# Transition-metal-free intramolecular Friedel–Crafts reaction by alkene activation: A method for the synthesis of some novel xanthene derivatives

**DOI:** 10.3762/bjoc.17.142

**Published:** 2021-08-30

**Authors:** Tülay Yıldız, İrem Baştaş, Hatice Başpınar Küçük

**Affiliations:** 1 Istanbul University-Cerrahpaşa, Department of Chemistry, Istanbul, Avcilar, 34320, Turkey

**Keywords:** alkene activation, intramolecular Friedel–Crafts alkylation, trifluoroacetic acid, xanthene

## Abstract

In this work, new derivatives (substituted 9-methyl-9-arylxanthenes) of xanthene compounds (**5a–l**) of possible biological significance were synthesized by developing a new synthesis method. In order to obtain xanthene derivatives, the original alkene compounds to be used as the starting materials were synthesized in four steps using appropriate reactions. A cyclization reaction by intramolecular Friedel–Crafts alkylation was carried out in order to synthesize the desired xanthene derivatives using the alkenes as starting compounds. The intramolecular Friedel–Crafts reaction was catalyzed by trifluoroacetic acid (TFA) and provided some novel substituted 9-methyl-9-arylxanthenes with good yields at room temperature within 6–24 hours. As a result, an alkene compound was used for activation with TFA in the synthesis of xanthene through intramolecular Friedel–Crafts alkylation for the first time.

## Introduction

The interest in xanthenes has increased remarkably in recent years due to their wide range of biological and pharmacological properties. Xanthenes are important biologically active oxygen-containing heterocyclic compounds. These types of compounds have some biological and pharmacological properties, such as anticarcinogenic [1–2], antiviral [3], antibacterial [4–5], and anti-inflammatory [6]. Because of their pharmaceutical activity, they are used in photodynamic therapy [7–9]. In addition, they have an important place in the dye industry due to their photochemical and photophysical properties. They are also used in pH-sensitive fluorescent materials [10] and laser technologies [11–14]. Moreover, xanthenes are present in some natural sources, such as santalin pigments found in many varieties of plants [15–16].

There have been many studies on the synthesis of xanthene derivatives to date because of their important biological and fluorescent uses. To summarize the main syntheses of these studies: in particular, transition metal-catalyzed cascade benzylation–cyclization [17], cyclization of polycyclic aryl triflate esters [18], reaction of β-naphthol and aldehydes [19–20] or inter- or intramolecular coupling of arynes by aldehydes or phenols [21–24], and Lewis acid-catalyzed cyclization of salicylaldehydes and cyclohexenones or tetralones [25]. Some other new and prominent synthesis methods of xanthenes are the tandem arylation/Friedel–Crafts reaction of *o*-hydroxy bisbenzylic alcohols with diaryliodonium salts [26], the Sc(OTf)_3_-catalyzed domino reaction [27], and the iodine-catalyzed nucleophilic substitution reaction of xanthen-9-ol [28]. As an example of an intramolecular hydroarylation of an olefin, 9,10-dihydroacridines, which are N derivatives of xanthenes, were synthesized using the combination of hexafluoroisopropanol and triflimide as a catalyst [29].

One of the most effective C–C bond establishing reactions, which ensures an efficient synthetic way to a plenty of functionalized aryl compounds, is the Friedel–Crafts cyclization reaction [30–34]. Therefore, there has been an increase in arylation methods using Friedel–Crafts alkylation (FCA) protocols without transition metals [35–36]. Environmentally friendly methods, in which metal-free catalysts are used, have come to the fore and are gaining importance because there are many disadvantages of organometallic chemistry and using transition metal catalysts, which need expensive and toxic chemicals. With the increasing interest in these methods, new organocatalysts and reagents that are less toxic, easier to use, readily accessible, and cheap have been developed [37]. In particular, the synthesis of arenes using the FCA methods with π-activated alcohols and organocatalysts has begun to be preferred over using conventional reagents, such as organohalogens and transition metal catalysts, which are toxic and require working under harsher conditions [38]. After these developments, the intramolecular FCA method using π-activated alcohols has frequently been used for xanthene synthesis. Some of these methods are the stereoselective synthesis of 9-vinyl-substituted unsymmetrical xanthenes and thioxanthenes by intramolecular FCA reaction [39], Lewis acid-catalyzed intramolecular FCA [40], and the synthesis of xanthenes and thioxanthenes by intramolecular FCA catalyzed with organic Brønsted superacid, which are our works [41–42]. According to the literature, there are also hydroarylation methods with FCA, which are made by unactivated alkenes instead of π-activated alcohols. In these studies, generally gold(III) [43], iridium(III) [44–45], iron(III) [46], or bismuth(III) [47–48] were used as catalysts for the intermolecular hydroarylation of unactivated alkenes. Organic Brønsted acids were also used as catalysts in a smaller number of studies [49–50].

In this work, we searched for some organic Brønsted acids and Lewis acids as catalysts (Table 1) to develop an intramolecular FCA protocol with activating alkenes effectively and economically in order to obtain some originally substituted arylxanthenes under mild conditions for the first time. We found that, among these acids, trifluoroacetic acid (TFA) was the best and most appropriate catalyst for this reaction. According to the literature, different from our work, TFA was reported as a catalyst for FCA with 6-acetoxy-4-alkenylarenes and benzyl alcohols in some previous studies [51–52].

As a continuation of our series of works to develop organic Brønsted acid-catalyzed cyclization reactions [41–42], herein we report a highly efficient intramolecular FCA of appropriate unactivated alkenes with a polyaromatic structure in order to synthesize xanthene derivatives. We developed a new intramolecular FCA method by activating alkenes working under mild reaction conditions and have widened the substrate scope of alkenes to those containing varied electronic and steric properties.

## Results and Discussion

**Scheme 1:**
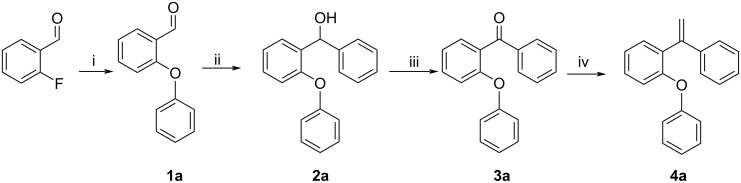
Synthesis of **4a:** (i) phenol, K_2_CO_3_, DMF, reflux, 2 h, 91%; (ii) PhMgBr, dry THF, 0 °C, 2 h, 86%; (iii) PCC, CH_2_Cl_2_, rt, 2 h, 80%; (iv) Me(Ph)_3_PBr, *t*-BuOK, NaH, dry THF, rt, 3 h, 85%.

Our starting alkenes **4a**–**l** are original and were synthesized in four steps involving coupling, Grignard, oxidation, and Wittig reactions. We synthesized the novel unactivated alkenes **4a**–**l** containing three aryl groups as the starting materials. The synthesis of **4a** is demonstrated in Scheme 1. First, 2-phenoxybenzaldehyde (**1a**) was synthesized by coupling reaction of phenol with commercial 2-fluorobenzaldehyde. This reaction was carried out with very high yield by refluxing the reactants in the presence of K_2_CO_3_ in DMF.

2-Phenoxybenzaldehyde (**1a**) was converted into secondary alcohol derivative **2a** by adding a phenyl group using a Grignard reaction. This reaction was carried out with high yield by adding the freshly prepared Grignard compound of phenyl bromide to 2-phenoxybenzaldehyde. As a result, phenoxy secondary alcohol **2a** containing three aromatic rings was obtained. In the third step, **2a** was oxidized and the ketone derivative **3a** was obtained in high yield. The oxidation reaction was carried out using PCC in DCM at room temperature. In the fourth and final step, phenoxydiphenylalkene derivative **4a** was prepared by a Wittig reaction, which was carried out using methyltriphenylphosphonium bromide with ketone **3a**, in basic medium, at room temperature, and dry THF.

After the structures of the starting compounds were elucidated, the method development trials for the synthesis of xanthene derivatives were carried out. For this purpose, catalyst researches were carried out using compound **4a**. An intramolecular Friedel–Crafts reaction was tried by activating the alkene with various organic Brønsted acids and Lewis acids (Table 1). In the reaction, iron(III) chloride hexahydrate, trifluoroacetic acid (TFA), *N*-trifylphosphoramide (NTPA), benzoic acid, diphenyl phosphate (DPP), malonic acid, chloroacetic acid, copper(II) triflate, acetic acid, and *p*-toluenesulfonic acid (*p*-TSA) were used as catalysts. TFA gave the best yield of these catalysts with 78% (Table 1, entry 2). The second-best yield was 65% when FeCl_3_·6H_2_O was used (Table 1, entry 1).

**Table 1:** Screening of catalysts for intramolecular FCA of **1a**.^a^

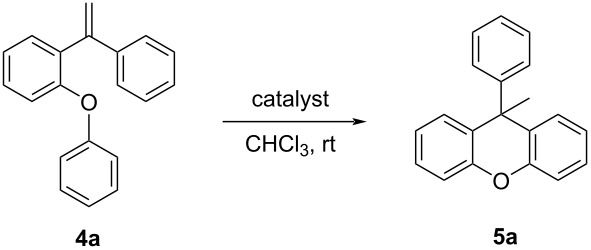		

Entry	Catalyst (10 mol %)	Conv. (%)^b^

1	FeCl_3_·6H_2_O	65
**2**	**TFA**	**78**
3	NTPA	10
4	benzoic acid	0
5	DPP	2
6	malonic acid	0
7	chloroacetic acid	14
8	Cu(OTf)_2_	12
9	AcOH	0
10	*p*-TSA	3

^a^Conditions: **4a** (0.1 mmol) and catalyst (10 mol %) in CHCl_3_ (1 mL) were stirred at room temperature for 24 hours. ^b^Conversions were determined with GC–MS.

Then the solvent was investigated. Toluene, methyl alcohol, ethyl acetate, THF, DMF, dichloromethane, chloroform, acetone, and acetonitrile were tested as solvents. As a result, it was determined that the best conversion was with dichloromethane (Table 2). Later, quantity and time experiments were performed (Table 2, entries 10–15) and, at the end of these trials, it was determined that the reaction was completed with >99% conversion in 6 hours with 10 mol % catalyst at room temperature (Table 2, entry 14).

**Table 2:** Exploration of solvents for intramolecular FCA of **1a**.^a^

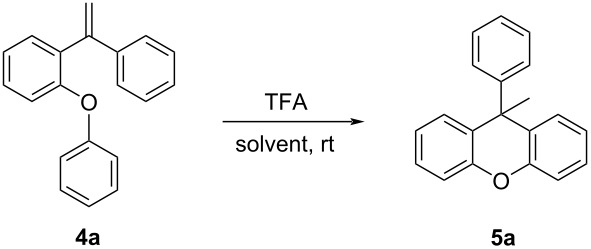				

Entry	Cat. amount (mol %)	Solvent	Time	Conv.^b^

1	10	CHCl_3_	24 h	78
2	10	acetone	24 h	75
3	10	toluene	24 h	70
4	10	CH_2_Cl_2_	24 h	>99
5	10	THF	24 h	45
6	10	CH_3_CN	24 h	10
7	10	MeOH	24 h	0
8	10	EtAc	24 h	35
9	10	DMF	24 h	0
10	10	CH_2_Cl_2_	1 h	32
11	10	CH_2_Cl_2_	2 h	53
12	10	CH_2_Cl_2_	3 h	70
13	10	CH_2_Cl_2_	4 h	85
**14**	**10**	**CH** **_2_** **Cl** **_2_**	**6 h**	**>99**
15	5	CH_2_Cl_2_	6 h	95

^a^Conditions: **4a** (0.1 mmol) and TFA in dry solvent (1 mL) were stirred at room temperature. ^b^Conversions were determined with GC–MS.

**Figure 1:**
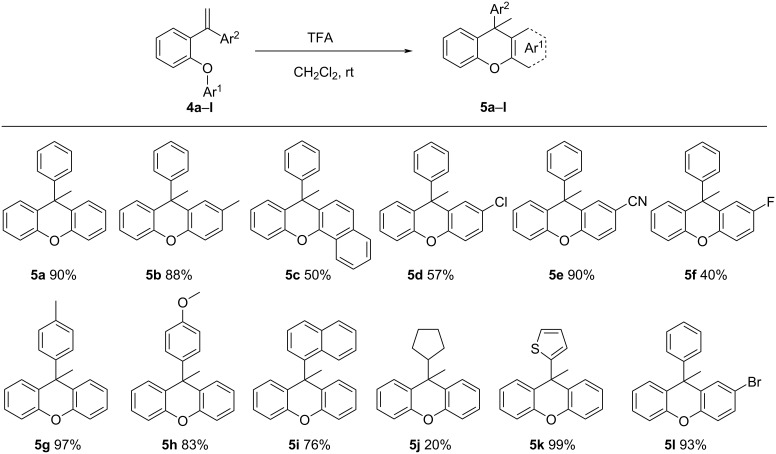
Scope of substrates for intramolecular FCA by activation of **4a–l** and their isolated yields. ^a^Conditions: **4a–l** (0.1 mmol) and TFA (10 mol %) in dry CH_2_Cl_2_ (1 mL) were stirred at room temperature for 1–24 hours. ^b^Yield of isolated product.

After determining the most suitable conditions for the intramolecular Friedel–Crafts reaction with alkene activation, the synthesis of the new xanthene derivatives was performed according to this method. The synthesized xanthene derivatives with their isolated yields are shown in Figure 1. Compounds **5b**–**l** were synthesized for the first time in this study. The first synthesis of **5a** was prepared by reduction of the corresponding xanthydrol [53] and was also synthesized recently by a different method from our group [29].

**Scheme 2:**
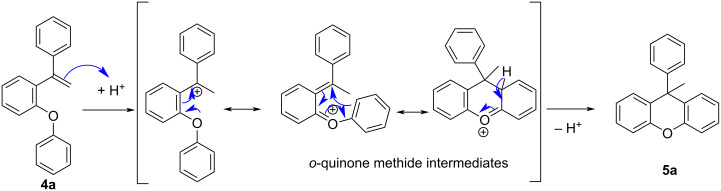
Plausible reaction mechanism for the cyclization reaction of alkene **4a**.

Although in the reactions for FCA reagents and strong inorganic acids, such as AlCl_3_, H_2_SO_4_, or H_3_PO_4_, which have generally corrosive properties, were used, in this study, an intramolecular ring closure reaction was carried out under easy operating conditions with an organic Brønsted acid catalyst with high yields. So, the xanthene synthesis with alkene activation was performed for the first time using TFA. The reasonable mechanism of this reaction is delineated in Scheme 2.

Despite this reaction occurring by the classical Friedel–Crafts mechanism, we believe that *o*-quinone methide is formed as an intermediate. Because of its very reactive structure, most of the xanthene synthesis is based on the *o*-quinone methide intermediate [54–58]. The carbocation formed by the activation of an alkene with acid turns into an intermediate *o*-quinone methide, resulting in a successful cyclization.

As seen in the mechanism, the acid catalyst adds to the vinyl group, allowing the formation of a tertiary carbocation. The carbocation is then transformed into the *o*-quinone methide intermediate, which undergoes cyclization to yield 9-methyl-9-arylxanthene by aromatization. When the yields of the synthesized compounds are examined, it is seen that the yields are high when there is no substituent at the ring to which the carbocation is attached or when there is an electron-donating group, such as a methoxy or methyl group (Figure 1). When there are electron-withdrawing groups, such as chlorine or cyano groups, in the ring, the yield is slightly reduced because they deactivate the ring in the transition state. The lowest yield was observed for compound **5j** to which a cyclopentyl group is attached. Since the cyclopentyl group in this compound is directly attached to the carbon from which the carbocation is formed, byproducts are formed and the yield is reduced since conversions can occur.

## Conclusion

In this study, a novel transition metal-free FCA method for the synthesis of 9-methyl-9-arylxanthenes has been developed. For this method, starting compounds with alkene structures suitable for our targets were synthesized. Among the synthesis methods of xanthene derivatives in the literature, the intramolecular Friedel–Crafts method, in which alkenes are activated, has not been used before. Remarkably, our mild reaction conditions using highly effective, inexpensive, and non-toxic reagents are very suitable for the efficient synthesis of xanthenes. The substituted 9-methyl-9-arylxanthenes **5a**–**l** may be used in the pharmaceutical chemistry field as native and bioactive products, and their biochemical potentials and efficiencies will be investigated in the coming years because of their potential biological uses, such as antileukemic, antifungal, antimycobacterial, antitumor, antioxidant, and anticarcinogenic.

## Experimental

### General information

The majority of the chemicals used in this work were commercially available from Merck or Aldrich. The starting compounds **1a**–**l** were prepared by Ullmann coupling of 2-fluorobenzaldehyde and substituted phenols. Compounds **2a**–**l** were synthesized by Grignard reaction of **1a**–**l** and aryl(or alkyl)magnesium bromides. Then **3a**–**l** were prepared by oxidation of **2a**–**l** using PCC. The final alkene compounds **4a**–**l** were obtained by Wittig reaction using Me(Ph)_3_PBr, *t*-BuOK, and NaH. All substrates were purified by crystallization or column chromatography and were characterized by IR and GC–MS. All novel pruducts were characterized by IR, ^1^H NMR, ^13^C NMR, elemental analysis and GC–MS. The reactions were monitored by TLC using silica gel plates and the products were purified by flash column chromatography on silica gel (Merck; 230–400 mesh), eluting with hexane/ethyl acetate (v/v 9:1). NMR spectra were recorded at 500 MHz for ^1^H and 125 MHz for ^13^C using Me_4_Si as the internal standard in CDCl_3_. GC–MS were recorded on a Shimadzu/ QP2010 Plus spectrometer. IR spectra were recorded on a Mattson 1000 spectrometer. Melting points were determined with a Büchi Melting Point B-540 apparatus.

## Supporting Information

File 1 Experimental and analytical data.

## References

[1] Song Y, Yang Y, You J, Liu B, Wu L, Hou Y, Wang W, Zhu J (2013). Chem Pharm Bull.

[2] Lin C-N, Liou S-J, Lee T-H, Chuang Y-C, Won S-J (1996). J Pharm Pharmacol.

[3] Poupelin J P, Saint-Ruf G, Foussard-Blanpin O, Narcisse G, Uchida-Ernouf G, Lacroix R (1978). Eur J Med Chem.

[4] Barmak A, Niknam K, Mohebbi G, Pournabi H (2019). Microb Pathog.

[5] Akbari A, Hosseini-Nia A (2017). J Saudi Chem Soc.

[6] Lambert R W, Martin J A, Merrett J H, Parkes K E B, Thomas G J (1997). PCT Int. Appl..

[7] Banerjee A, Mukherjee A K (1981). Stain Technol.

[8] Ion R-M (1997). Prog Catal.

[9] Ion R M, Planner A, Wiktorowicz K, Frackowiak D (1998). Acta Biochim Pol.

[10] Knight C G, Stephens T (1989). Biochem J.

[11] Gonçalves M S T (2009). Chem Rev.

[12] Feringa B L (2007). J Org Chem.

[13] Tisseh Z N, Azimi S C, Mirzaei P, Bazgir A (2008). Dyes Pigm.

[14] Hirata R, Torii A, Kawano K, Futaki S, Imayoshi A, Tsubaki K (2018). Tetrahedron.

[15] Kinjo J, Uemura H, Nohara T, Yamashita M, Marubayashi N, Yoshihira K (1995). Tetrahedron Lett.

[16] Robertson L P, Lucantoni L, Duffy S, Avery V M, Carroll A R (2019). J Nat Prod.

[17] Xu X, Xu X, Li H, Xie X, Li Y (2010). Org Lett.

[18] Wang J-Q, Harvey R G (2002). Tetrahedron.

[19] Su W, Yang D, Jin C, Zhang B (2008). Tetrahedron Lett.

[20] Naidu K R M, Krishna B S, Kumar M A, Arulselvan P, Khalivulla S I, Lasekan O (2012). Molecules.

[21] Zhao J, Larock R C (2005). Org Lett.

[22] Okuma K, Nojima A, Matsunaga N, Shioji K (2009). Org Lett.

[23] Yoshida H, Watanabe M, Fukushima H, Ohshita J, Kunai A (2004). Org Lett.

[24] Zhao J, Larock R C (2007). J Org Chem.

[25] Böβ E, Hillringhaus T, Nitsch J, Klussmann M (2011). Org Biomol Chem.

[26] Mao S, Hua Z, Wu X, Yang Y, Han J, Wang L (2016). ChemistrySelect.

[27] Singh R, Panda G (2010). Org Biomol Chem.

[28] Miao W, Ye P, Bai M, Yang Z, Duan S, Duan H, Wang X (2020). RSC Adv.

[29] Wang S, Force G, Carpentier J-F, Sarazin Y, Bour C, Gandon V, Lebœuf D (2021). Org Lett.

[30] Zhu Y-Q, Dong L (2015). J Org Chem.

[31] Liu C-R, Yang F-L, Jin Y-Z, Ma X-T, Cheng D-J, Li N, Tian S-K (2010). Org Lett.

[32] Kozak J A, Patrick B O, Dake G R (2010). J Org Chem.

[33] Wang S, Zhu Y, Wang Y, Lu P (2009). Org Lett.

[34] Zhou X, Zhang H, Xie X, Li Y (2008). J Org Chem.

[35] Shirakawa E, Itoh K-i, Higashino T, Hayashi T (2010). J Am Chem Soc.

[36] Yanagisawa S, Ueda K, Taniguchi T, Itami K (2008). Org Lett.

[37] Deng X, Liang J T, Liu J, McAllister H, Schubert C, Mani N S (2007). Org Process Res Dev.

[38] Bandini M, Tragni M (2009). Org Biomol Chem.

[39] Prajapati A, Kumar M, Thakuria R, Basak A K (2020). Tetrahedron Lett.

[40] Das S K, Singh R, Panda G (2009). Eur J Org Chem.

[41] Yıldız T, Küçük H B (2017). RSC Adv.

[42] Yildiz T (2018). Synth Commun.

[43] Xiao Y-P, Liu X-Y, Che C-M (2009). J Organomet Chem.

[44] Matsumoto T, Periana R A, Taube D J, Yoshida H (2002). J Mol Catal A: Chem.

[45] Bhalla G, Oxgaard J, Goddard W A, Periana R A (2005). Organometallics.

[46] Kischel J, Jovel I, Mertins K, Zapf A, Beller M (2006). Org Lett.

[47] Rueping M, Nachtsheim B J, Scheidt T (2006). Org Lett.

[48] Sun H-B, Li B, Hua R, Yin Y (2006). Eur J Org Chem.

[49] Bonderoff S A, West F G, Tremblay M (2012). Tetrahedron Lett.

[50] Fleischer I, Pospech J (2015). RSC Adv.

[51] Ma S, Zhang J (2003). Tetrahedron.

[52] Ladd A L, Gribble G W (2018). Biomed J Sci Tech Res.

[53] Lambelet P, Lucken E A C (1975). J Chem Soc, Perkin Trans 2.

[54] Karthick M, Konikkara Abi E, Someshwar N, Anthony S P, Ramanathan C R (2020). Org Biomol Chem.

[55] Huang C-G, Shukla D, Wan P (1991). J Org Chem.

[56] Yoshida H, Watanabe M, Fukushima H, Ohshita J, Kunai A (2004). Org Lett.

[57] Hsiao C-C, Liao H-H, Rueping M (2014). Angew Chem, Int Ed.

[58] Hsiao C-C, Raja S, Liao H-H, Atodiresei I, Rueping M (2015). Angew Chem, Int Ed.

