# Supplementary material for: Transition-metal-free intramolecular Friedel–Crafts reaction by alkene activation: A method for the synthesis of some novel xanthene derivatives
Source: Beilstein J Org Chem. 2021 Aug 30;17:2203–8. doi: 10.3762/bjoc.17.142 (PMC8450977; doi:10.3762/bjoc.17.142)
Supplement: File 1 — Experimental and analytical data. [file Beilstein_J_Org_Chem-17-2203-s001.pdf]

BEILSTEIN JOURNAL OF ORGANIC CHEMISTRY

Supporting Information

for

Transition-metal-free intramolecular Friedel–Crafts reaction by alkene activation: A method for the synthesis of some novel xanthene derivatives

Tülay Yıldız, İrem Baştaş and Hatice Başpınar Küçük

Beilstein J. Org. Chem. **2021,** *17,* 2203–2208. doi:10.3762/bjoc.17.142

Experimental and analytical data

License and Terms: This is a supporting information file under the terms of the Creative Commons Attribution License (https://creativecommons.org/licenses/by/4.0). Please note that the reuse, redistribution and reproduction in particular requires that the author(s) and source are credited and that individual graphics may be subject to special legal provisions.
The license is subject to the *Beilstein Journal of Organic Chemistry* terms and conditions: (https://www.beilstein-journals.org/bjoc/terms)

Experimental

1.General information

The majority of the chemicals used in this work were commercially available from Merck or Aldrich. The starting compounds **1a–l** were prepared by Ullmann coupling of 2-fluoro benzaldehyde and substituted phenols. The further starting compounds **2a–l** were synthesized Grignard reaction of **1a–l** and aryl(or alkyl)magnesium bromide. Then **3a–l** were prepared from oxidation of **2a–l** using PCC. The final starting alkene compounds **4a–l** were obtained with Wittig reaction using $Me(Ph)_3PBr$, *t*-BuOK, NaH. All substrates were purified by crystallization or column chromatography and were characterized by IR and GC-MS. All novel pruducts were characterized by IR, ^{1}H NMR, ^{13}C NMR, elemental analysis and GC-MS. The reactions were monitored by TLC using silica gel plates and the products were purified by flash column chromatography on silica gel (Merck; 230–400 mesh), eluting with hexane-ethyl acetate (v/v 9:1). NMR spectra were recorded at 500 MHz for ^{1}H and 125 MHz for ^{13}C using Me_4Si as the internal standard in $CDCl_3$. GC-MS were recorded on Shimadzu/ QP2010 Plus. IR spectra were recorded on a Mattson 1000 spectrometer. Melting points were determined with Büchi Melting Point B-540.

2. General procedure for reduction of the Ullmann coupling:[1]

To a solution of DMF (10 mL) containing 2-fluorobenzaldehyde (4 mmol) and phenol (4 mmol) was added K_2CO_3 (6 mmol) and the reaction mixture was stirred for 2 hours at 175 °C. It was cooled to room temperature and after usual workup and concentration, the product was purified over silica gel.
Thus, 2- arenoxybenzaldehydes were prepared in good yields with 85–95% (Scheme S1).

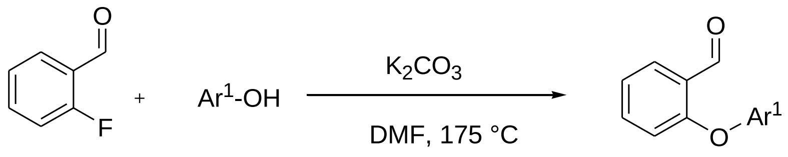

1a-1l
85-95 % yield

Scheme S1. The Ullmann type reaction of 2-fluorobenzaldehyde and hydroxy aryls.

3.General procedure for the Grignard reaction:

For the Grignard solution, to Mg (2 mmol) was added the respective bromo substrate (1.2 mmol) in dry THF and the mixture was heated under reflux and N_2 for 0,5–1 h then cooled to room temperature. 2-Arenoxybenzaldehydes (1 mmol) was dissolved in dry THF and cooled to 0 °C. Then the Grignard solution was added to the aldehyde solution and stirred for 1 hour. The end of the reaction was quenched with saturated NH_4Cl solution, extracted with DCM and water 2 times. After usual workup and concentration, the crude product was purified over silica gel.

Diarylcarbinol compounds including an arenoxy group were obtained with very high yields (85–90%) (Scheme S2).

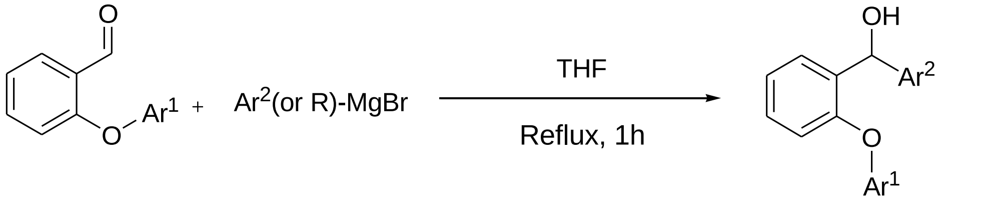

2a-2l
85-90 % yield

Scheme S2. The Grignard reaction of 2-arenoxybenzaldehyde.

4. General procedure for the alcohol oxidation:

To the alcohol compound (1 mmol) were added PCC (1.5 mmol) and DCM (5 mL) as solvent then stirred for 2 hours. The end of the reaction was extracted with DCM and water 2 times. After usual reaction workup and concentration, the product was charged on silica gel (Scheme S3).

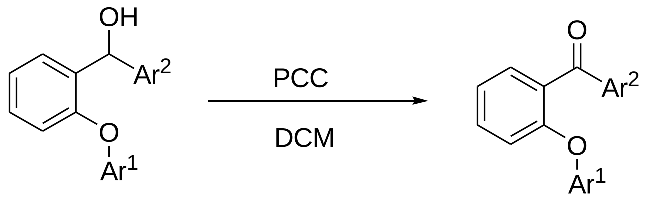

2a-2l

3a-3l
85-95 % yield

Scheme S3. The oxidation of diarylcarbinol compounds.

5. General procedure for the Wittig reaction:

To the ketone compound (1 mmol) in dry THF were added $Me(Ph)_3PBr$ (2.2 mmol), *t*-BuOK (2.7 mmol) and NaH (2.5 mmol) and the reaction was stirred for 1-2 hours. At the end of the reaction it was extracted with DCM and water 2 times. After usual reaction workup and concentration, the product was charged on silica gel (Scheme S4).

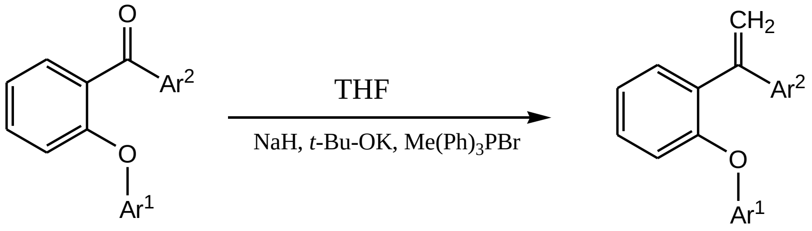

3a-3l

4a-4l
50-60 % yield

Scheme S4. Wittig reaction of ketones **3a–3l.**

6. General procedure for intramolecular FCA of vinyl compounds

TFA (10 mol %) was added to a stirred solution of a starting alkene compound (**4a**–**4l**) (0.1 mmol) in DCM (1 mL), and the reaction was stirred for 6-24 h under room temperature. After the finishing of the reaction, as monitored with TLC the reaction mixture was washed with water two times. After the usual reaction workup and concentration, the product was charged on silica gel.

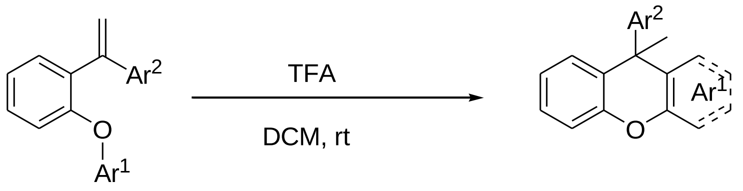

4a-4l **5a-5l**

Scheme S5. The intramolecular FCA of vinyl compounds **4a–4l.**

7.Experimental and characterization data of 4a–l and 5a–l

***1-Phenoxy-2-(1-phenyl-vinyl)benzene* (4a):**

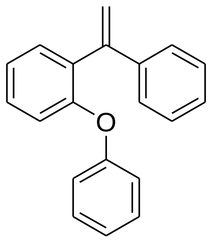

Colorless oily (85% yield).^{1}**H NMR** (500 MHz, $CDCl_3$) δ 5.37 (d, *J* = 1.5 Hz, 1H), 5.63 (d, *J* = 1.5 Hz, 1H), 6.72 (d, *J* = 8.5 Hz, 1H), 6.96 − 7.01 (m, 2H), 7.17 − 7.40 (m, 10H). ^{13}**C NMR** (150MHz, $CDCl_3$) δ 116.3, 117.9, 117.8, 119.9, 122.4, 123.7, 126.7, 127.3, 127.9, 129.0, 129.1, 129.2, 131.7, 134.0, 141.0, 146.3, 154.0, 157.4. **MS** (m/z) = 152, 165, 181, 194, 272 (M^+). Anal. Calcd. for $C_{20}H_{16}O$: C, 88.20; H, 5.92. Found: C, 88.33; H, 5.93.

***1-[4-Methylphenoxy]-2-(1-pheny-vinyl)benzene* (4b):**

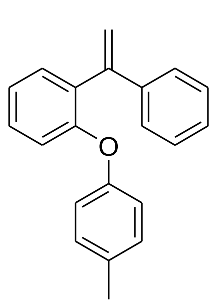

Colorless oily (53% yield). ^{1}**H NMR** (500 MHz, $CDCl_3$) δ 2.30 (s, 3H), 5.40 (d, *J* = 1.5 Hz, 1H), 5.67 (d, *J* = 1.5 Hz, 1H), 6.66 (d, *J* = 8.5 Hz, 2H), 6.92 (d, *J* = 9.0 Hz, 1H), 6.95 − 7.18 (m, 4H), 7.24 − 7.39 (m, 6H). ^{13}**C NMR** (150MHz, $CDCl_3$) δ 20.6, 116.1, 118.2, 118.3, 119.1, 119.1, 123.2, 126.7, 127.3, 128.0, 128.9, 129.8, 130.2, 131.6, 132.1, 133.6, 141.1, 146.4, 154.6, 155.0. **MS** (m/z) = 77, 105, 211, 288 (M^+). Anal. Calcd. for $C_{21}H_{18}O$: C, 88.08; H, 6.34. Found: C, 88.23; H, 6.26.

2-[2-(1-Phenylvinyl)phenoxy]naphthalene **(4c):**

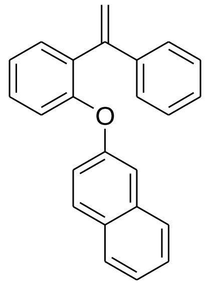

Pale yellow oily (57% yield) . **^{1}H NMR** (500 MHz, $CDCl_3$) δ 5.40 (d, J = 1.5 Hz, 1H), 5.62 (d, J = 1.5 Hz, 1H), 6.97 (dd, J_1 = 2.5 Hz, J_2 = 9.0 Hz, 1H), 7.05 − 7.07 (m, 1H), 7.24 − 7.32 (m, 5H), 7.35 − 7.49 (m, 6H), 7.63 − 7.88 (m, 3H). **^{13}C NMR** (150MHz, $CDCl_3$) δ 112.7, 114.1, 116.4, 119.1, 119.3, 120.0, 123.3, 123.4, 123.4, 124.0, 124.2, 126.3, 126.7, 127.6, 128.0, 129.1, 129.4, 129.8, 131.8, 134.1, 141.0, 146.3, 153.8, 155.3. **MS** (m/z) = 194, 215, 321, 322 (M^+). Anal. Calcd. for $C_{24}H_{18}O$: C, 89.41; H, 5.63. Found: C, 89.56; H, 5.54.

1-[4-Chlorophenoxy]-2-(1-phenylvinyl)benzene **(4d):**

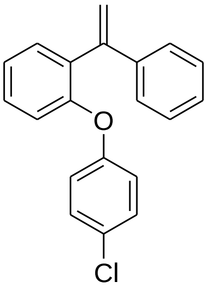

Colorless oily (64% yield). **^{1}H NMR** (500 MHz, $CDCl_3$) δ 5.34 (d, J = 1.5 Hz, 1H), 5.62 (d, J = 1.5 Hz, 1H), 6.62 (d, J = 9.0 Hz, 1H), 6.96 − 6.99 (m, 1H), 7.15 (d, J = 9.0 Hz, 2H), 7.22 − 7.43 (m, 9H). **^{13}C NMR** (150MHz, $CDCl_3$) δ 116.5, 118.8, 118.9, 120.0, 120.2, 123.6, 124.3, 126.6, 127.2, 127.5, 128.0, 129.2, 129.7, 129.8, 131.8, 134.2, 140.9, 146.3, 153.4, 456.1. **MS** (m/z) = 152, 165, 181, 194, 306 (M^+). Anal. Calcd. for $C_{20}H_{15}ClO$: C, 78.30; H, 4.93. Found: C, 78.48; H, 4.86.

4-[2-(1-Phenylvinyl)phenoxy]benzonitrile **(4e):**

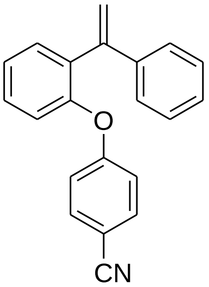

Yellow oily (45% yield). **^{1}H NMR** (500 MHz, $CDCl_3$) δ 5.29 (d, J = 1.0 Hz, 1H), 5.55 (d, J = 1.0 Hz, 1H), 6.65 (d, J = 1.0 Hz, 2H), 7.06 (dd, J_1 = 1.5 Hz, J_2 = 8.0 Hz, 1H), 7.13 − 7.16 (m, 2H), 7.21 − 7.27 (m, 3H), 7.32 (dd, J_1 = 1.0 Hz, J_2 = 7.5 Hz, 1H), 7.40 − 7.48 (m, 4H). **^{13}C NMR** (150MHz, $CDCl_3$) δ 105.0, 116.9, 117.9, 118.9, 120.4, 121.8, 125.7, 126.6, 127.6, 128.0, 129.5, 130.2, 132.1, 133.6, 134.1, 135.0, 140.5, 146.0, 151.4, 161.2. **MS (m/z) =** 152, 165, 181, 194, 296 (M^+). Anal. Calcd. for $C_{21}H_{15}NO$: C, 84.82; H, 5.08; N, 4.71. Found: C, 84.75; H, 5.19; N, 4.54.

1-[4-Fluorophenoxy]-2-(1-pheny-vinyl)benzene **(4f):**

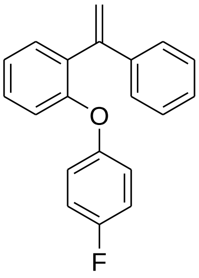

Colorless oily (43% yield). **^{1}H NMR** (500 MHz, $CDCl_3$) δ 5.36 (s, 1H), 5.65 (s, 1H), 6.66 (dd, J_1 = 4.5 Hz, J_2 = 9.0 Hz, 1H), 6.88 − 6.92 (m, 2H), 6.99 − 7.07 (m, 2H), 7.21 − 7.27 (m, 1H), 7.25 − 7.41 (m, 7H). **^{13}C NMR** (150MHz, $CDCl_3$) δ 115.6 (d, $^2J_{CF}$= 27.9 Hz), 115.8 (d, $^2J_{CF}$= 27.9 Hz),

116.2, 118.2, 119.2 (d, ${}^{3}J_{CF}$= 6.6 Hz), 119.3 (d, ${}^{3}J_{CF}$= 6.6 Hz), 120.5, 120.5, 123.1, 123.7, 126.6, 127.4, 128.0, 129.1, 129.7, 131.8, 140.9, 146.4, 154.3- 159.2 (d, ${}^{1}J_{CF}$= 287.1 Hz). **MS** (m/z) = 152, 165, 181, 194, 290 (M^+). Anal. Calcd. for $C_{20}H_{15}FO$: C, 82.74; H, 5.21. Found: C, 82.81; H, 5.29.

***1-Phenoxy-2-(1-4-methylphenylvinyl)benzene* (4g):**

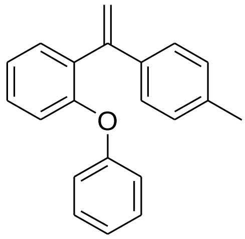

Colorless oily (66% yield). **^{1}H NMR** (500 MHz, $CDCl_3$) δ 2.37 (s, 3H), 5.34 (d, J = 1.5 Hz, 1H), 5.64 (d, J = 1.5 Hz, 1H), 6.81 (d, J = 8.5 Hz, 2H), 7.00 − 7.05 (m, 2H), 7.11 − 7.12 (m, 2H), 7.19 − 7.28 (m, 5H), 7.28 − 7.33 (m, 1H), 7.40 (dd, J_1 = 1.5 Hz, J_2 = 9.0 Hz, 1H). **^{13}C NMR** (150MHz, $CDCl_3$) δ 21.1, 115.5, 117.9, 118.9, 120.0, 122.4, 123.7, 126.6, 128.7, 128.9, 129.3, 129.7, 131.7, 134.3, 137.1, 138.2, 146.0, 154.0, 157.6. **MS (m/z) =** 165, 178, 195, 271, 286 (M^+). Anal. Calcd. for $C_{21}H_{18}O$: C, 88.08; H, 6.35. Found: C, 88.12; H, 6.29.

***1-Phenoxy-2-(1-4-methoxyphenylvinyl)benzene* (4h):**

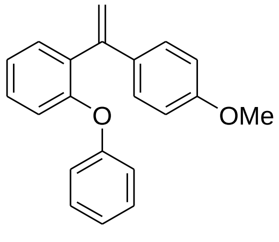

Colorless oily (40% yield). **^{1}H NMR** (500 MHz, $CDCl_3$) δ 3.81 (s, 3H), 5.25 (d, J = 1.5 Hz, 1H), 5.56 (d, J = 1.5 Hz, 1H), 6.80 (d, J = 9.0 Hz, 2H), 7.01 − 7.06 (m, 2H), 7.17 − 7.24 (m, 5H), 7.31 − 7.39 (m, 4H). **^{13}C NMR** (150MHz, $CDCl_3$) δ 55.2, 113.3, 114.6, 117.9, 118.8, 120.0, 122.4, 123.2, 123.7, 127.8, 128.9, 129.2, 129.7, 131.7, 133.6, 134.3, 145.6, 153.9, 157.2, 157.5, 159.1. **MS (m/z) =** 165, 211, 271, 287, 302 (M^+). Anal. Calcd. for $C_{21}H_{18}O_2$: C, 83.42; H, 6.00. Found: C, 83.52; H, 6.08.

***1-[1-(2-Phenoxyphenyl)vinyl]naphthalene* (4i):**

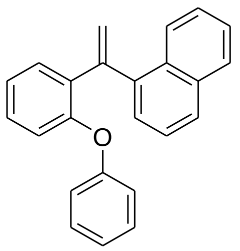

Orange oily (42% yield). **^{1}H NMR** (500 MHz, $CDCl_3$) δ 5.56 (d, J = 1.5 Hz, 1H), 5.98 (d, J = 1.5 Hz, 1H), 6.60 (d, J = 10.0 Hz, 2H), 6.92 − 7.49 (m, 14H). **^{13}C NMR** (150MHz, $CDCl_3$) δ 117.2, 118.8, 120.6, 120.8, 122.1, 124.0, 122.1, 124.0, 125.1, 125.3, 125.5, 126.1, 126.5, 127.4, 128.1, 128.8, 129.2, 129.7, 130.8, 144.9, 153.3, 157.4. **MS (m/z) =** 77, 115, 127, 155, 197, 231, 247, 322 (M^+). Anal. Calcd. for $C_{24}H_{18}O$: C, 89.41; H, 5.63. Found: C, 89.48; H, 5.69.

***1-(1-Cyclopentylvinyl)-2-phenoxybenzene* (4j):**

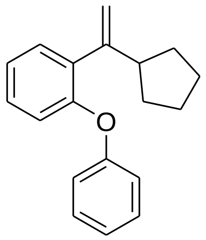

Colorless oily (51% yield). **^{1}H NMR** (500 MHz, $CDCl_3$) δ 1.27− 1.30 (m, 8H), 1.57− 1.58 (m, 1H), 5.28 (dd, J_1 = 1.5 Hz, J_2 = 11.0 Hz, 1H), 5.79 (dd, J_1 = 1.5 Hz, J_2 = 19.0 Hz, 1H), 6.92 − 7.28 (m, 9H). **^{13}C NMR** (150MHz, $CDCl_3$) δ 22.6, 29.7, 31.9, 115.3, 117.7, 120.0, 119.4, 122.6, 124.0, 126.6, 128.9, 129.6, 130.0, 130.9, 135.7, 153.5, 157.8. **MS** (m/z) = 77, 93, 115, 127,169, 171, 187, 195, 250, 264 (M^+). Anal. Calcd. for $C_{19}H_{20}O$: C, 86.32; H, 7.63. Found: C, 86.41; H, 7.71.

***2-[1-(2-Phenoxyphenyl)vinyl]thiophene* (4k):**

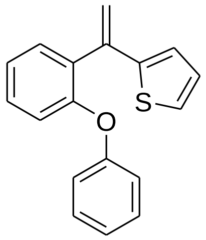

Pale brown oily (62% yield). . **^{1}H NMR** (500 MHz, $CDCl_3$) δ 5.19 (s, 1H), 5.70 (s, 1H), 6.82 (dd, J_1 = 1.0 Hz, J_2 = 3.5 Hz, 1H), 6.86 − 6.88 (m, 2H), 6.93 (dd, J_1 = 3.5 Hz, J_2 = 5.0 Hz, 1H), 6.98 (dd, J_1 = 1.0 Hz, J_2 = 8.0 Hz, 1H), 7.02 − 7.05 (m, 1H), 7.16 − 7.20 (m, 2H), 7.24 − 7.28 (m, 2H), 7.32 − 7.38 (m, 1H), 7.40 (dd, J_1 = 3.5 Hz, J_2 = 5.0 Hz, 1H). **^{13}C NMR** (150MHz, $CDCl_3$) δ 114.8, 118.2, 118.8, 119.6, 122.7, 123.5, 124.5, 125.5, 127.1, 129.2, 129.4, 129.7, 131.3, 133.1, 139.4, 144.9, 154.1, 157.5. **MS** (m/z) = 77, 83, 93, 127, 169, 171, 185, 201, 250, 264, 278 (M^+). Anal. Calcd. for $C_{18}H_{14}OS$: C, 77.66; H, 5.07; S, 11.52. Found: C, 77.58; H, 5.12; S, 11.42.

***1-[4-Bromophenoxy]-2-(1-phenylvinyl)benzene* (4l):**

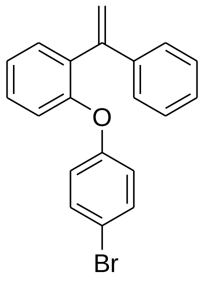

Yellow oily (54% yield). **^{1}H NMR** (500 MHz, $CDCl_3$) δ 5.32 (d, 1H, J = 1.5 Hz), 5.60 (d, 1H, J = 1.5 Hz), 6.55 (d, J = 9.0 Hz, 1H), 6.90 (d, J = 9.0 Hz, 1H), 6.93 − 7.03 (dd, J_1 = 15.5 Hz, J_2 = 9.0 Hz, 2H), 7.22 − 7.38 (m, 8H), 7.44 (d, J = 9.0 Hz, 1H). **^{13}C NMR** (150MHz, $CDCl_3$) δ 116.5, 119.0, 119.2, 120.4, 124.4, 126.5, 127.5, 128.8, 129.2, 129.8, 132.1, 132.6, 134.3, 140.8, 145.2, 153.2, 156.7. **MS** (m/z) =154, 170, 179, 195, 271, 336, 350 (M^+). Anal. Calcd. for $C_{20}H_{15}BrO$: C, 68.39; H, 4.30. Found: C, 68.51; H, 4.28.

***9-Methyl-9-phenyl-9H-xanthene* (5a):**[2,3]

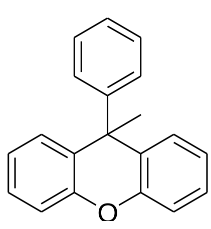

White solid (90% yield); mp: 105.3-106.8 °C. **IR** (cm^{-1}) ν = 3018, 2958, 1594, 1597, 1468, 1431, 1245, 1021. **^{1}H NMR** (400 MHz, $CDCl_3$) δ 1.96 (s, 3H), 6.87 (d, J_1 = 7.5 Hz, 2H), 6.96 (dd, J_1 = J_2 = 7.5 Hz, 2H), 7.12 (d, J_1 = 8.0 Hz, 2H), 7.19 − 7.38 (m, 7H). **^{13}C NMR** (150 MHz, $CDCl_3$) δ 32.1, 42.9, 116.1, 123.0, 126.2, 127.4, 127.8, 128.0, 128.6, 128.0, 130.0, 130.2, 149.0, 150.2.

MS (m/z) = 195, 257, 272 (M^+). Anal. Calcd. for $C_{20}H_{16}O$: C, 88.20; H, 5.92. Found: C, 88.12; H, 5.83.

2,9-Dimethyl-9-phenyl-9H-xanthene **(5b):**

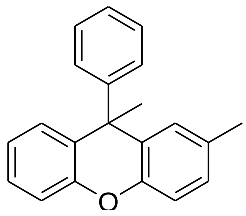

Colorless oily (88% yield). **IR** (cm^{-1}) ν = 3018, 2965, 2908, 1591, 1574, 1475, 1445, 1245, 750. **^{1}H NMR** (500 MHz, $CDCl_3$) δ 1.95 (s, 3H), 2.21 (s, 3H), 6.66 (d, *J* = 2.0 Hz, 1H), 6.85 (dd, J_1 = 1.5 Hz, J_2 = 8.0 Hz, 1H), 6.92 − 7.38 (m, 10H). **^{13}C NMR** (150MHz, $CDCl_3$) δ 20.8, 32.1, 42.9, 115.9, 116.1, 118.3, 119.1, 122.8, 126.1, 127.3, 128.0, 128.2, 128.6, 129.0, 129.0, 129.6, 130.2, 132.2, 148.1, 150.3, 150.3. **MS (m/z) =** 209, 286, 271 (M^+). Anal. Calcd. for $C_{21}H_{18}O$: C, 88.08; H, 6.34. Found: C, 88.13; H, 6.41.

12-Methyl-12-phenyl-12H-benzo[b]xanthene **(5c):**

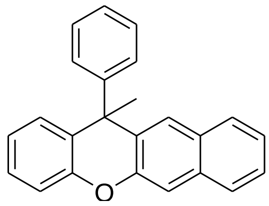

Beige solid (50% yield); mp: 137.8-140.9 °C. **IR** (cm^{-1}) ν = 3041, 3024, 2961, 2911, 1584, 1485, 1448, 1245, 743. **^{1}H NMR** (500 MHz, $CDCl_3$) δ 2.29 (s, 3H), 6.91 − 6.96 (m, 2H), 7.08 − 7.60 (m, 11H), 7.72 − 7.87 (m, 2H). **^{13}C NMR** (150MHz, $CDCl_3$) δ 30.3, 42.9, 114.0, 115.8, 118.1, 119.1, 119.9, 123.2, 123.4, 124.6, 125.6, 125.8, 126.0, 126.5, 127.1, 127.7, 127.8, 128.4, 128.9, 129.6, 131.4, 148.2, 150.1, 155.0. **MS (m/z) =** 245, 307, 308, 322 (M^+). Anal. Calcd. for $C_{24}H_{18}O$: C, 89.41; H, 5.63. Found: C, 89.78; H, 5.71.

2-Chloro-9-methyl-9-phenyl-9H-xanthene **(5d):**

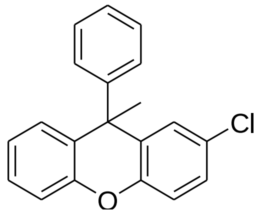

Colorless oily (57% yield). **IR** (cm^{-1}) ν = 3051, 3018, 2965, 2911, 1594, 1561, 1468, 1438, 1269, 1235, 750. **^{1}H NMR** (500 MHz, $CDCl_3$) δ 1.93 (s, 3H), 6.83 (d, *J* = 2.5 Hz, 1H), 6.84 (dd, J_1 = 2.0 Hz, J_2 = 7.5 Hz, 1H), 6.95 − 7.35 (m, 10H). **^{13}C NMR** (150MHz, $CDCl_3$) δ 32.1, 43.1, 116.1, 117.6, 118.9, 120.0, 123.3, 126.5, 127.6, 128.2, 128.5, 128.6, 128.9, 129.5, 129.7, 129.8, 131.0, 148.2, 148.8, 149.9. **MS (m/z) =** 51, 75, 152, 165, 181, 194, 271, 306 (M^+). Anal. Calcd. for $C_{20}H_{15}ClO$: C, 78.30; H, 4.93. Found: C, 78.41; H, 4.89.

9-Methyl-9-phenyl-9H-xanthene-2-carbonitrile **(5e):**

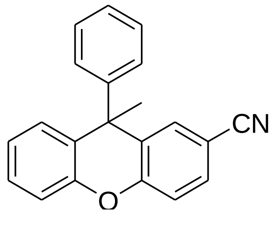

Yellow oily (90% yield). **IR** (cm^{-1}) ν = 3049, 2970, 2225, 1600, 1479, 1447, 1240, 1044. **^{1}H NMR** (500 MHz, $CDCl_3$) δ 1.93 (s, 3H), 7.01 − 7.79 (m, 12H). **^{13}C NMR** (150MHz, $CDCl_3$) δ 32.5, 42.9, 105.0,

116.9, 118.9, 121.8, 124.1, 125.7, 126.6, 127.6, 128.0, 128.4, 129.0, 129.5, 132.1, 133.6, 134.0, 135.0, 146.0, 151.4, 161.2. **MS (m/z) =** 51, 77, 152, 165, 181, 194, 296 (M^+). Anal. Calcd. for $C_{21}H_{15}NO$: C, 84.82; H, 5.08; N, 4.71. Found: C, 84.75; H, 5.15; N, 4.59.

2-Fluoro-9-methyl-9-phenyl-9H-xanthene **(5f):**

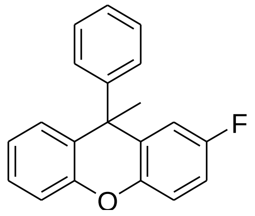

White solid (40% yield); mp: 89.5-90.5 °C. **IR** (cm^{-1}) ν = 3054, 3028, 2961, 2921, 1598, 1471, 1448, 1259, 750. **^{1}H NMR** (500 MHz, $CDCl_3$) δ 1.94 (s, 3H), 6.56 (dd, J_1 = 3.0 Hz, J_2 = 9.5 Hz, 1H), 6.85 (dd, J_1 = 1.5 Hz, J_2 = 8.0 Hz, 1H), 6.89 − 7.35 (m, 10H). **^{13}C NMR** (150MHz, $CDCl_3$) δ 31.9, 43.3, 114.4 (d, $^2J_{CF}$= 23.8 Hz), 114.7, 114.9 (d, $^2J_{CF}$= 23.9 Hz), 116.1, 117.3 (d, $^3J_{CF}$= 8.4 Hz), 118.2, 120.5 (d, $^3J_{CF}$= 8.4 Hz), 123.2, 126.5, 127.6, 128.1, 128.8, 129.2, 129.7, 148.2, 150.2, 157.5-158.4 (d, $^1J_{CF}$= 240.1 Hz). **MS (m/z) =** 213, 275, 290 (M^+). Anal. Calcd. for $C_{20}H_{15}FO$: C, 82.74; H, 5.21. Found: C, 82.68; H, 5.28.

9-Methyl-9-p-tolyl-9H-xanthene **(5g):**

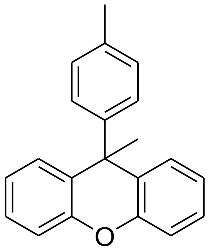

White solid (97% yield); mp: 81.6-82.4 °C.**IR** (cm^{-1}) ν = 3018, 2958, 2918, 1568, 1468, 1441, 1298, 1239, 746. **^{1}H NMR** (500 MHz, $CDCl_3$) δ 1.94 (s, 3H), 2.36 (s, 3H), 6.89 (dd, J_1 = 1.5 Hz, J_2 = 8.0 Hz, 2H), 6.99 (ddd, J_1 = 1.5 Hz, J_2 = J_3= 7.5 Hz, 2H), 7.05 − 7.28 (m, 8H). **^{13}C NMR** (150MHz, $CDCl_3$) δ 20.9, 32.2, 42.5, 116.1, 118.9, 123.2, 127.3, 128.5, 128.9, 129.7, 130.3, 135.7, 146.1, 150.2, 157.2. **MS (m/z) =** 195, 255, 271, 286 (M^+). Anal. Calcd. for $C_{21}H_{18}O$: C, 88.08; H, 6.34. Found: C, 88.13; H, 6.41.

9-(4-Methoxy-phenyl)-9-methyl-9H-xanthene **(5h):**

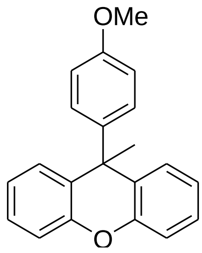

Pale yellow solid (83% yield); mp: 108.5-109.4°C. **IR** (cm^{-1}) ν = 3068, 3018, 2955, 2918, 1594, 1438, 1239, 1022, 750. **^{1}H NMR** (500 MHz, $CDCl_3$) δ 1.91 (s, 3H), 3.81 (s, 3H), 6.84 (d, J = 8.5 Hz, 1H), 6.87 (dd, J_1 = 1.5 Hz, J_2 = 8.0 Hz, 2H), 6.95 (ddd, J_1 = 1.5 Hz, J_2 = J_3= 7.5 Hz, 2H), 7.10 (dd, J_1 = 1.5 Hz, J_2 = 8.5 Hz, 2H), 7.19 (ddd, J_1 = 1.5 Hz, J_2 = J_3= 8.0 Hz, 2H), 7.25 (d, J = 8.5 Hz, 2H), 7. 28 (s, 1H). **^{13}C NMR** (150MHz, $CDCl_3$) δ 32.4, 42.3, 55.2, 113.2, 116.1, 123.0, 127.3, 128.9, 129.6,130.4, 141.2, 150.2, 157.7. **MS (m/z) =** 152, 165, 195, 243, 287, 302 (M^+). Anal. Calcd. for $C_{21}H_{18}O_2$: C, 83.42; H, 6.00. Found: C, 83.51; H, 6.08.

9-Methyl-9-naphthalen-1-yl-9H-xanthene **(5i):**

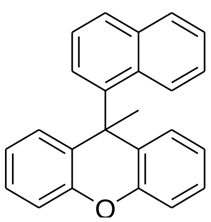

White solid (76% yield); mp: 134.0-136.2 °C. **IR** (cm^{-1}) ν = 3044, 2965, 1594, 1568, 1475, 1438, 1298, 1242, 743. . **^{1}H NMR** (400 MHz, $CDCl_3$) δ 1.98 (s, 3H), 7.14 − 7.27 (m, 5H), 7.60 − 7.64 (d, *J* = 7.5 Hz, 2H),, 7.80 − 7.82 (d, *J* = 7.2 Hz, 2H), 7.89 − 7.91 (d, *J* = 7.0 Hz, 2H), 7.99 − 8.01 (d, *J* = 7.2 Hz, 2H),. **^{13}C NMR** (150 MHz, $CDCl_3$) δ 35.6, 43.2, 116.1, 123.1, 123.3, 124.4, 124.8, 125.0, 126.1, 127.4, 127.5, 128.7, 128.9, 129.1, 135.2, 140.9, 149.8. **MS** (m/z) = 153, 165, 195, 307, 322 (M^+). Anal. Calcd. for $C_{24}H_{18}O$: C, 89.41; H, 5.63. Found: C, 89.54; H, 5.73.

9-Cyclopentyl-9-methyl-9H-xanthene **(5j):**

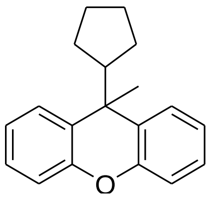

Yellow oily (20% yield). **IR** (cm–1) ν = 3061, 3034, 2958, 2918, 1688, 1574, 1475, 1451, 1222, 1009, 796, 750. **^{1}H NMR** (500 MHz, $CDCl_3$) δ 1.33 − 1.39 (m, 2H), 1.56 − 1.66 (m, 6H), 1.67 (s, 3H), 2.54 (q, *J* = 7.5 Hz, 1H), 6.91 (dd, J_1 = 1.0 Hz, J_2 = 8.0 Hz, 1H), 7.08 (dd, J_1 = 1.5 Hz, J_2 = 8.0 Hz, 2H), 7.21 (dd, J_1 = J_2 = 7.5 Hz, 2H), 7.41 (dd, J_1 = 7.5 Hz, J_2 = 8.5 Hz, 2H), 7.53 (ddd, J_1 = 2.0 Hz, J_2 = J_3= 8.0 Hz, 1H). **^{13}C NMR** (150MHz, $CDCl_3$) δ 29.3, 29.7, 30.2, 31.9, 53.4, 118.4, 119.4, 123.3, 124.3, 124.3, 128.4, 130.0, 135.7, 156.3, 159.9. **MS** (m/z) = 77, 104, 180, 189, 195, 249, 264 (M^+), 265, 266. Anal. Calcd. for $C_{19}H_{20}O$: C, 86.32; H, 7.63. Found: C, 86.48; H, 7.76.

9-Methyl-9-thiophen-2-yl-9H-xanthene **(5k):**

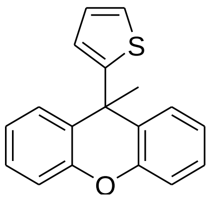

Pale brown solid (99% yield); mp: 134.0-136.2 °C. **IR** (cm^{-1}) ν = 3064, 3024, 2958, 2915, 1598, 1468, 1431, 1235, 750. **^{1}H NMR** (500 MHz, $CDCl_3$) δ 2.02 (s, 3H), 6.97 (dd, J_1 = 4.0 Hz, J_2 = 5.0 Hz, 1H), 7.01 − 7.05 (m, 3H), 7.13 (ddd, J_1 =1.5 Hz, J_2 = J_3= 8.0 Hz, 4H), 7.22 − 7.28 (m, 3H). **^{13}C NMR** (150MHz, $CDCl_3$) δ 33.7, 40.9, 116.3, 118.8, 123.1, 125.4, 125.9 127.9, 128.4, 129.2, 149.7, 154.9. **MS** (m/z) = 83, 92, 110, 173, 180, 195, 264, 278 (M^+). Anal. Calcd. for $C_{18}H_{14}OS$: C, 77.66; H, 5.07; S, 11.52. Found: C, 77.71; H, 5.12; S, 11.41.

2-Bromo-9-methyl-9-phenyl-9H-xanthene **(5l):**

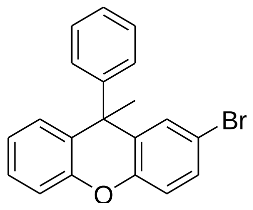

Colorless oily (93% yield). **IR** (cm^{-1}) ν = 3066, 3025, 2987, 2933, 1594, 1463, 1451, 1278, 758. **^{1}H NMR** (500 MHz, $CDCl_3$) δ 1.93 (s, 3H), 6.89 − 6.91 (m, 3H), 7.01 − 7.03 (d, *J* = 8.5 Hz, 2H), 7.13 − 7.16 (m, 1H), 7.34 − 7.45 (m, 6H). **^{13}C NMR** (150MHz, $CDCl_3$) δ 29.7, 43.0, 110.3, 115.5, 119.02, 119.1, 119.9, 120.4, 123.5, 123.7, 129.8, 132.6,

132.8, 136.1, 147.7, 153.3, 156.5, 156.7. **MS (m/z) =** 78, 152, 168, 181, 257, 271, 335, 350 (M^+). Anal. Calcd. for $C_{20}H_{15}BrO$: C, 68.39; H, 4.30. Found: C, 68.45; H, 4.24.

References

1. Yeager, G. W.; Schissel, D. N. *Synthesis* **1995**, *1*, 28.
2. Lambelet, P.; Lucken, E. A. C. *J. Chem. Soc., Perkin Trans.* **1975**, *2*, 1652.
3. Wang, M.; Force, G; Carpentier, J.P.; Sarazin, Y.; Bour, C.; Gandon**,** V.; Lebœuf, D. *Org. Lett.* **2021**, 23, *7*, 2565.

NMR Spectra of all the reported compounds:

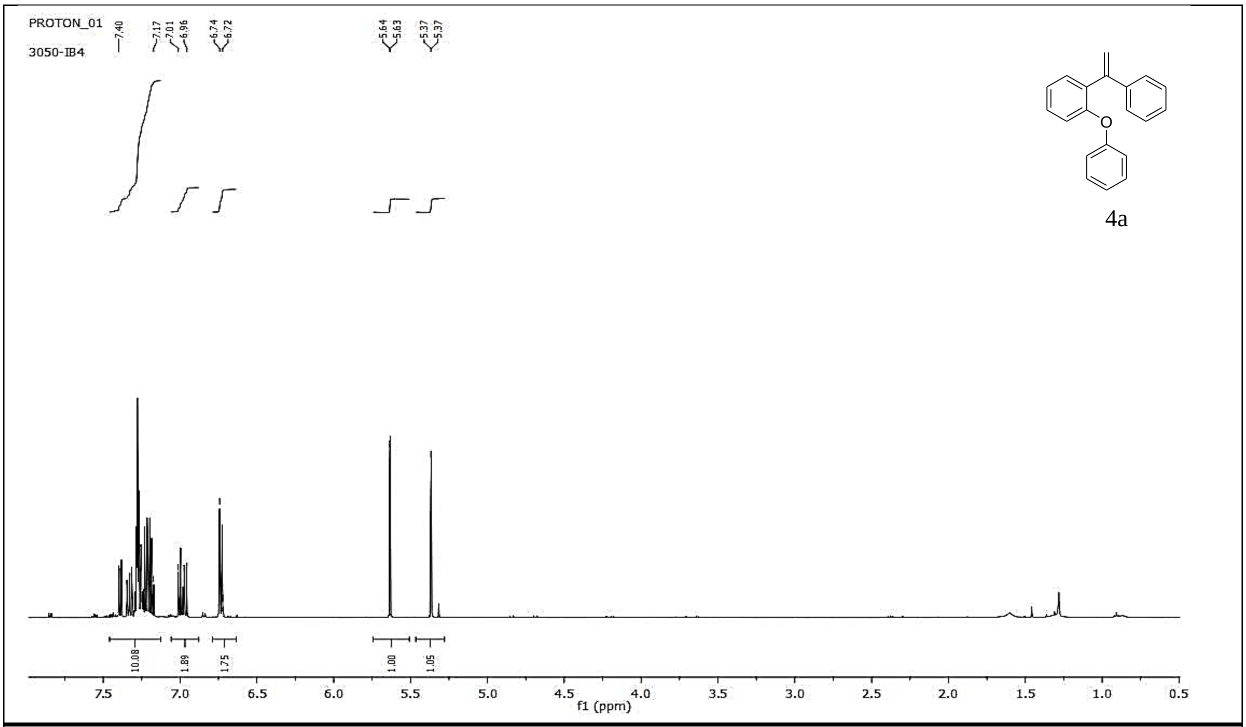

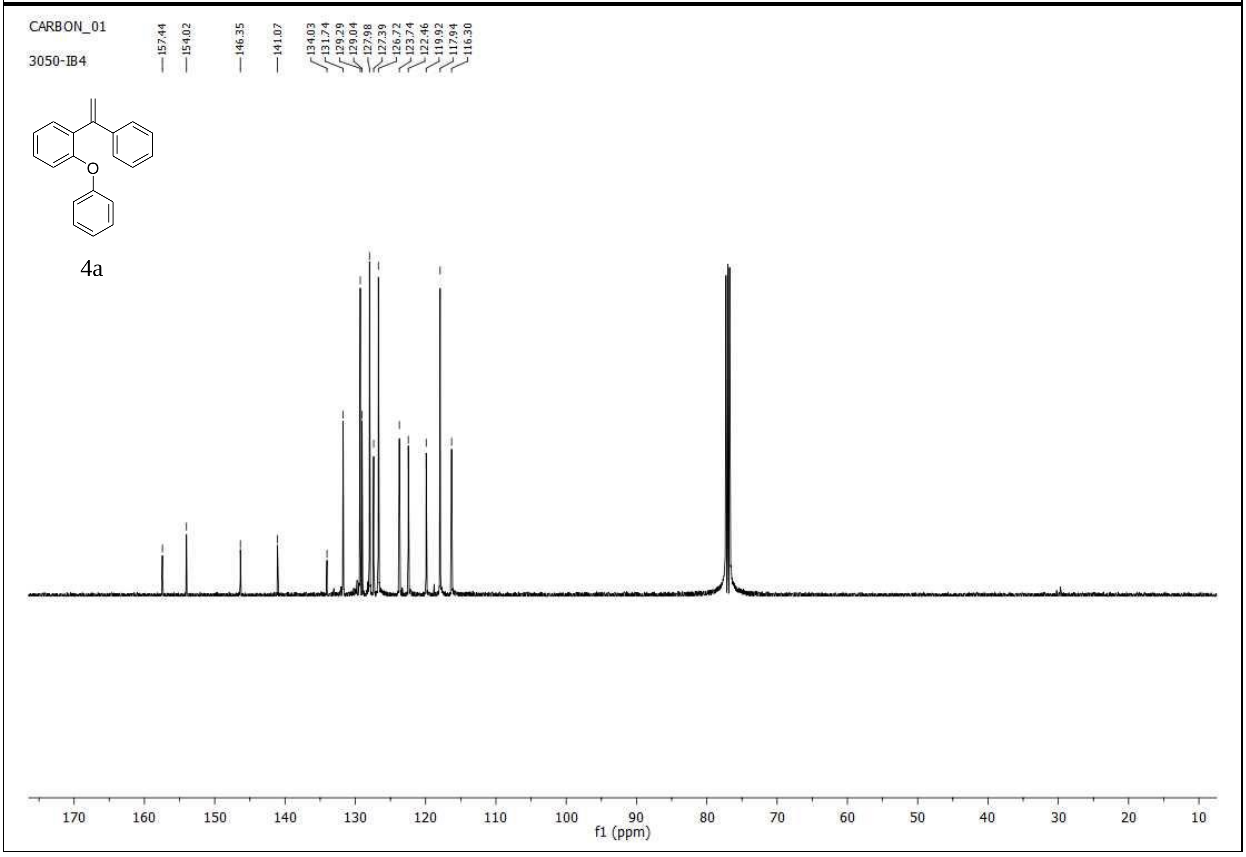

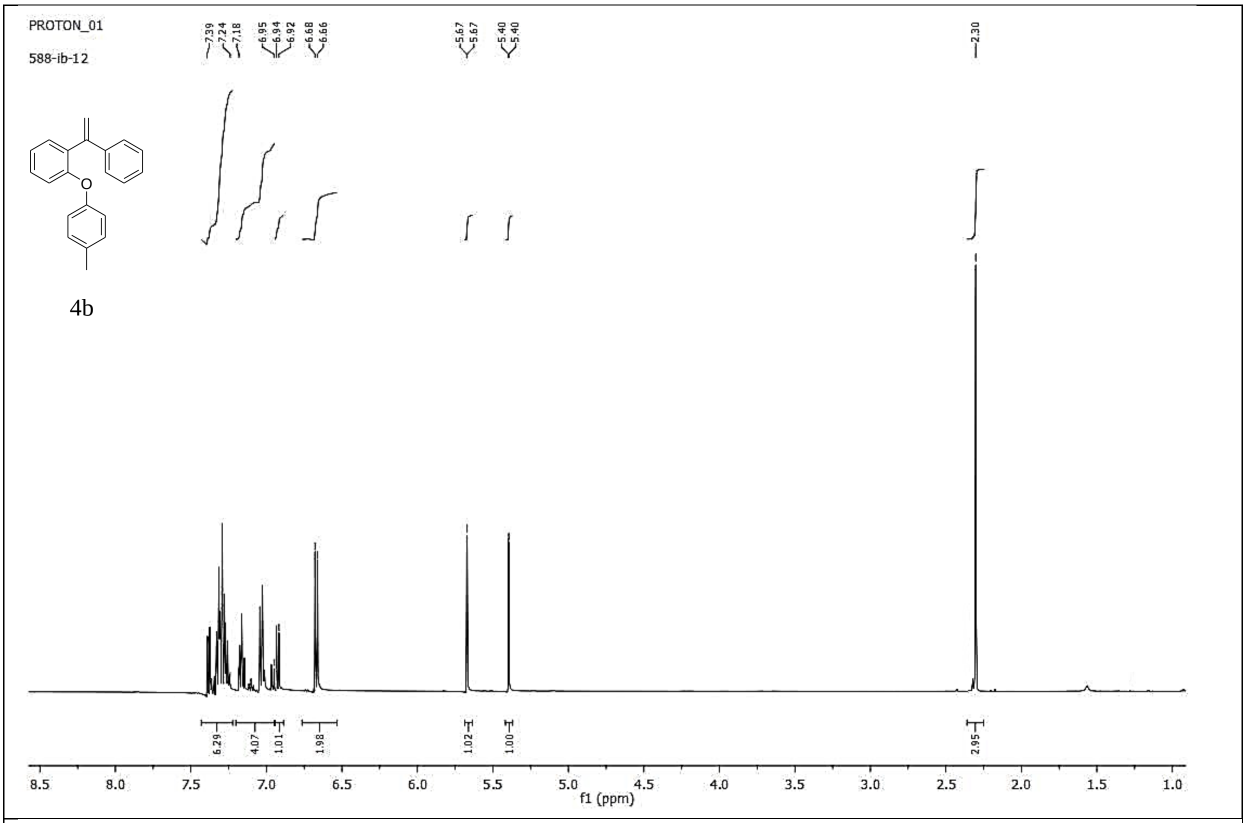
PROTON_01
588-ib-12
7.39
7.24
7.18
6.95
6.94
6.92
6.68
6.66
5.67
5.67
5.40
5.40
2.30
O
4b
6.29
4.07
1.01
1.98
1.02
1.00
2.95
8.5 8.0 7.5 7.0 6.5 6.0 5.5 5.0 4.5 4.0 3.5 3.0 2.5 2.0 1.5 1.0
f1 (ppm)

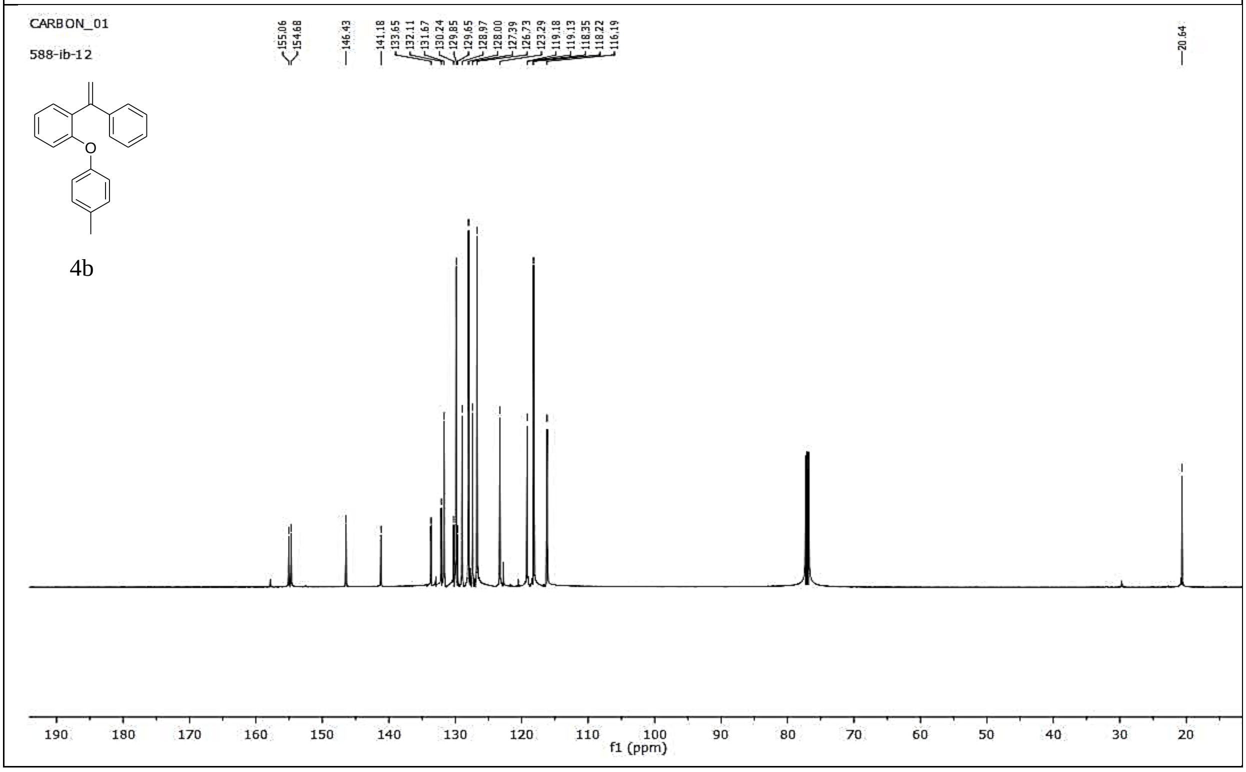
CARBON_01
588-ib-12
155.06
154.68
146.43
141.18
133.65
132.11
131.67
130.24
129.85
129.65
128.97
128.00
127.39
126.73
123.29
119.18
119.13
118.35
118.22
116.19
20.64
O
4b
190 180 170 160 150 140 130 120 110 100 90 80 70 60 50 40 30 20
f1 (ppm)

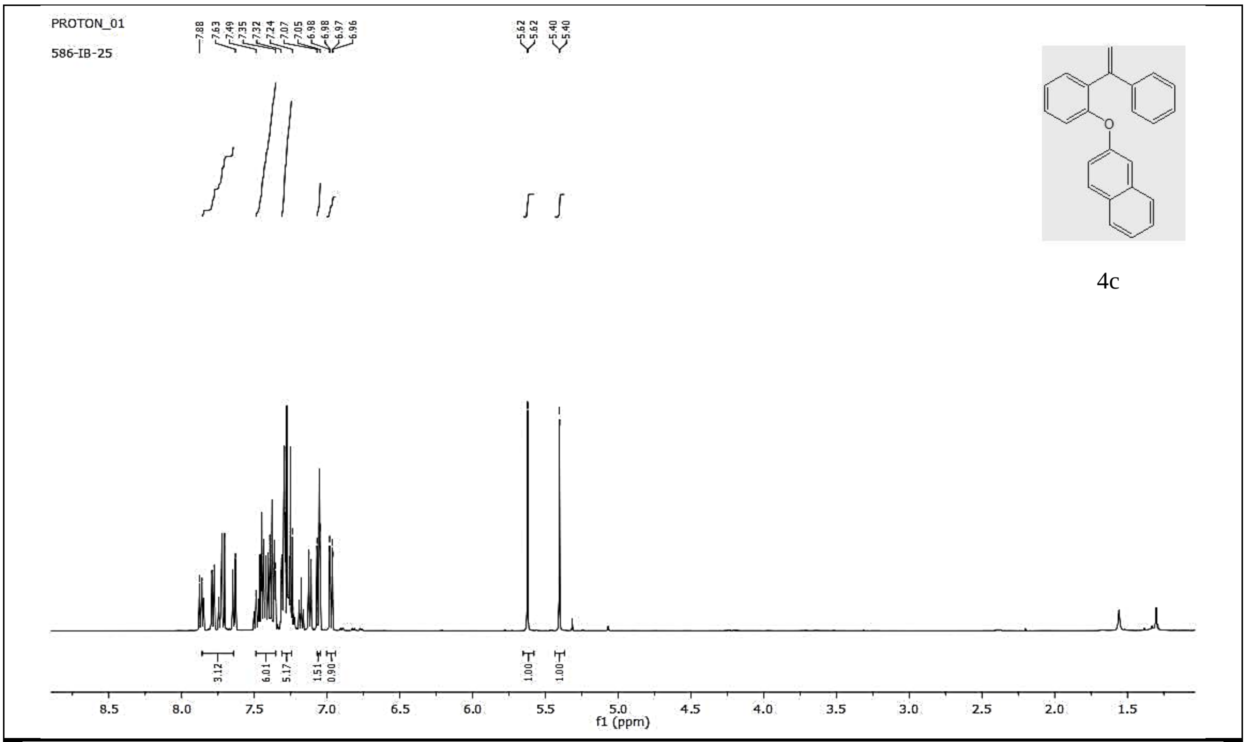
PROTON_01
586-IB-25
7.88
7.63
7.49
7.35
7.32
7.24
7.07
7.05
6.98
6.98
6.97
6.96
5.62
5.62
5.40
5.40
O
4c
3.12
6.01
5.17
1.51
0.90
1.00
1.00
8.5 8.0 7.5 7.0 6.5 6.0 5.5 5.0 4.5 4.0 3.5 3.0 2.5 2.0 1.5
f1 (ppm)

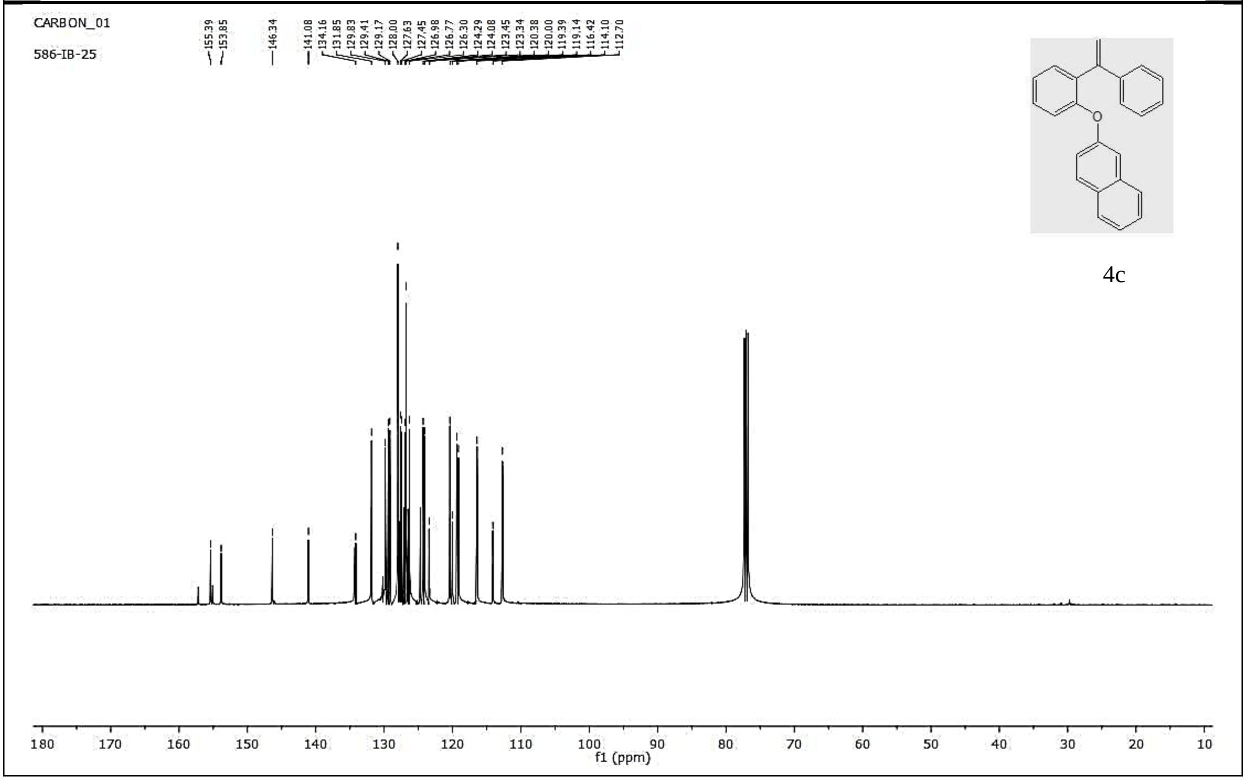
CARBON_01
586-IB-25
155.39
153.85
146.34
141.08
134.16
131.85
129.83
129.41
129.17
128.00
127.63
127.45
126.98
126.77
126.30
124.29
124.08
123.45
123.34
120.38
120.00
119.39
119.14
116.42
114.10
112.70
O
4c
180 170 160 150 140 130 120 110 100 90 80 70 60 50 40 30 20 10
f1 (ppm)

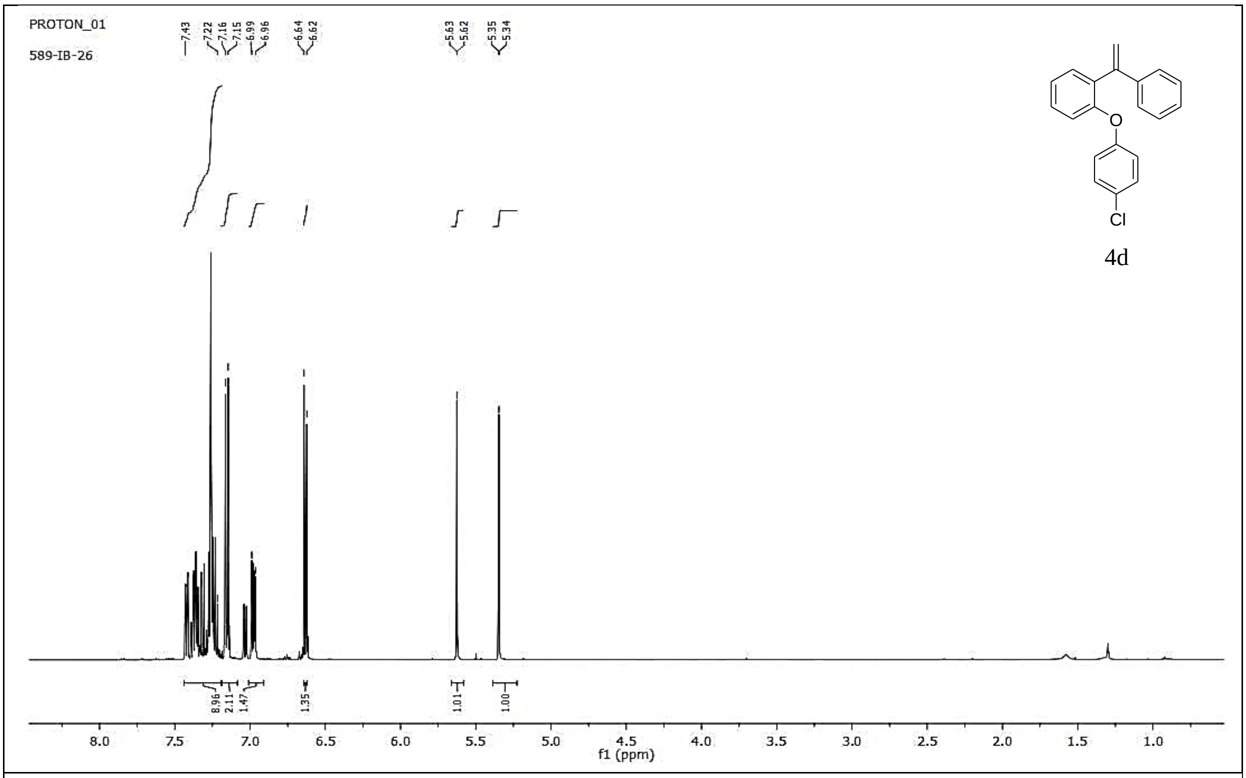
PROTON_01
589-IB-26
7.43
7.22
7.16
7.15
6.99
6.96
6.64
6.62
5.63
5.62
5.35
5.34
8.96
2.11
1.47
1.35
1.01
1.00
O
Cl
4d
8.0 7.5 7.0 6.5 6.0 5.5 5.0 4.5 4.0 3.5 3.0 2.5 2.0 1.5 1.0
f1 (ppm)

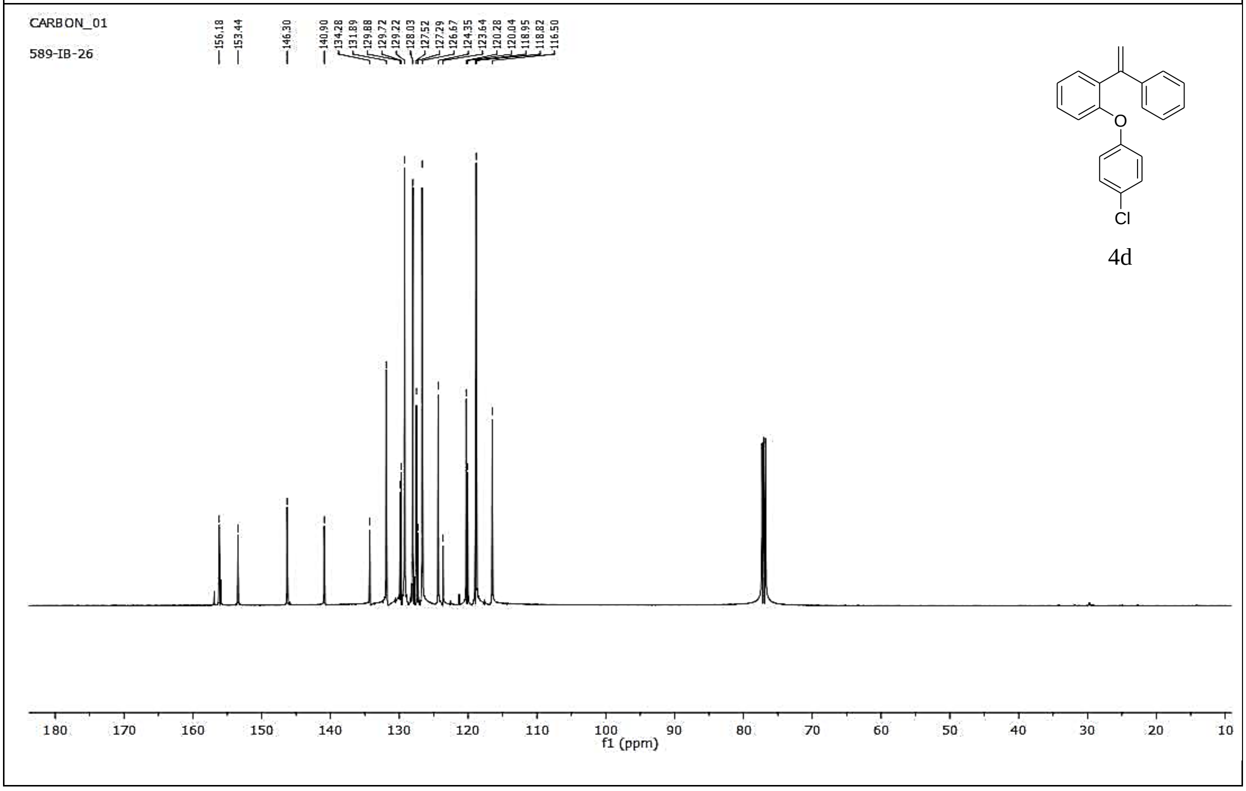
CARBON_01
589-IB-26
156.18
153.44
146.30
140.90
134.28
131.89
129.88
129.72
129.22
128.03
127.52
127.29
126.67
124.35
123.64
120.28
120.04
118.95
118.82
116.50
O
Cl
4d
180 170 160 150 140 130 120 110 100 90 80 70 60 50 40 30 20 10
f1 (ppm)

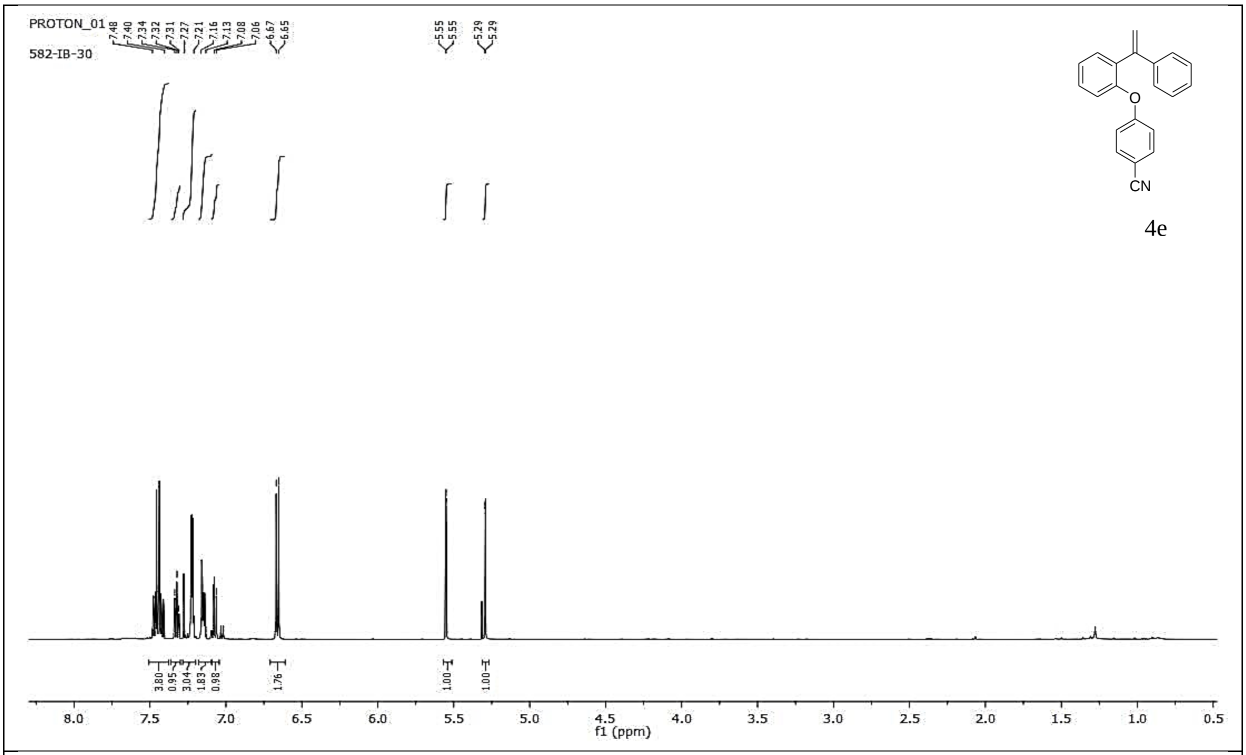
PROTON_01
582-IB-30
7.48
7.40
7.34
7.32
7.31
7.27
7.21
7.16
7.13
7.08
7.06
6.67
6.65
5.55
5.55
5.29
5.29
3.80
0.95
3.04
1.83
0.98
1.76
1.00
1.00
O
CN
4e
8.0
7.5
7.0
6.5
6.0
5.5
5.0
4.5
4.0
3.5
3.0
2.5
2.0
1.5
1.0
0.5
f1 (ppm)

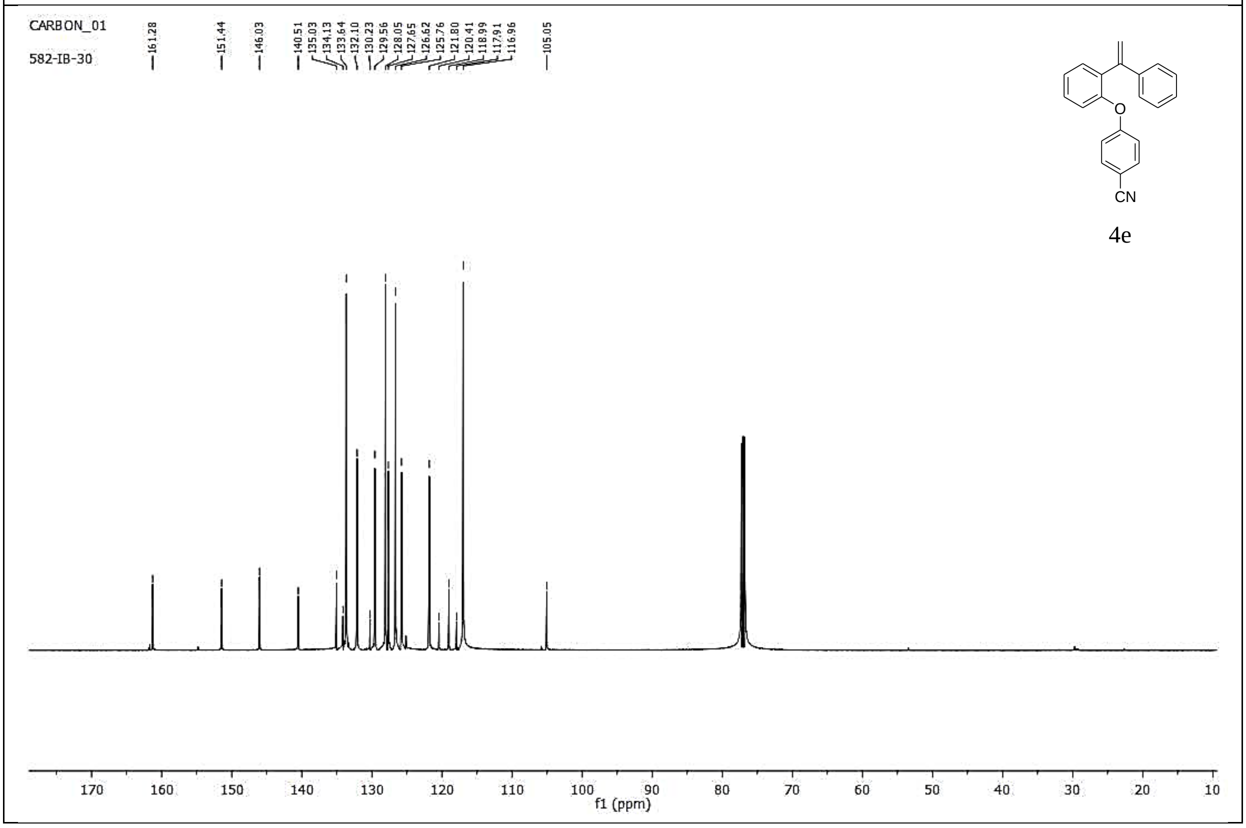
CARBON_01
582-IB-30
161.28
151.44
146.03
140.51
135.03
134.13
133.64
132.10
130.23
129.56
128.05
127.65
126.62
125.76
121.80
120.41
118.99
117.91
116.96
105.05
O
CN
4e
170
160
150
140
130
120
110
100
90
80
70
60
50
40
30
20
10
f1 (ppm)

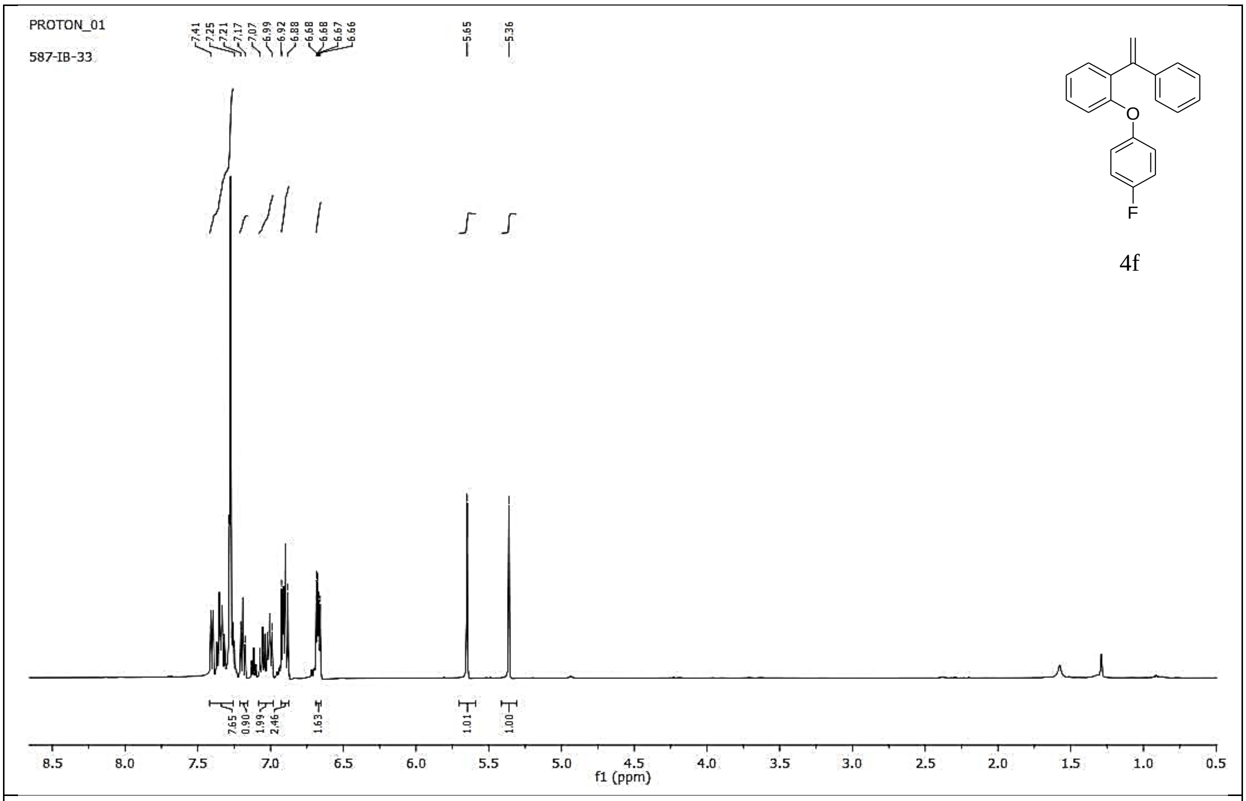
PROTON_01
587-IB-33
7.41 7.25 7.21 7.17 7.07 6.99 6.92 6.88 6.68 6.68 6.67 6.66
5.65
5.36
7.65 0.90 1.99 2.46 1.63 1.01 1.00
O
F
4f
8.5 8.0 7.5 7.0 6.5 6.0 5.5 5.0 4.5 4.0 3.5 3.0 2.5 2.0 1.5 1.0 0.5
f1 (ppm)

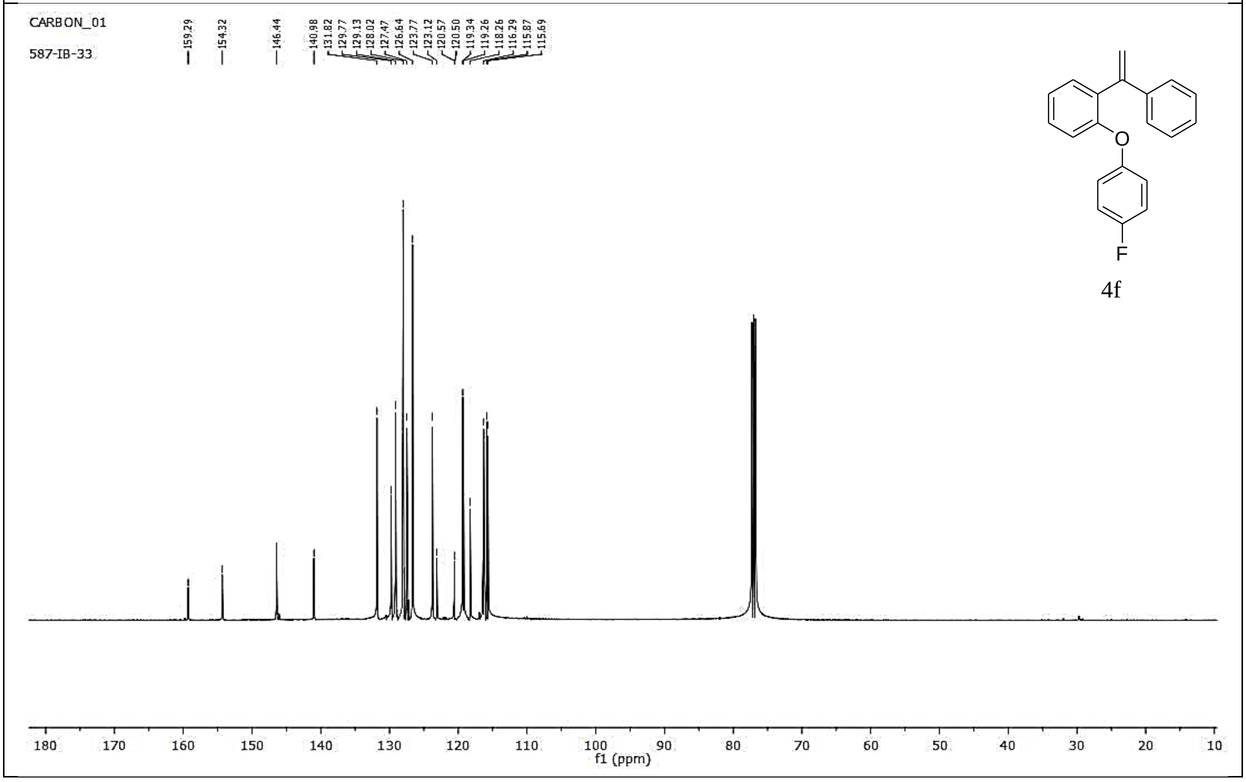
CARBON_01
587-IB-33
159.29
154.32
146.44
140.98 131.82 129.77 129.13 128.02 127.47 126.64 123.77 123.12 120.57 120.50 119.34 119.26 118.26 116.29 115.87 115.69
O
F
4f
180 170 160 150 140 130 120 110 100 90 80 70 60 50 40 30 20 10
f1 (ppm)

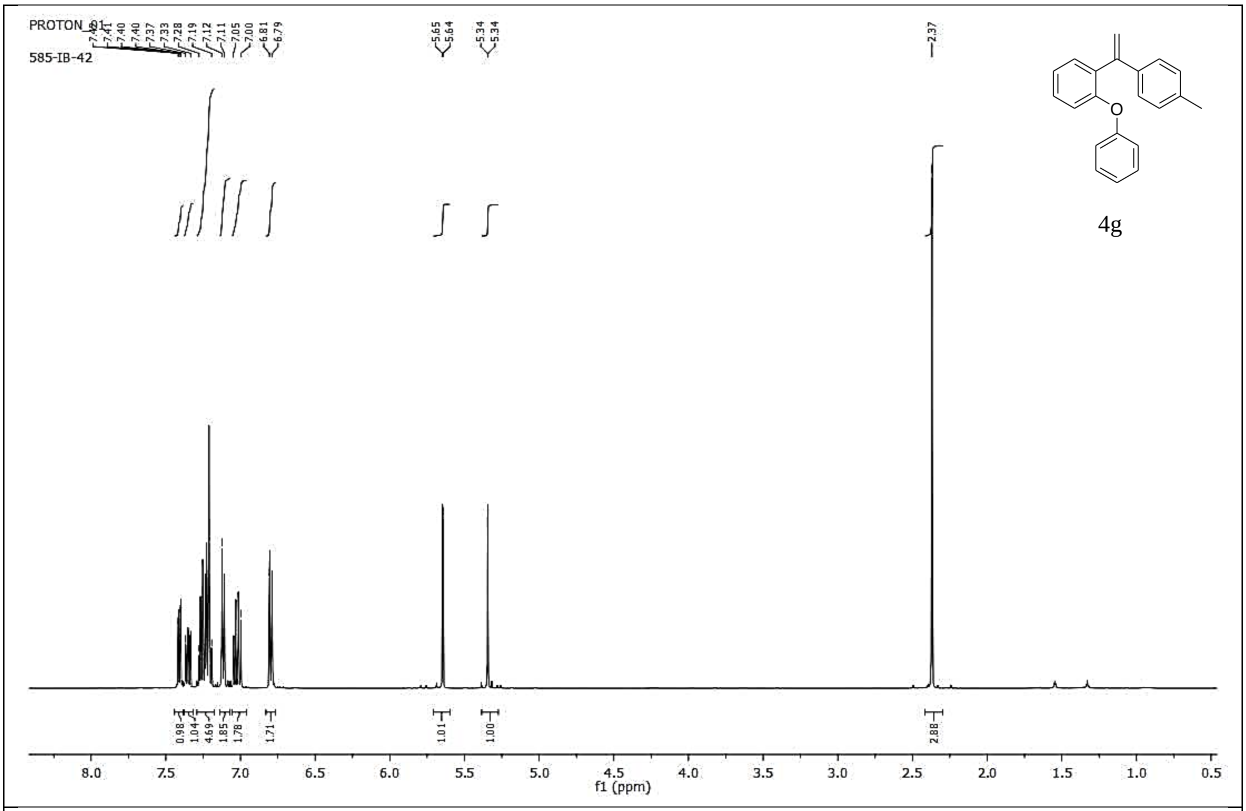
PROTON_01
585-IB-42
7.41 7.41 7.40 7.40 7.37 7.33 7.28 7.19 7.12 7.11 7.05 7.00 6.81 6.79
5.65 5.64
5.34 5.34
2.37
O
4g
0.98 1.04 4.69 1.85 1.78 1.71
1.01
1.00
2.88
8.0 7.5 7.0 6.5 6.0 5.5 5.0 4.5 4.0 3.5 3.0 2.5 2.0 1.5 1.0 0.5
f1 (ppm)

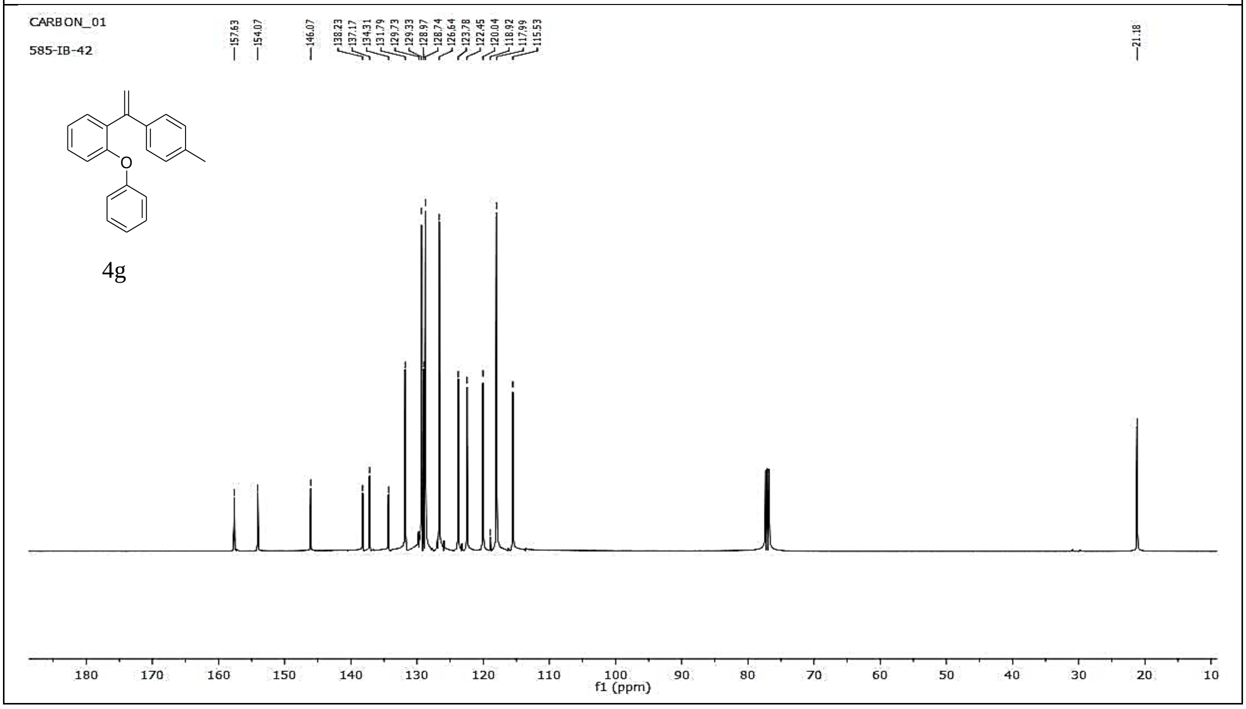
CARBON_01
585-IB-42
157.63
154.07
146.07
138.23 137.17 134.31 131.79 129.73 129.33 128.97 128.74 126.64 123.78 122.45 120.04 118.92 117.99 115.53
21.18
O
4g
180 170 160 150 140 130 120 110 100 90 80 70 60 50 40 30 20 10
f1 (ppm)

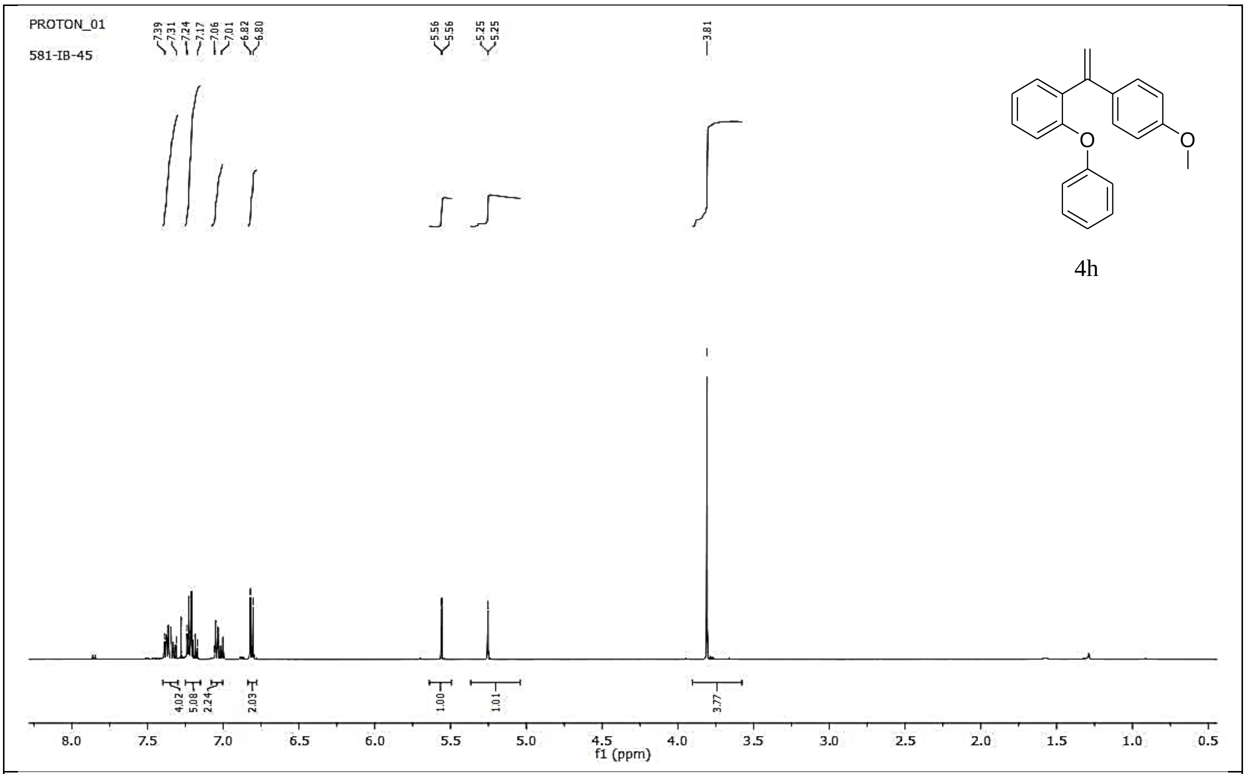
PROTON_01
581-IB-45
7.39
7.31
7.24
7.17
7.06
7.01
6.82
6.80
5.56
5.56
5.25
5.25
3.81
4h
4.02
5.08
2.24
2.03
1.00
1.01
3.77
f1 (ppm)

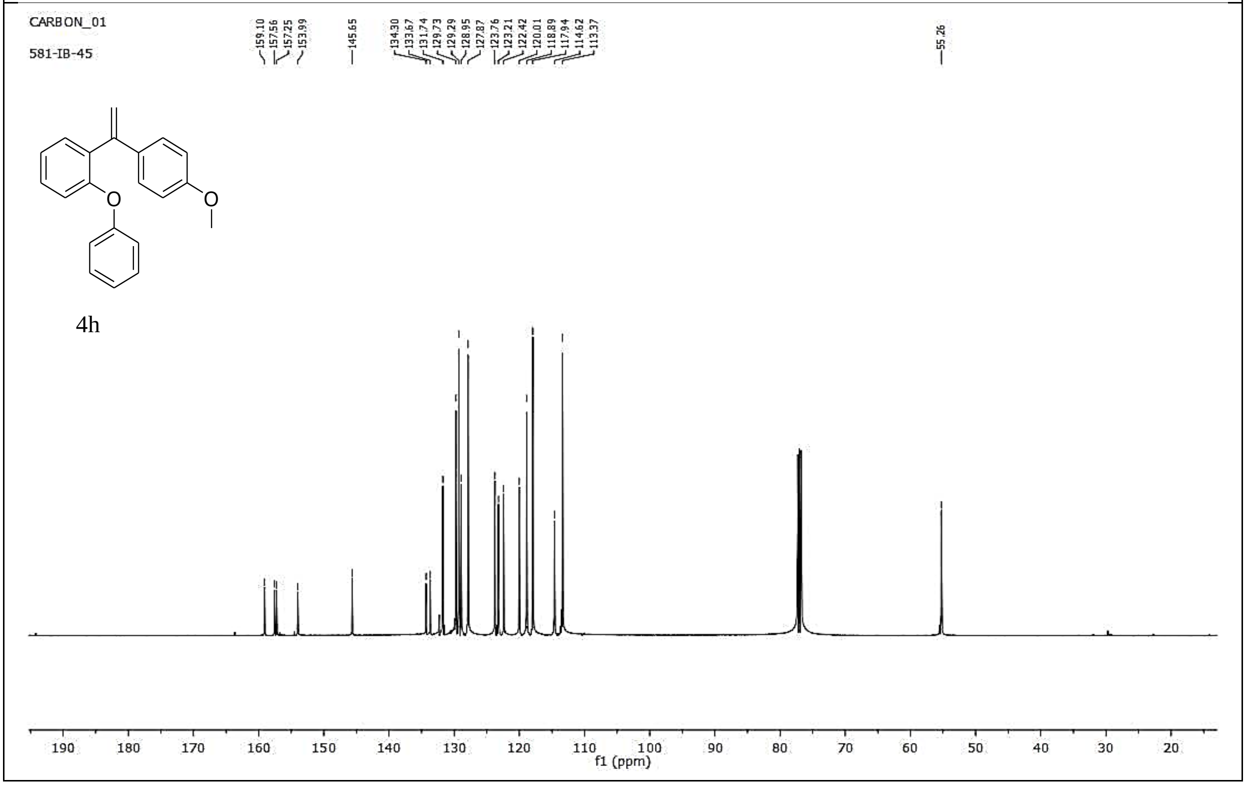
CARBON_01
581-IB-45
159.10
157.56
157.25
153.99
145.65
134.30
133.67
131.74
129.73
129.29
128.95
127.87
123.76
123.21
122.42
120.01
118.89
117.94
114.62
113.37
55.26
4h
f1 (ppm)

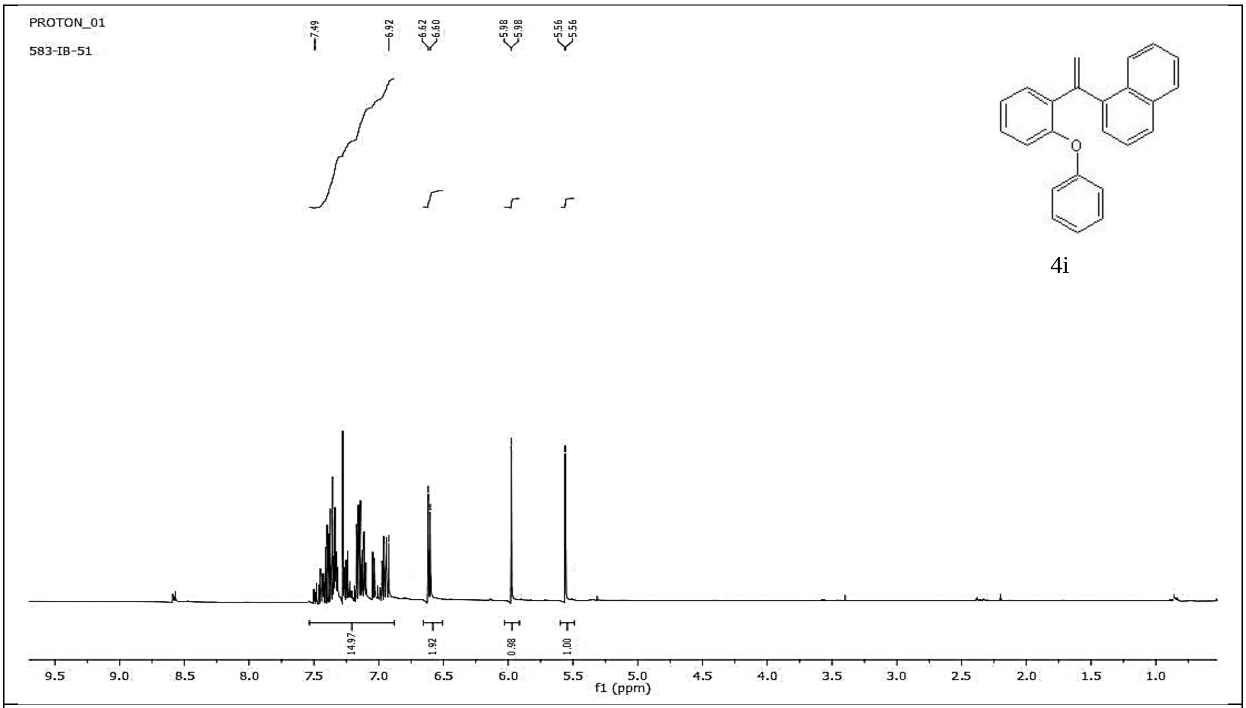
PROTON_01
583-IB-51
4i
14.97
1.92
0.98
1.00
f1 (ppm)

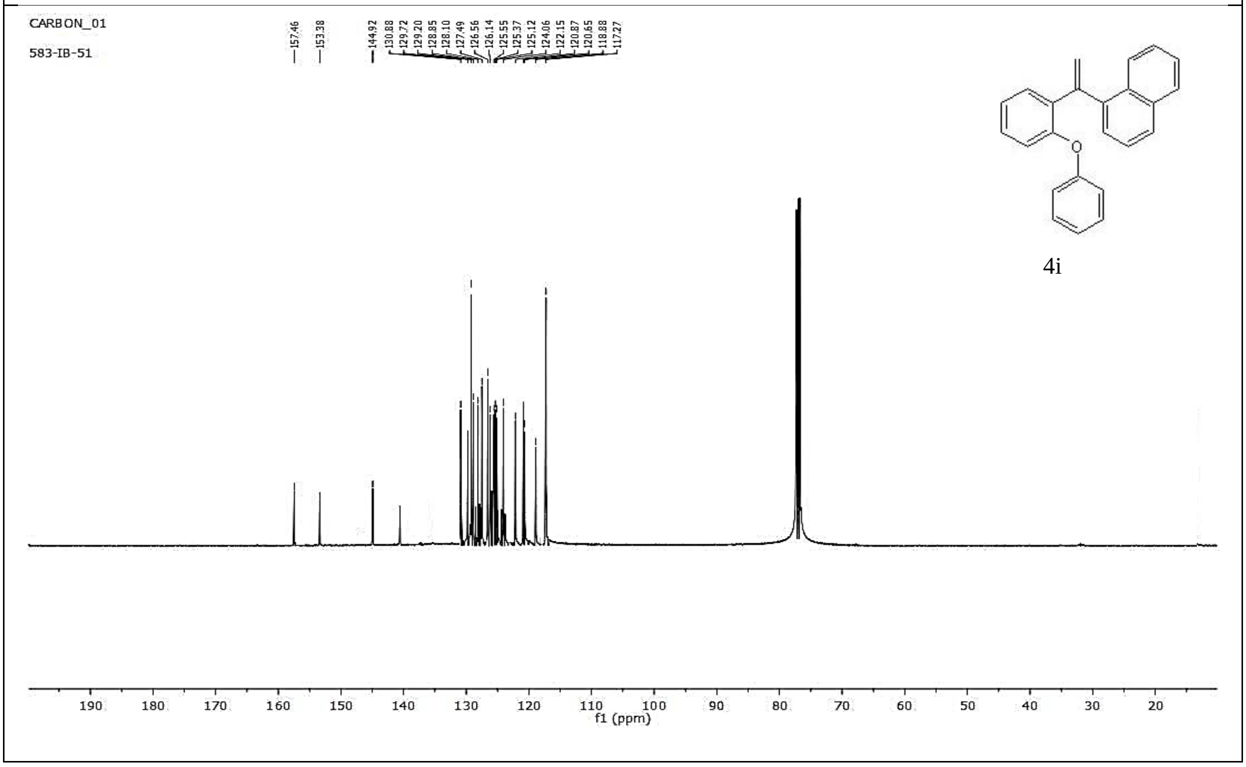
CARBON_01
583-IB-51
4i
f1 (ppm)

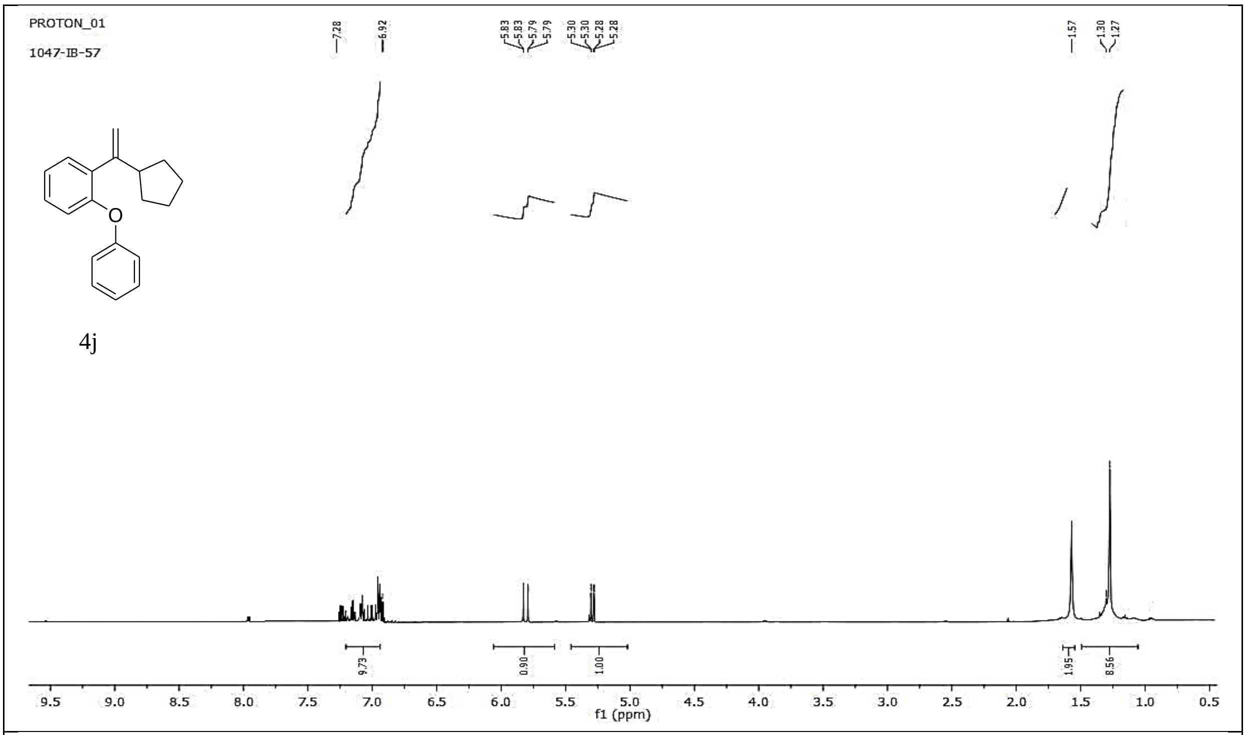
PROTON_01
1047-IB-57
4j
7.28
6.92
5.83
5.83
5.79
5.79
5.30
5.30
5.28
5.28
1.57
1.30
1.27
9.73
0.90
1.00
1.95
8.56
f1 (ppm)

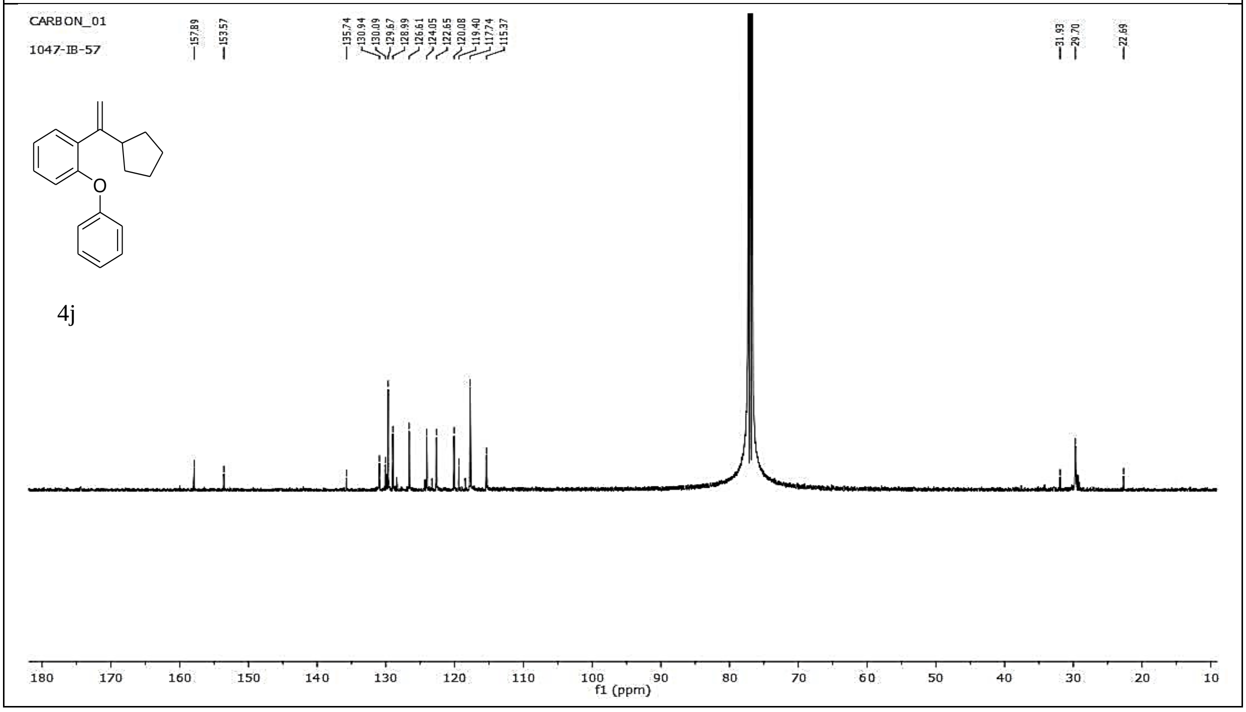
CARBON_01
1047-IB-57
4j
157.89
153.57
135.74
130.94
130.09
129.67
128.99
126.61
124.05
122.65
120.08
119.40
117.74
115.37
31.93
29.70
22.69
f1 (ppm)

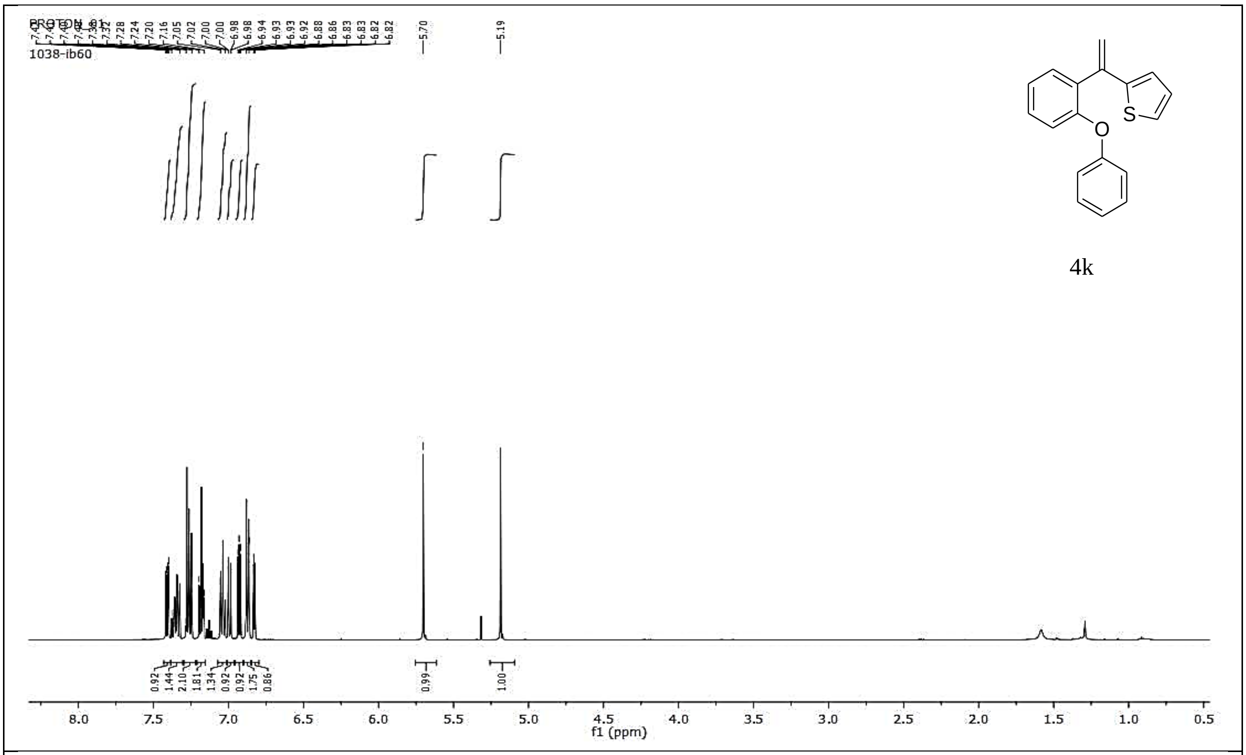
PROTON_01
1038-ib60
5.70
5.19
0.99
1.00
f1 (ppm)
4k

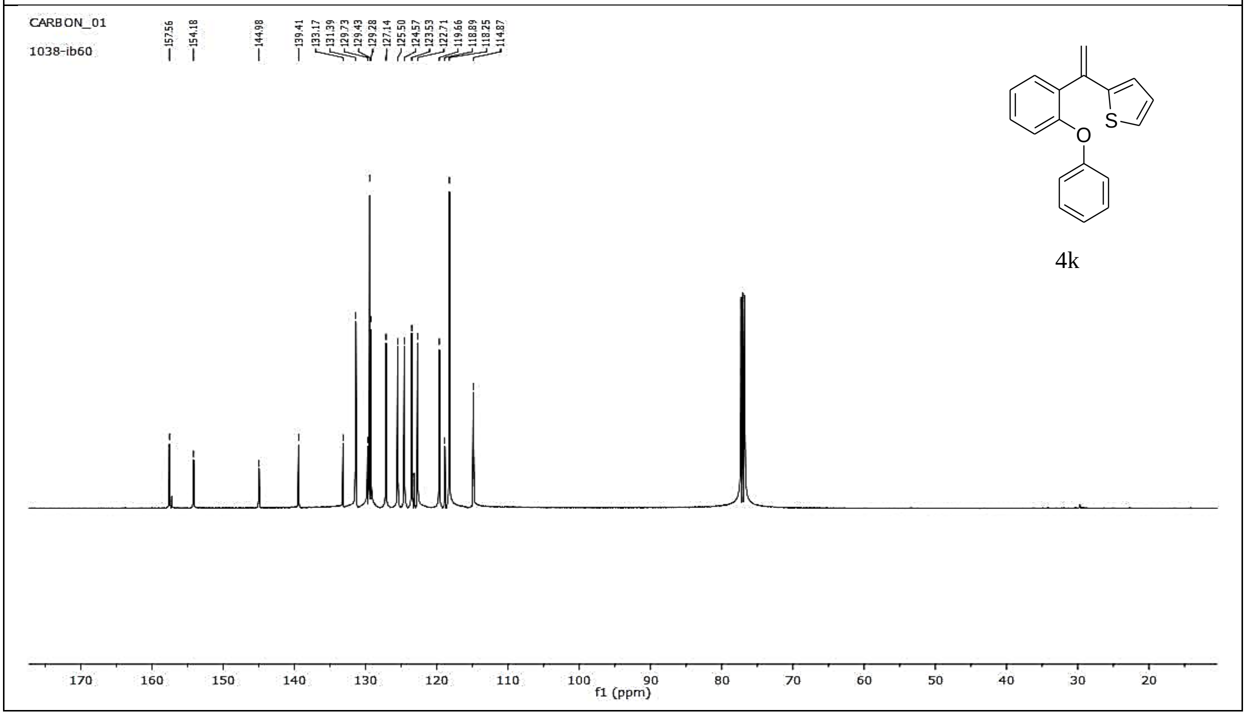
CARBON_01
1038-ib60
157.56
154.18
144.98
139.41
133.17
131.39
129.73
129.43
129.28
127.14
125.50
124.57
123.53
122.71
119.66
118.89
118.25
114.87
f1 (ppm)
4k

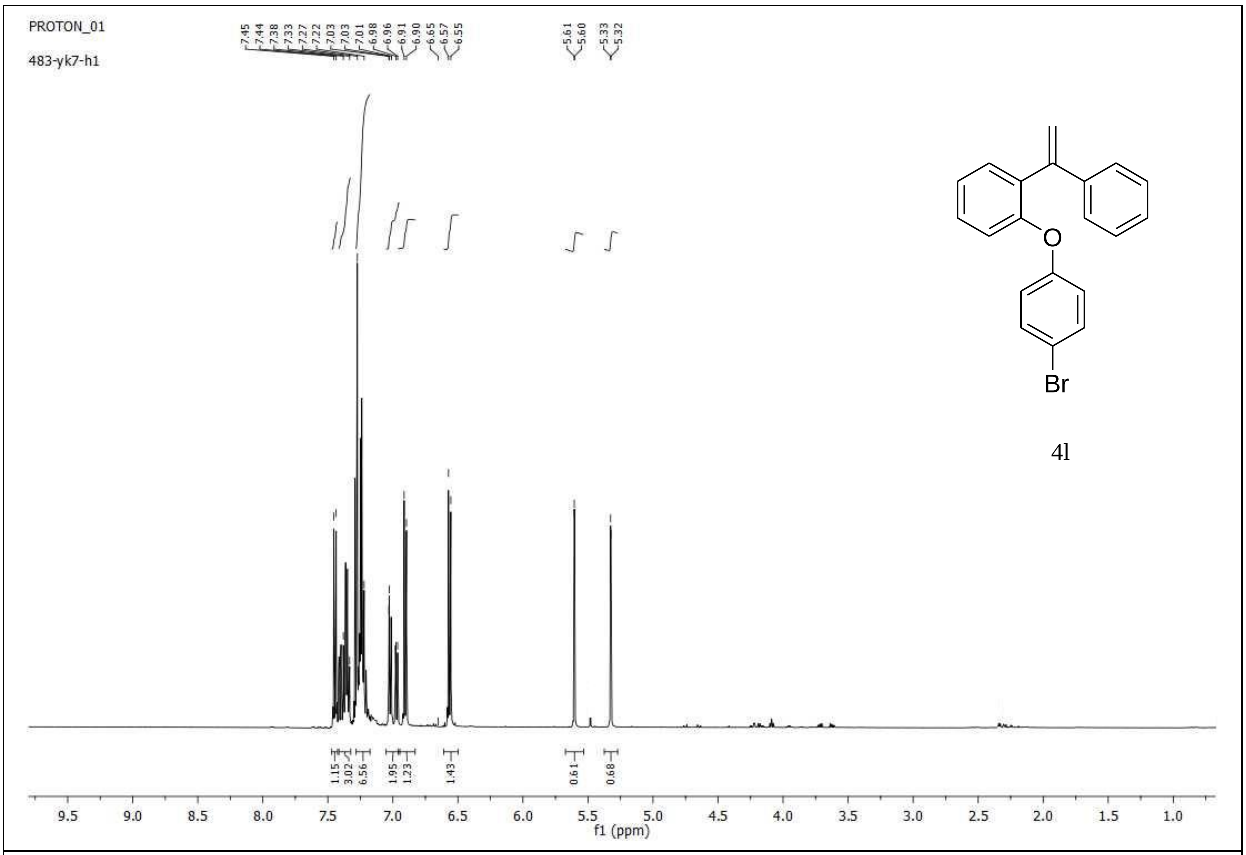
PROTON_01
483-yk7-h1
7.45 7.44 7.38 7.33 7.27 7.22 7.03 7.03 7.01 6.98 6.96 6.91 6.90 6.65 6.57 6.55
5.61 5.60 5.33 5.32
1.15 3.02 6.56 1.95 1.23 1.43 0.61 0.68
O
Br
4l
f1 (ppm)

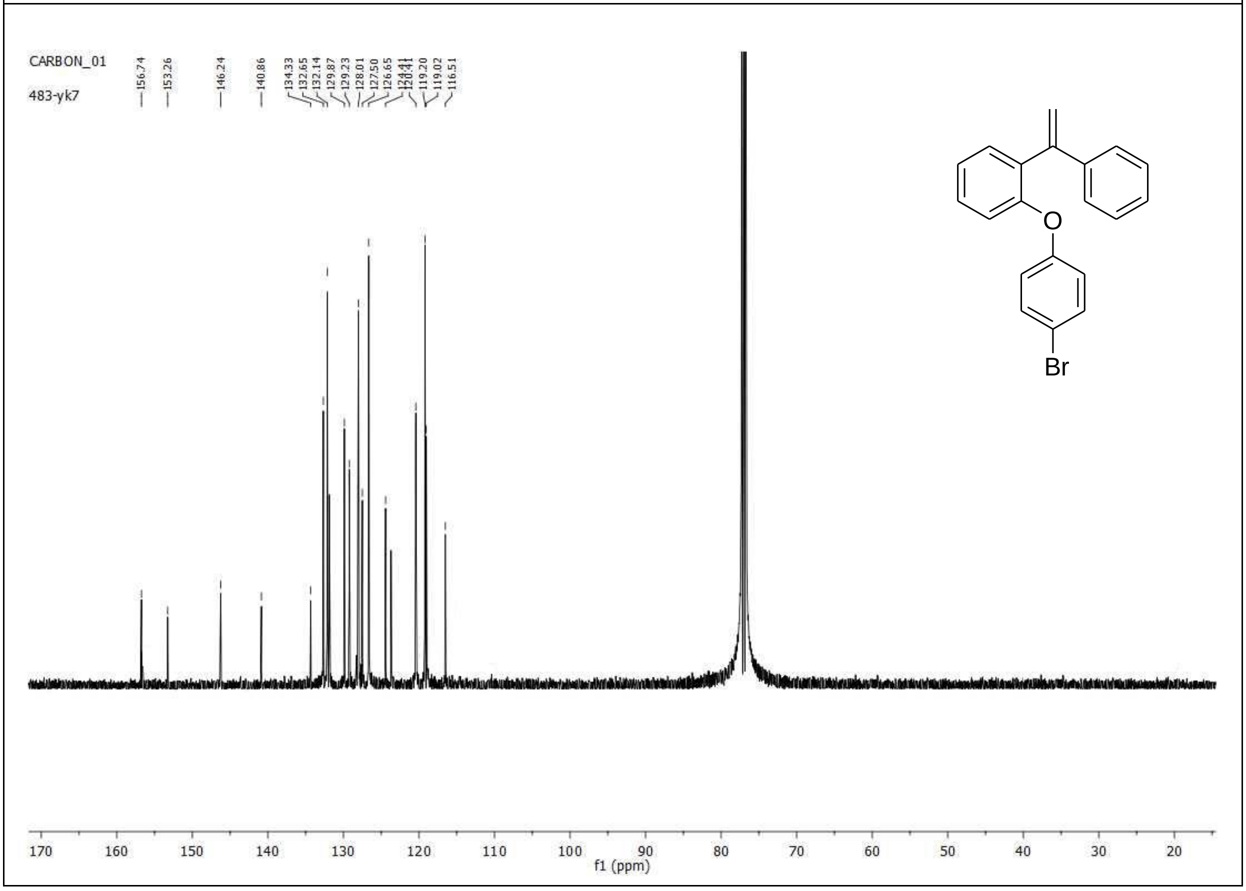
CARBON_01
483-yk7
156.74 153.26 146.24 140.86 134.33 132.65 132.14 129.87 129.23 128.01 127.50 126.65 119.20 119.02 116.51
O
Br
f1 (ppm)

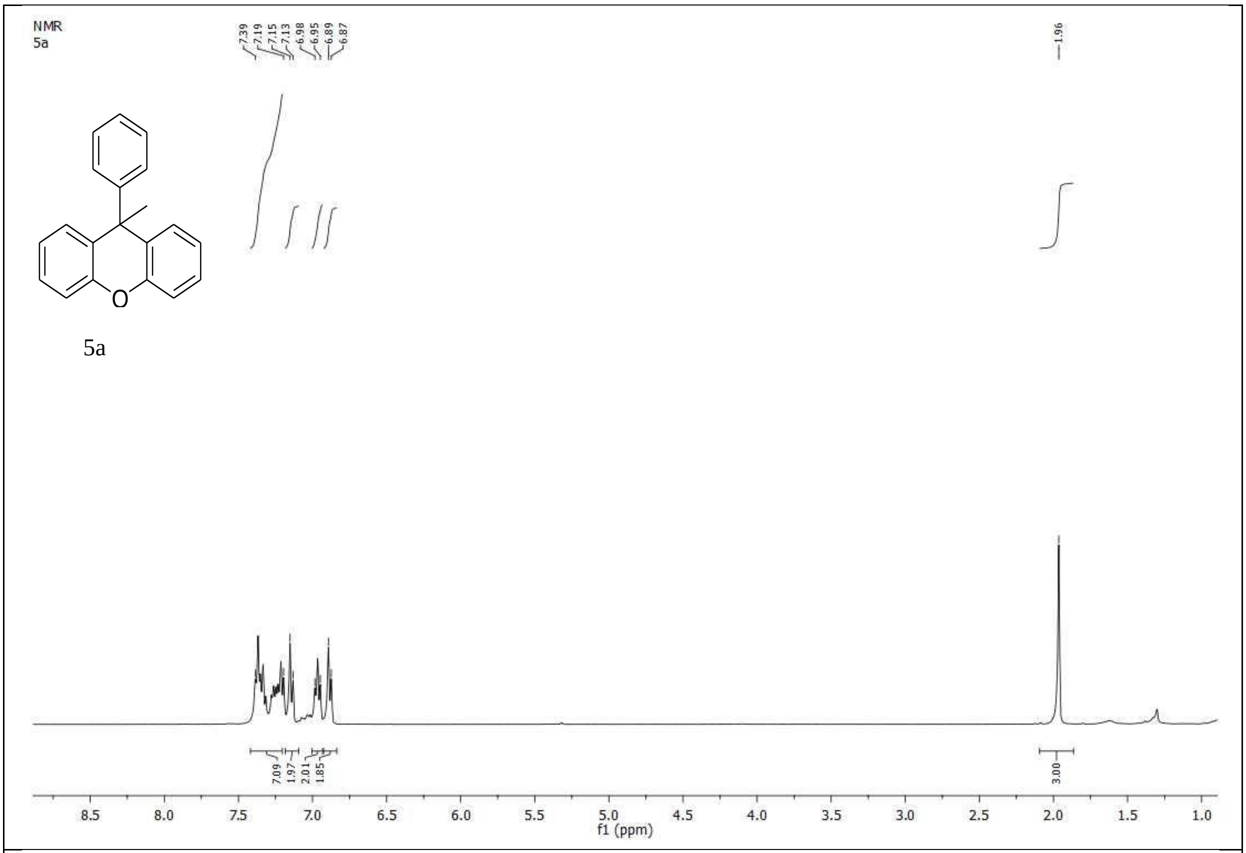
NMR
5a
7.39
7.19
7.15
7.13
6.98
6.95
6.89
6.87
1.96
O
5a
7.09
1.97
2.01
1.85
3.00
f1 (ppm)

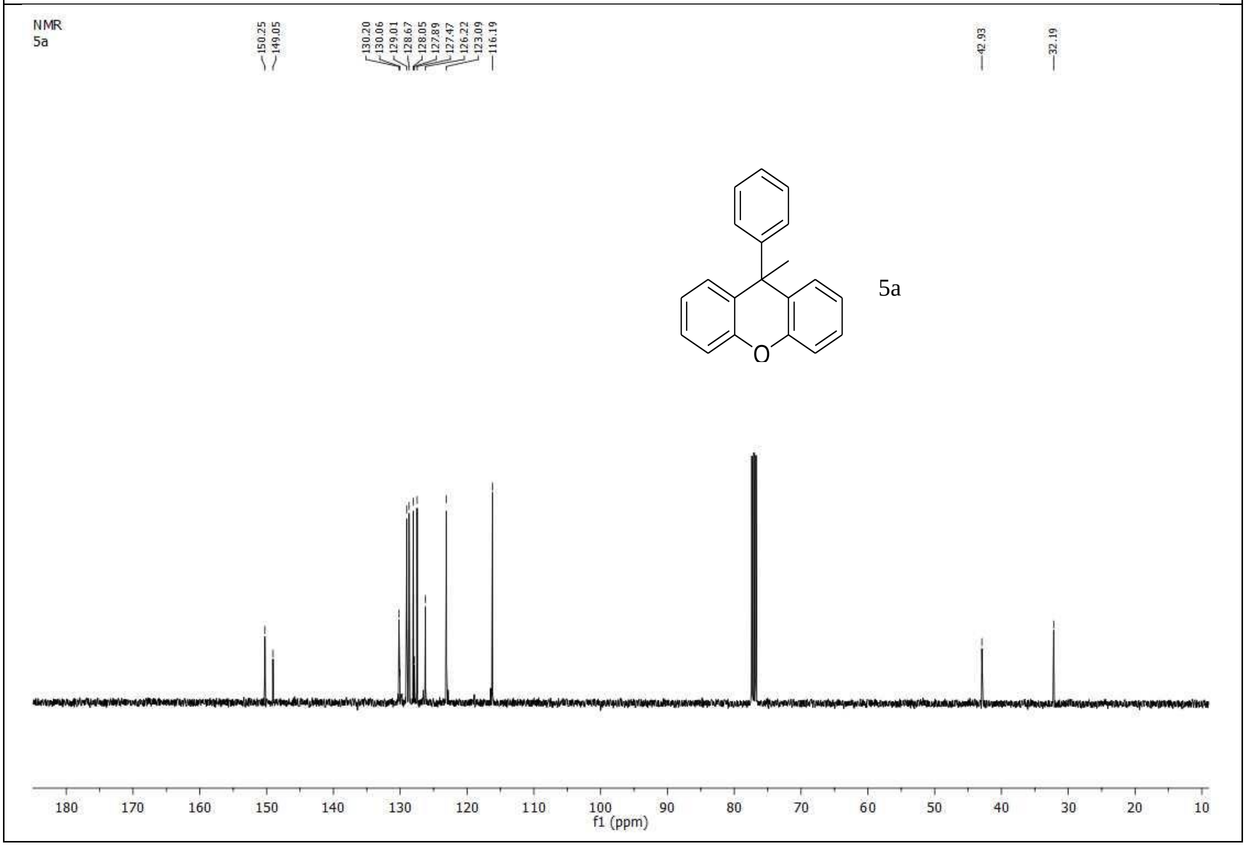
NMR
5a
150.25
149.05
130.20
130.06
129.01
128.67
128.05
127.89
127.47
126.22
123.09
116.19
42.93
32.19
O
5a
f1 (ppm)

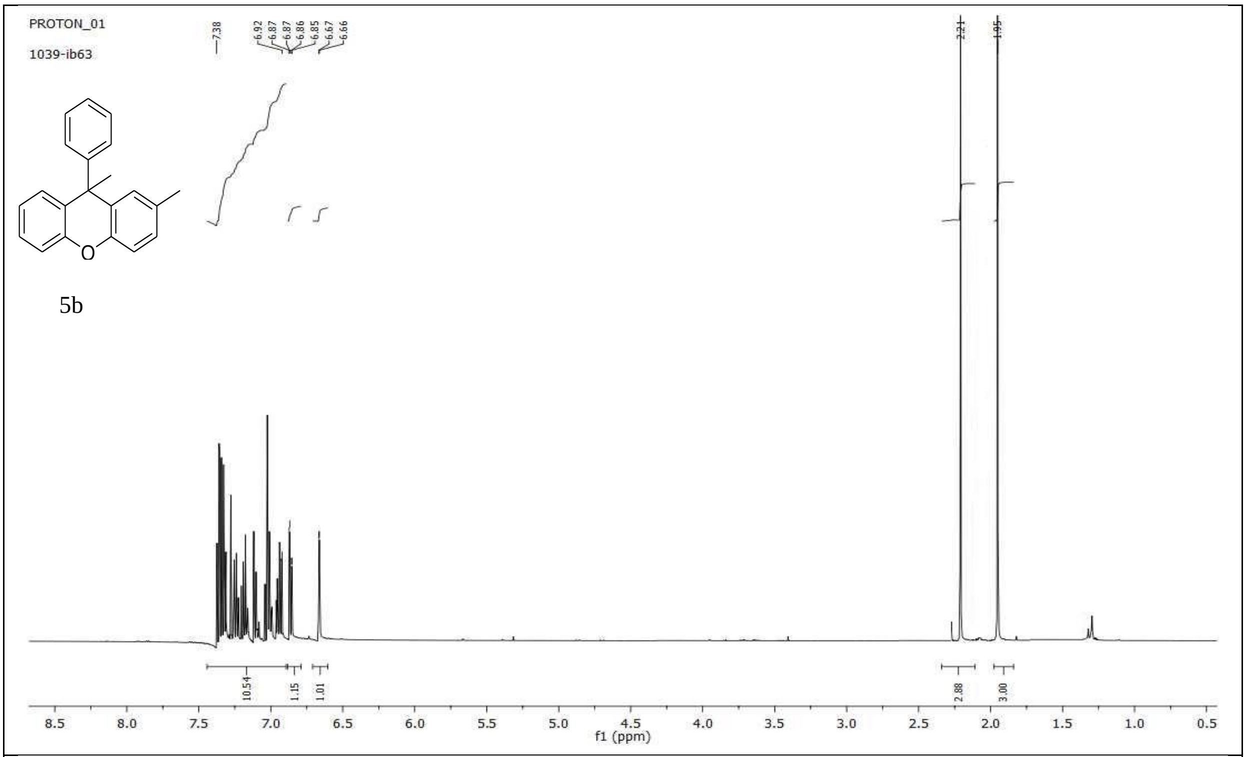
PROTON_01
1039-ib63
7.38
6.92
6.87
6.87
6.86
6.85
6.67
6.66
2.21
1.95
O
5b
10.54
1.15
1.01
2.88
3.00
8.5 8.0 7.5 7.0 6.5 6.0 5.5 5.0 4.5 4.0 3.5 3.0 2.5 2.0 1.5 1.0 0.5
f1 (ppm)

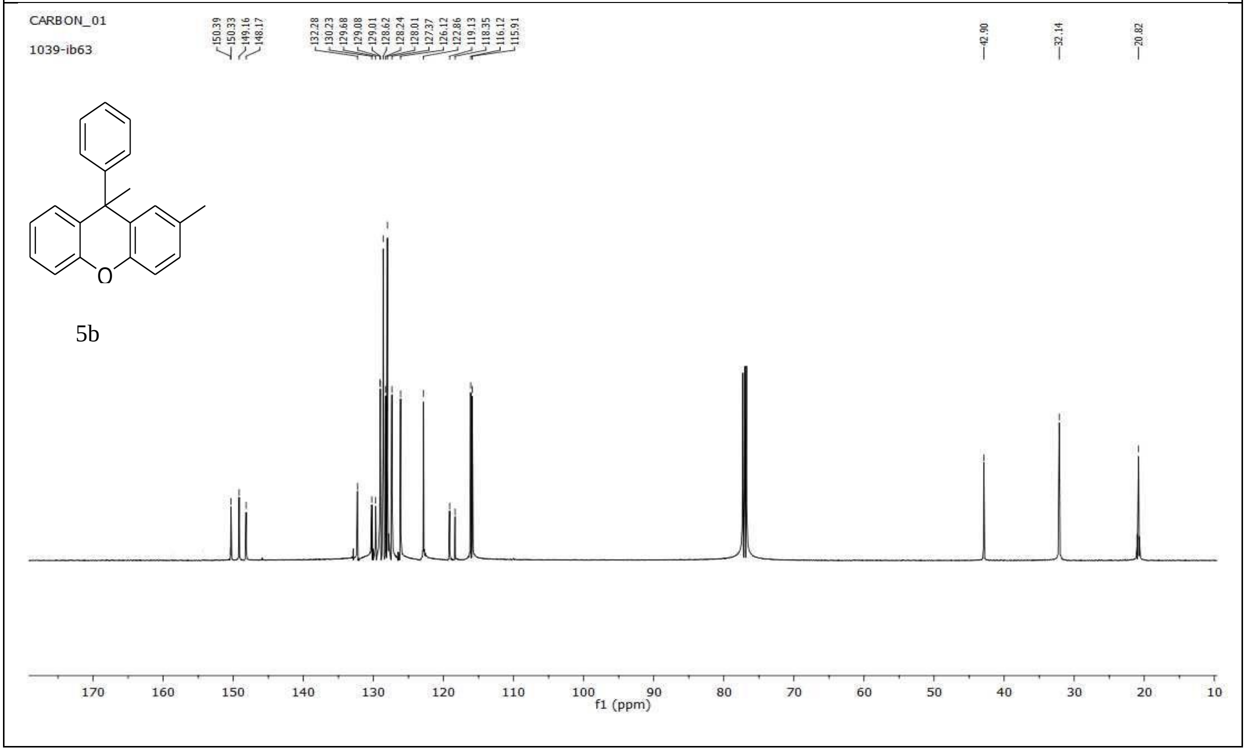
CARBON_01
1039-ib63
150.39
150.33
149.16
148.17
132.28
130.23
129.68
129.08
129.01
128.62
128.24
128.01
127.37
126.12
122.86
119.13
118.35
116.12
115.91
42.90
32.14
20.82
O
5b
170 160 150 140 130 120 110 100 90 80 70 60 50 40 30 20 10
f1 (ppm)

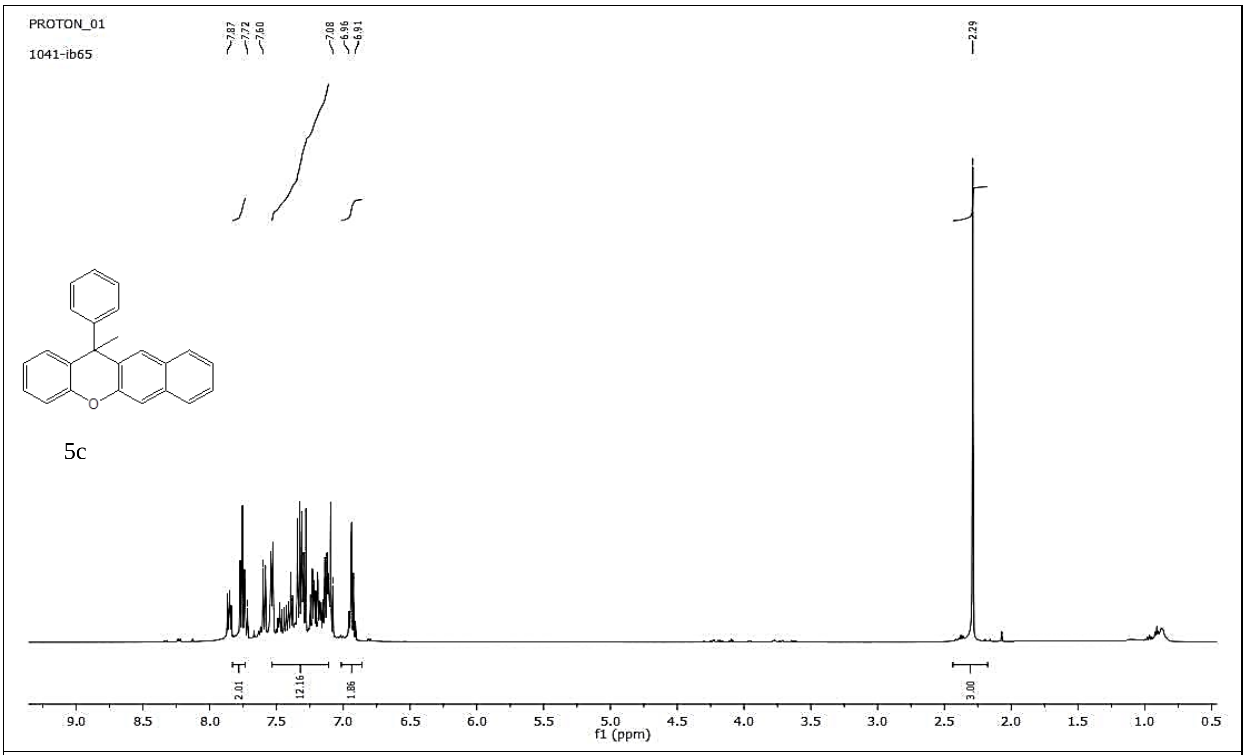
PROTON_01
1041-ib65
7.87
7.72
7.60
7.08
6.96
6.91
2.29
5c
2.01
12.16
1.86
3.00
9.0 8.5 8.0 7.5 7.0 6.5 6.0 5.5 5.0 4.5 4.0 3.5 3.0 2.5 2.0 1.5 1.0 0.5
f1 (ppm)

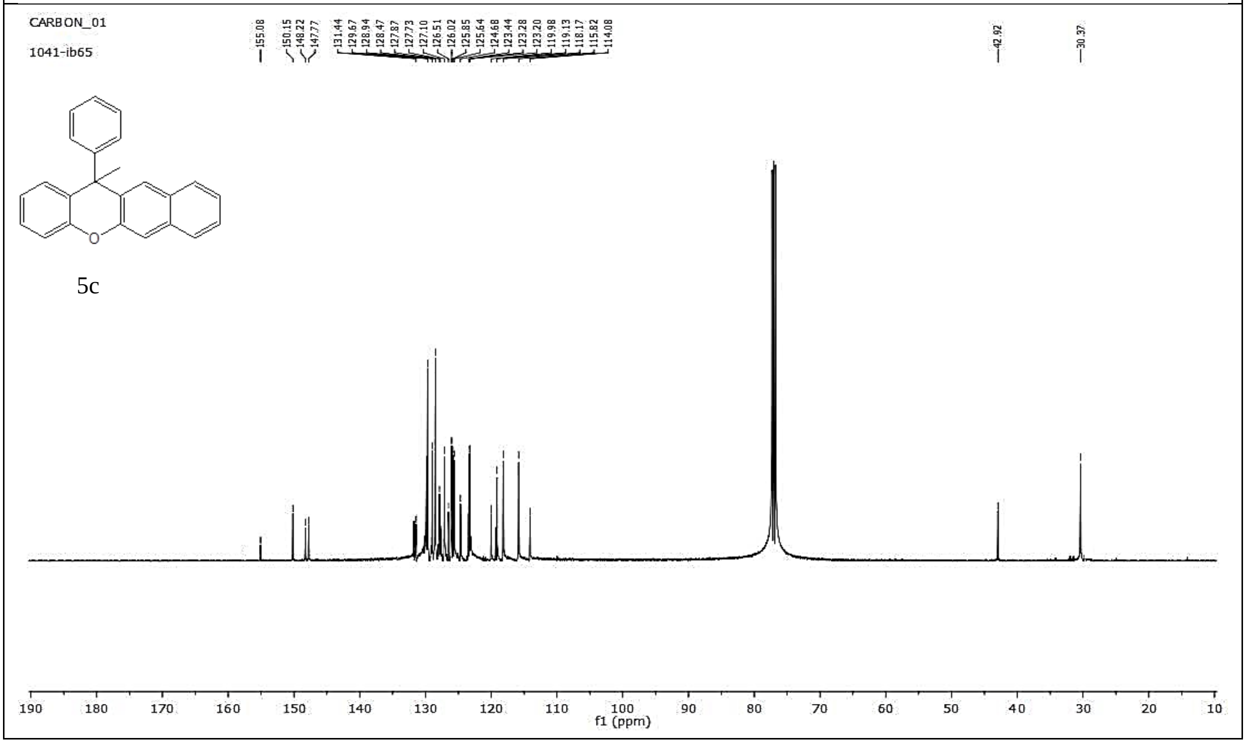
CARBON_01
1041-ib65
155.08
150.15
148.22
147.77
131.44
129.67
128.94
128.47
127.87
127.73
127.10
126.51
126.02
125.85
125.64
124.68
123.44
123.28
123.20
119.98
119.13
118.17
115.82
114.08
42.92
30.37
5c
190 180 170 160 150 140 130 120 110 100 90 80 70 60 50 40 30 20 10
f1 (ppm)

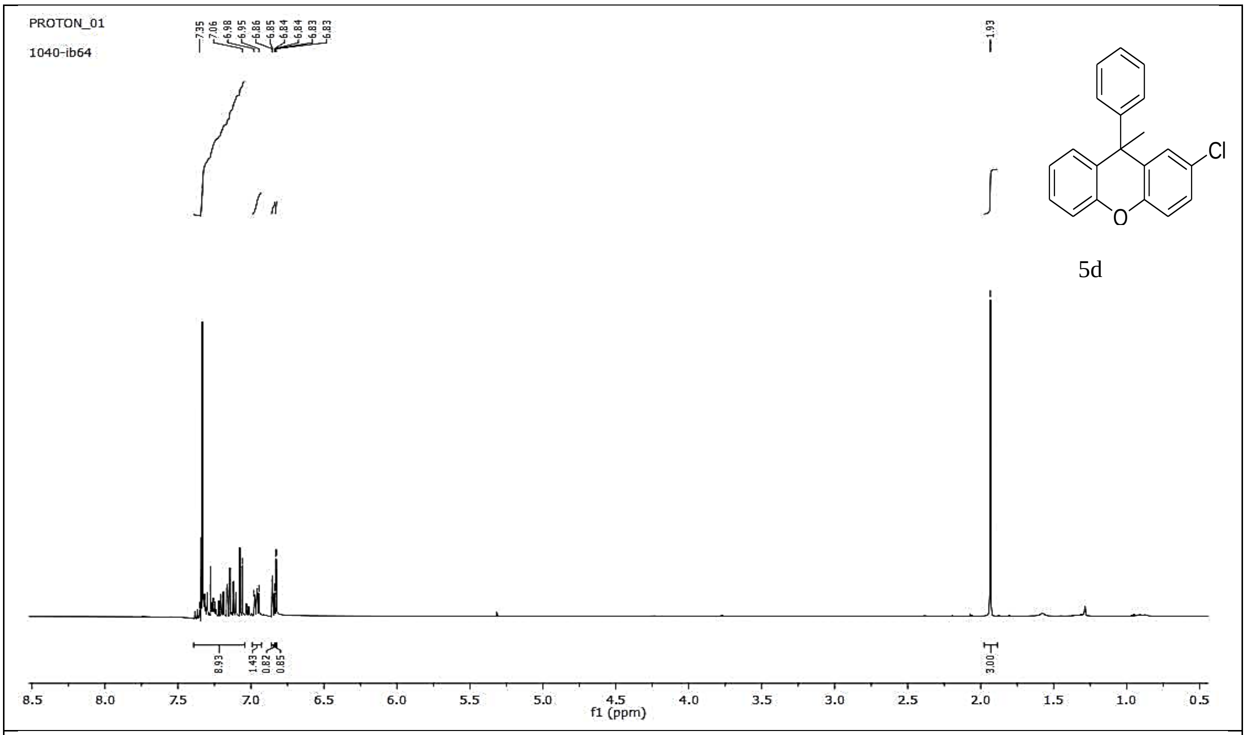
PROTON_01
1040-ib64
7.35
7.06
6.98
6.95
6.86
6.85
6.84
6.84
6.83
6.83
1.93
Cl
O
5d
8.93
1.43
0.82
0.85
3.00
8.5
8.0
7.5
7.0
6.5
6.0
5.5
5.0
4.5
4.0
3.5
3.0
2.5
2.0
1.5
1.0
0.5
f1 (ppm)

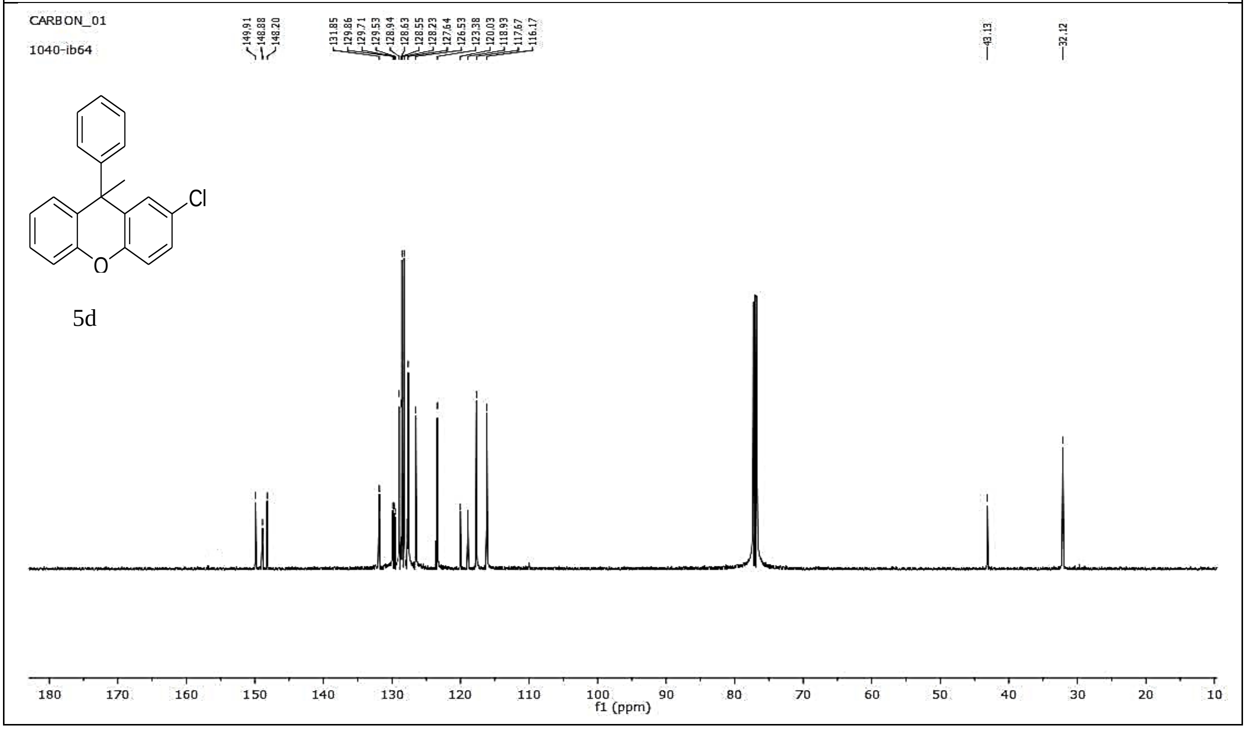
CARBON_01
1040-ib64
149.91
148.88
148.20
131.85
129.86
129.71
129.53
128.94
128.63
128.55
128.23
127.64
126.53
123.38
120.03
118.93
117.67
116.17
43.13
32.12
Cl
O
5d
180
170
160
150
140
130
120
110
100
90
80
70
60
50
40
30
20
10
f1 (ppm)

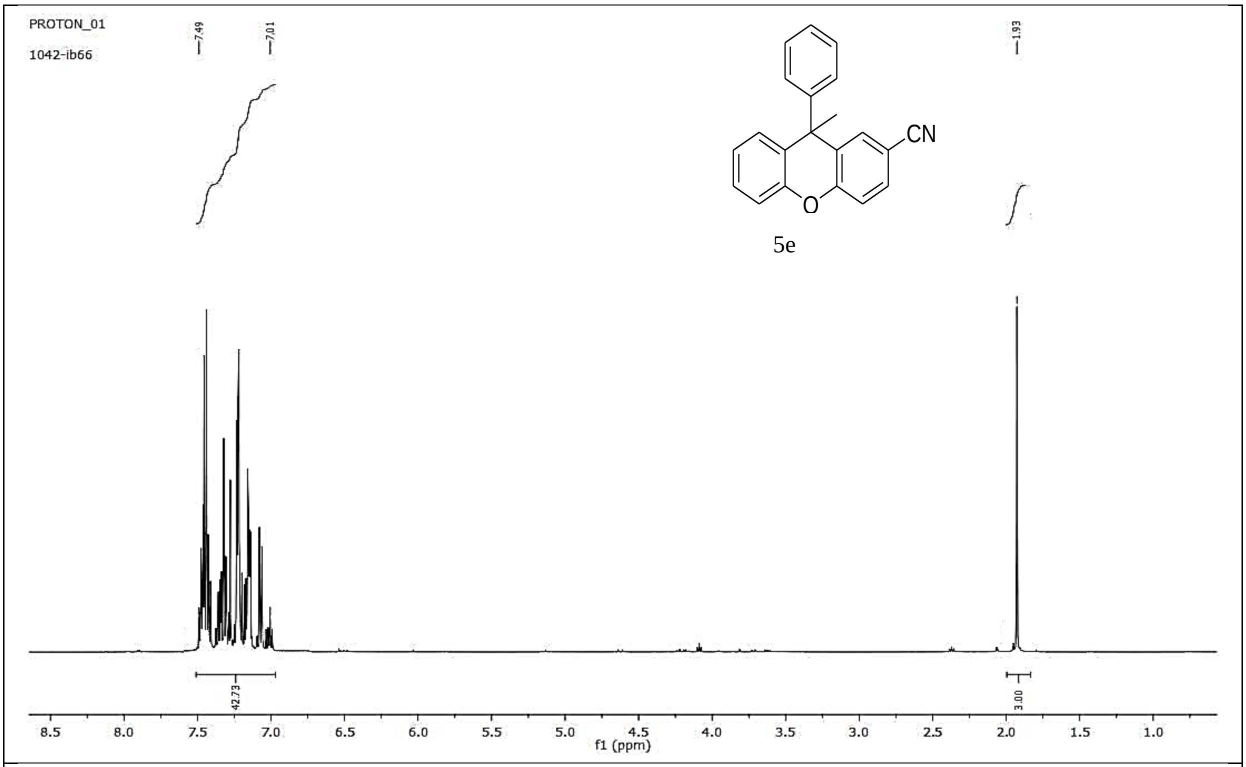
PROTON_01
1042-ib66
7.49
7.01
1.93
CN
O
5e
42.73
3.00
8.5 8.0 7.5 7.0 6.5 6.0 5.5 5.0 4.5 4.0 3.5 3.0 2.5 2.0 1.5 1.0
f1 (ppm)

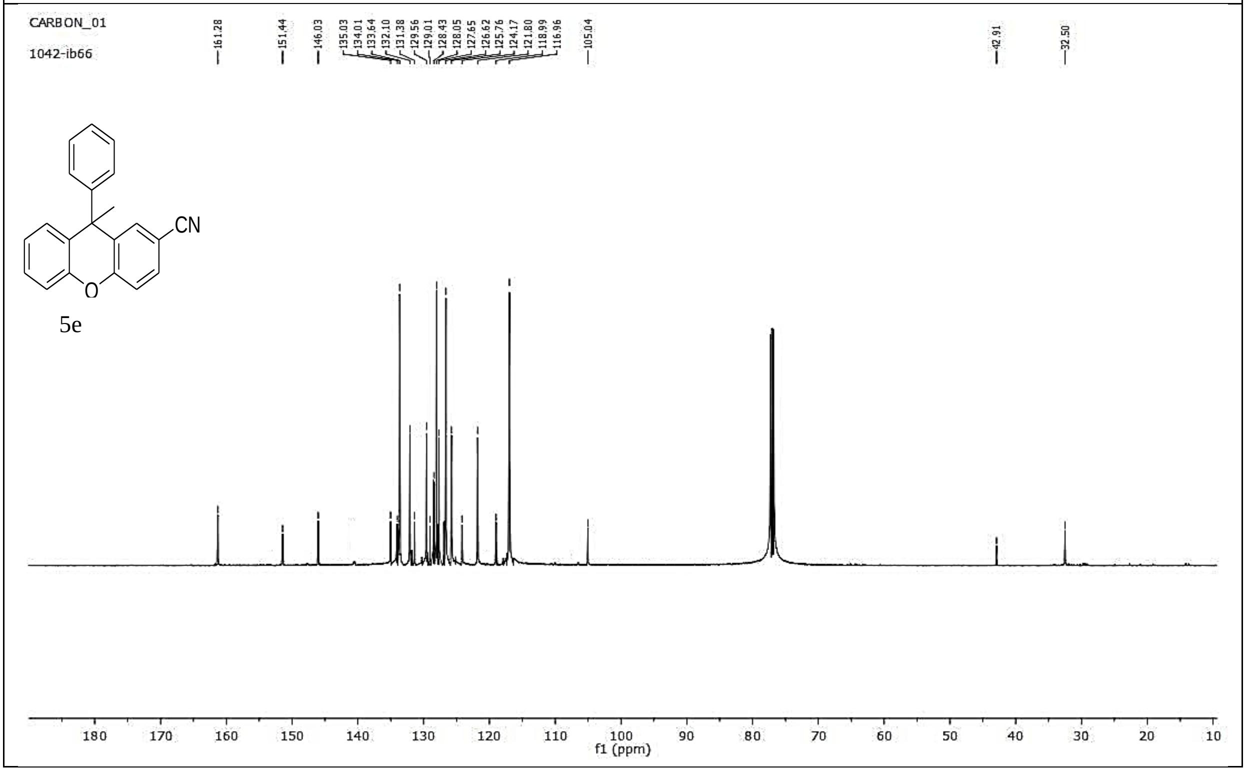
CARBON_01
1042-ib66
161.28
151.44
146.03
135.03
134.01
133.64
132.10
131.38
129.56
129.01
128.43
128.05
127.65
126.62
125.76
124.17
121.80
118.99
116.96
105.04
42.91
32.50
CN
O
5e
180 170 160 150 140 130 120 110 100 90 80 70 60 50 40 30 20 10
f1 (ppm)

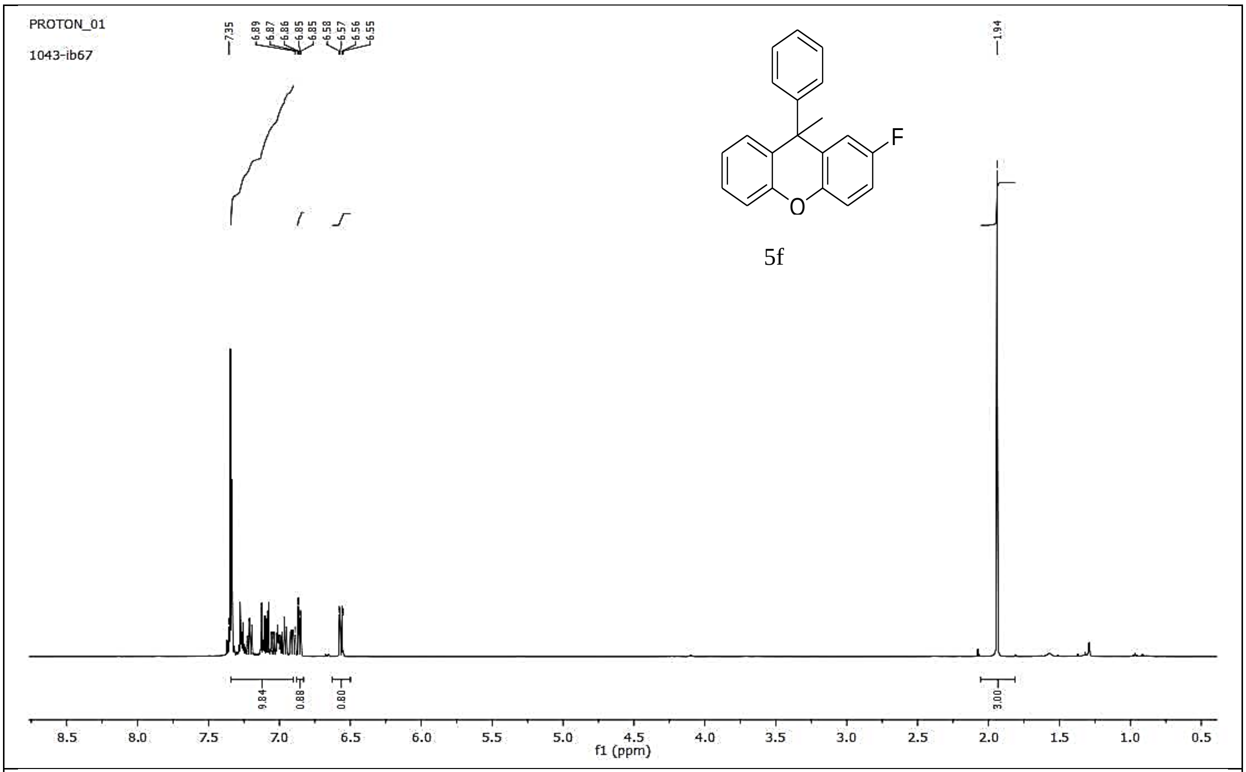
PROTON_01
1043-ib67
7.35
6.89
6.87
6.86
6.85
6.85
6.58
6.57
6.56
6.55
1.94
F
O
5f
9.84
0.88
0.80
3.00
8.5 8.0 7.5 7.0 6.5 6.0 5.5 5.0 4.5 4.0 3.5 3.0 2.5 2.0 1.5 1.0 0.5
f1 (ppm)

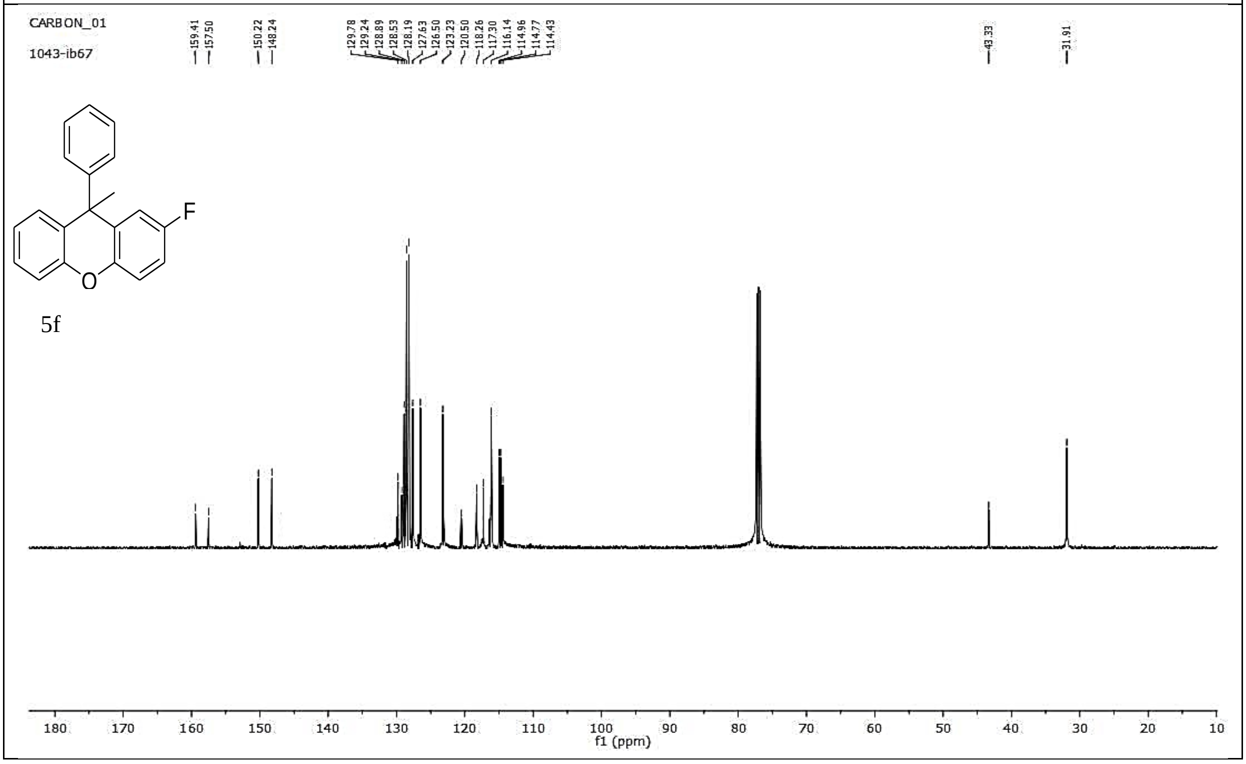
CARBON_01
1043-ib67
159.41
157.50
150.22
148.24
129.78
129.24
128.89
128.53
128.19
127.63
126.50
123.23
120.50
118.26
117.30
116.14
114.96
114.77
114.43
43.33
31.91
F
O
5f
180 170 160 150 140 130 120 110 100 90 80 70 60 50 40 30 20 10
f1 (ppm)

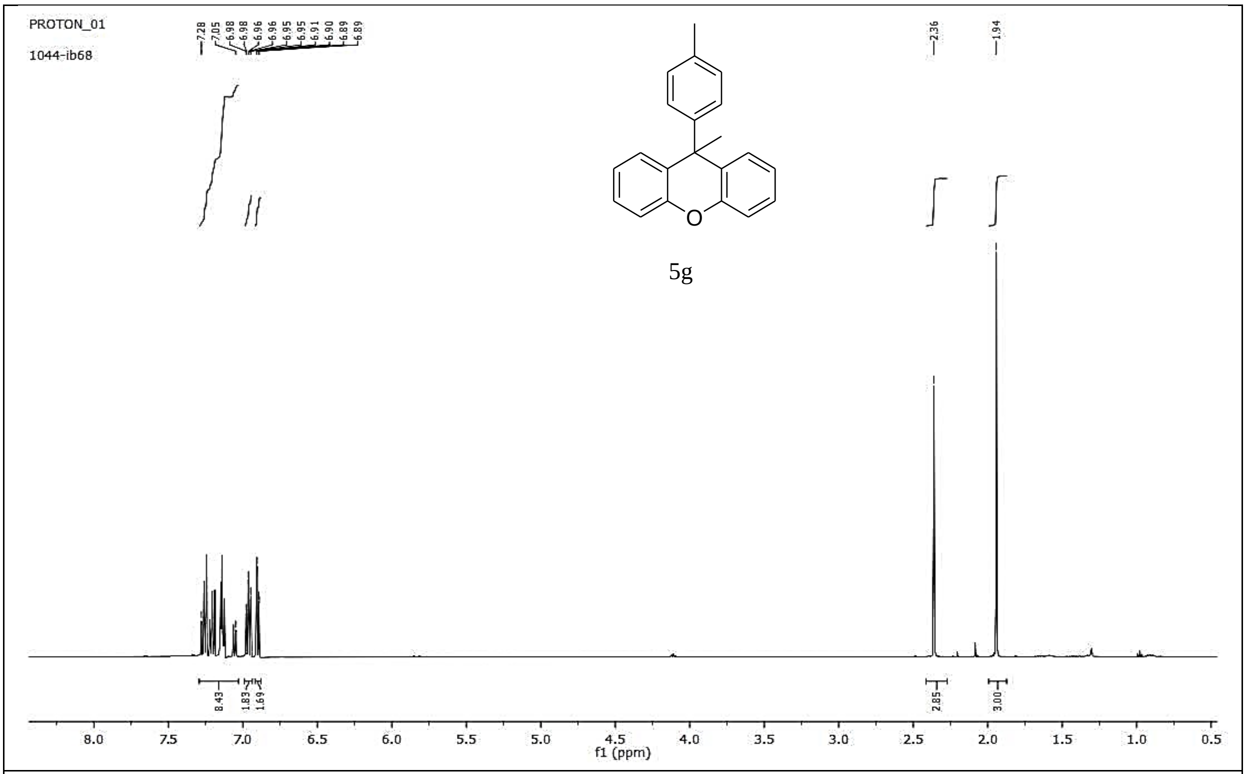
PROTON_01
1044-ib68
7.28
7.05
6.98
6.98
6.96
6.96
6.95
6.95
6.91
6.90
6.89
6.89
2.36
1.94
O
5g
8.43
1.83
1.69
2.85
3.00
8.0 7.5 7.0 6.5 6.0 5.5 5.0 4.5 4.0 3.5 3.0 2.5 2.0 1.5 1.0 0.5
f1 (ppm)

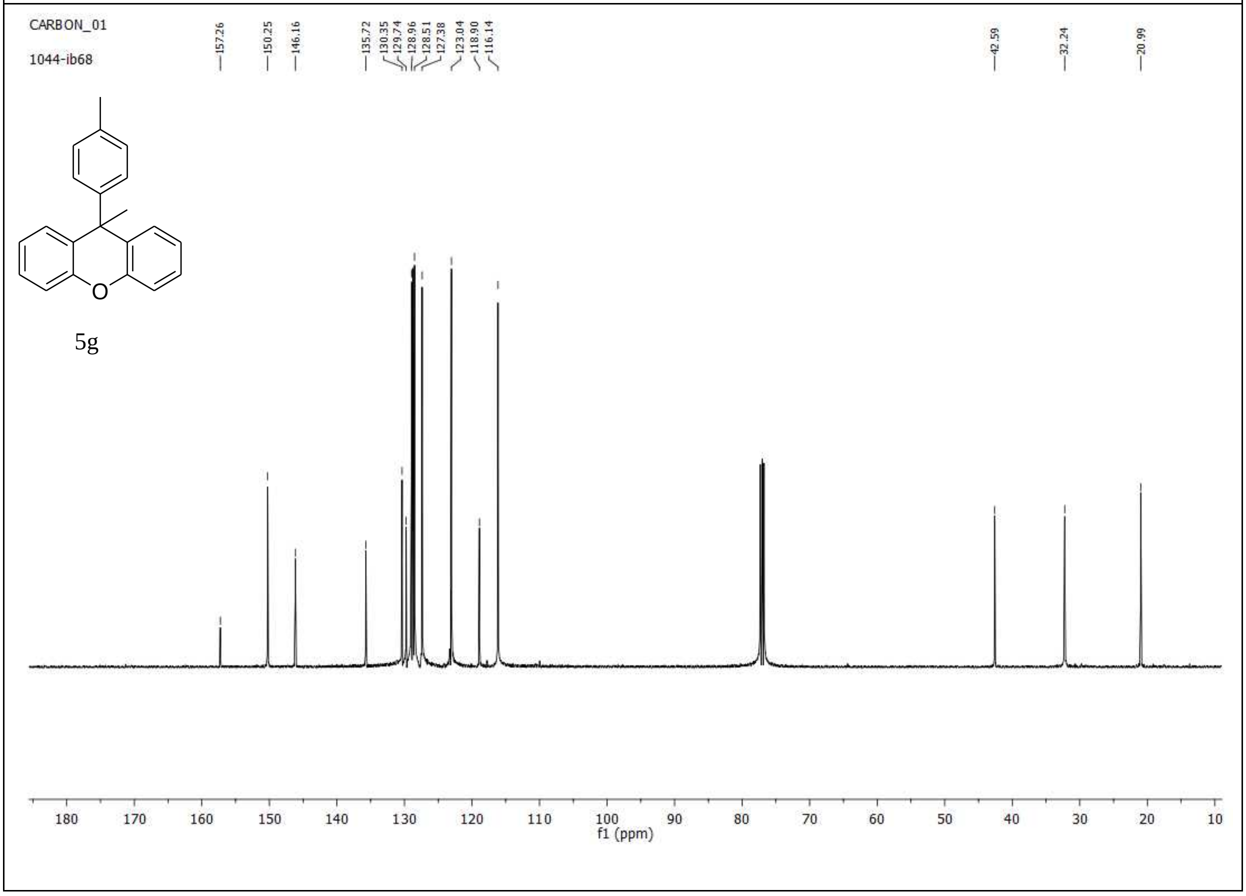
CARBON_01
1044-ib68
157.26
150.25
146.16
135.72
130.35
129.74
128.96
128.51
127.38
123.04
118.90
116.14
42.59
32.24
20.99
O
5g
180 170 160 150 140 130 120 110 100 90 80 70 60 50 40 30 20 10
f1 (ppm)

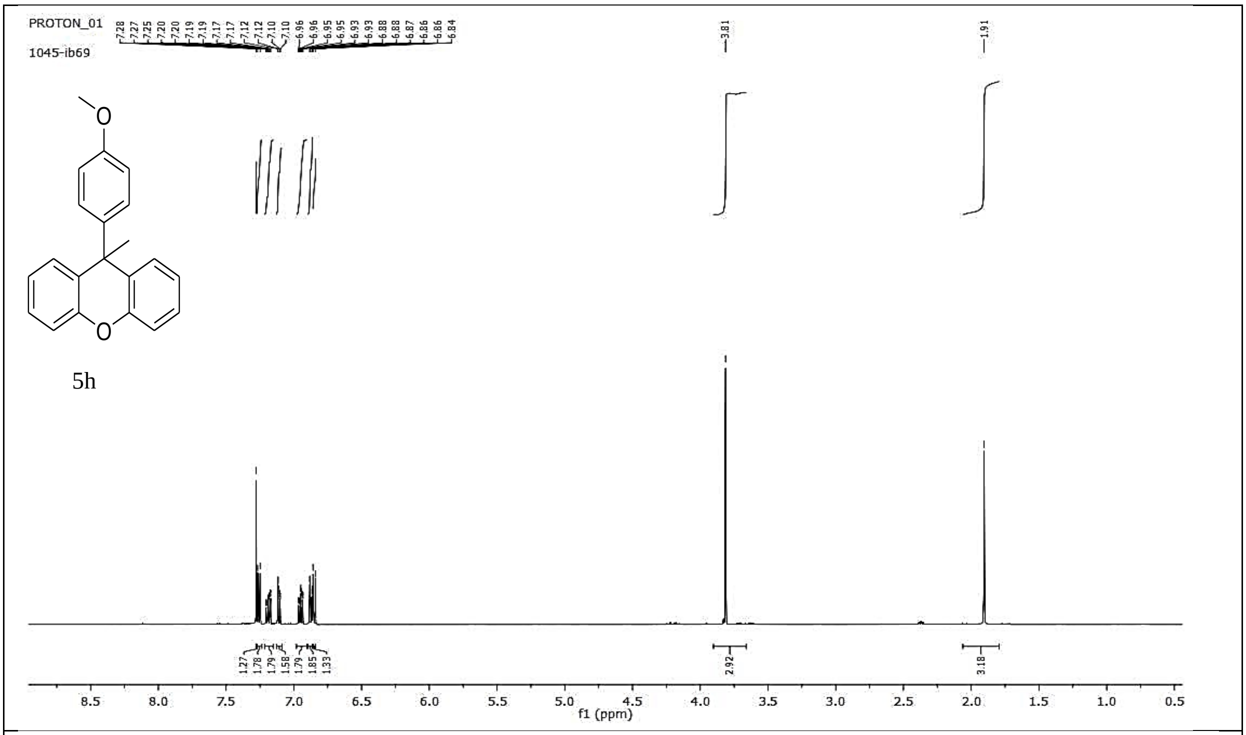
PROTON_01
1045-ib69
7.28 7.27 7.25 7.20 7.20 7.19 7.19 7.17 7.17 7.12 7.12 7.10 7.10 6.96 6.96 6.95 6.95 6.93 6.93 6.88 6.88 6.87 6.86 6.86 6.84
3.81
1.91
O
O
5h
1.27 1.78 1.79 1.58 1.79 1.85 1.33
2.92
3.18
8.5 8.0 7.5 7.0 6.5 6.0 5.5 5.0 4.5 4.0 3.5 3.0 2.5 2.0 1.5 1.0 0.5
f1 (ppm)

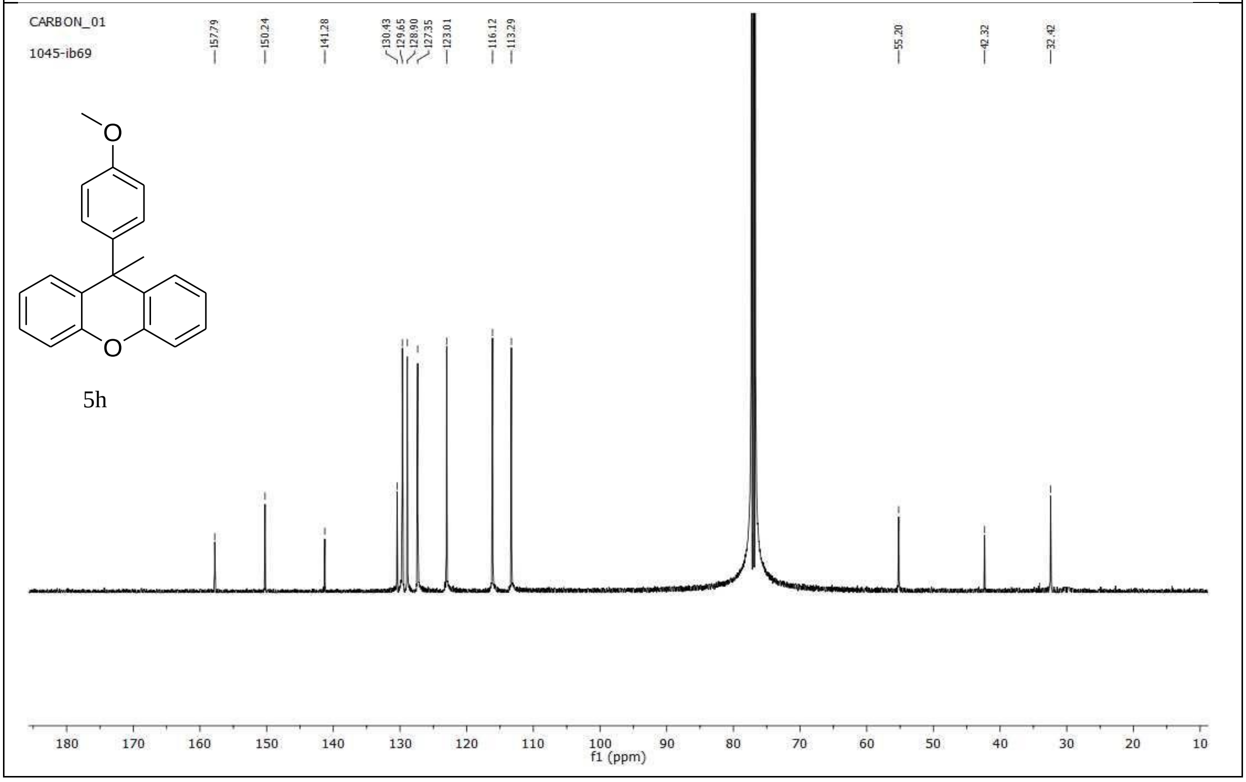
CARBON_01
1045-ib69
157.79
150.24
141.28
130.43 129.65 128.90 127.35
123.01
116.12
113.29
55.20
42.32
32.42
O
O
5h
180 170 160 150 140 130 120 110 100 90 80 70 60 50 40 30 20 10
f1 (ppm)

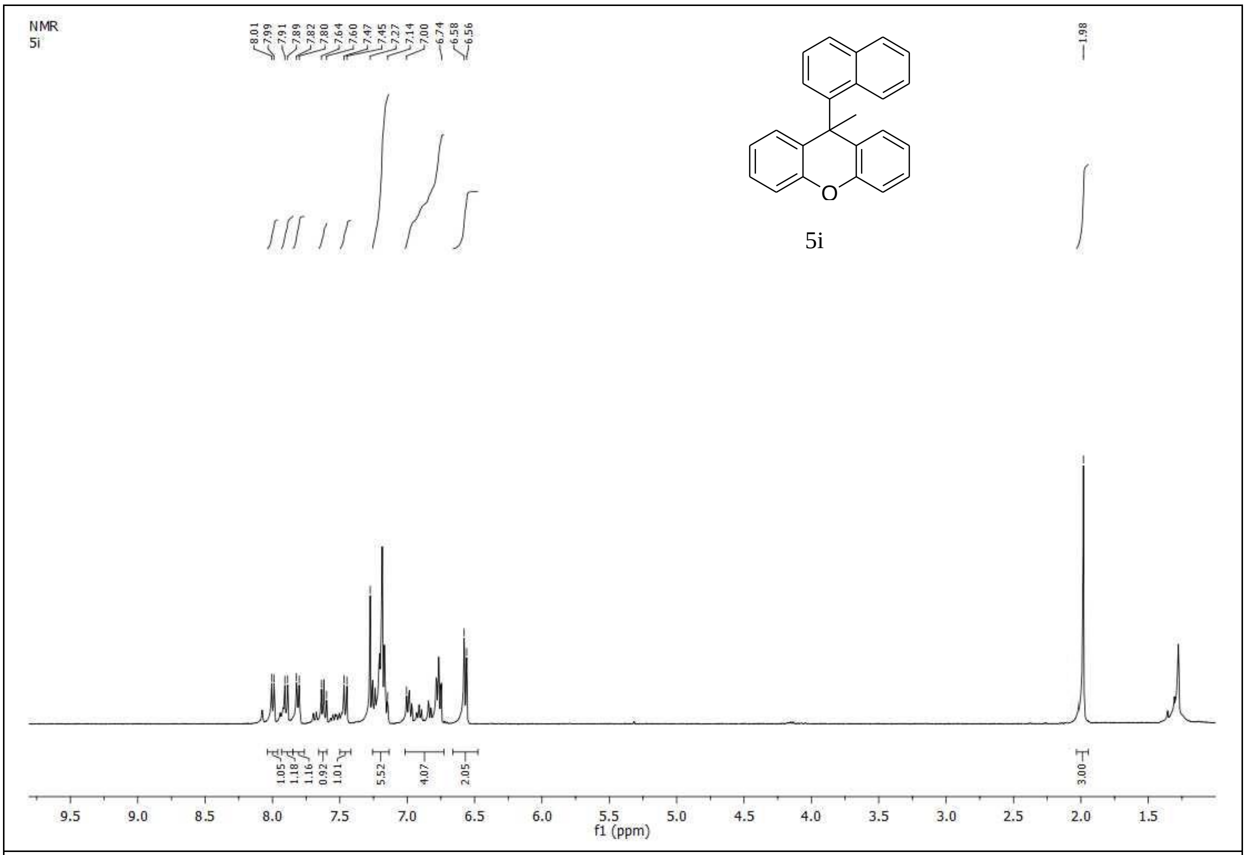
NMR
5i
8.01 7.99 7.91 7.89 7.82 7.80 7.64 7.60 7.47 7.45 7.27 7.14 7.00 6.74 6.58 6.56
1.98
O
5i
1.05 1.18 1.16 0.92 1.01 5.52 4.07 2.05 3.00
9.5 9.0 8.5 8.0 7.5 7.0 6.5 6.0 5.5 5.0 4.5 4.0 3.5 3.0 2.5 2.0 1.5
f1 (ppm)

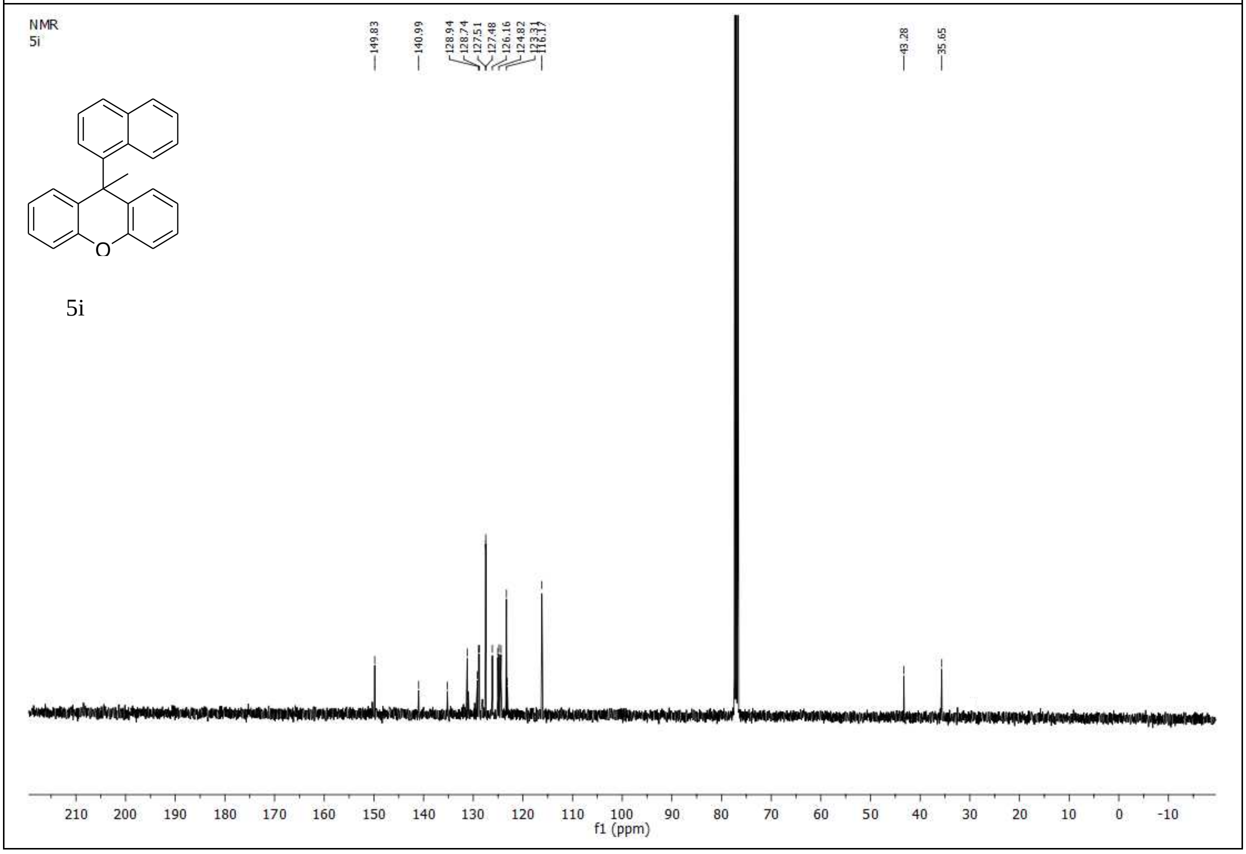
NMR
5i
149.83 140.99 128.94 128.74 127.51 127.48 126.16 124.82 123.31 116.17
43.28 35.65
O
5i
210 200 190 180 170 160 150 140 130 120 110 100 90 80 70 60 50 40 30 20 10 0 -10
f1 (ppm)

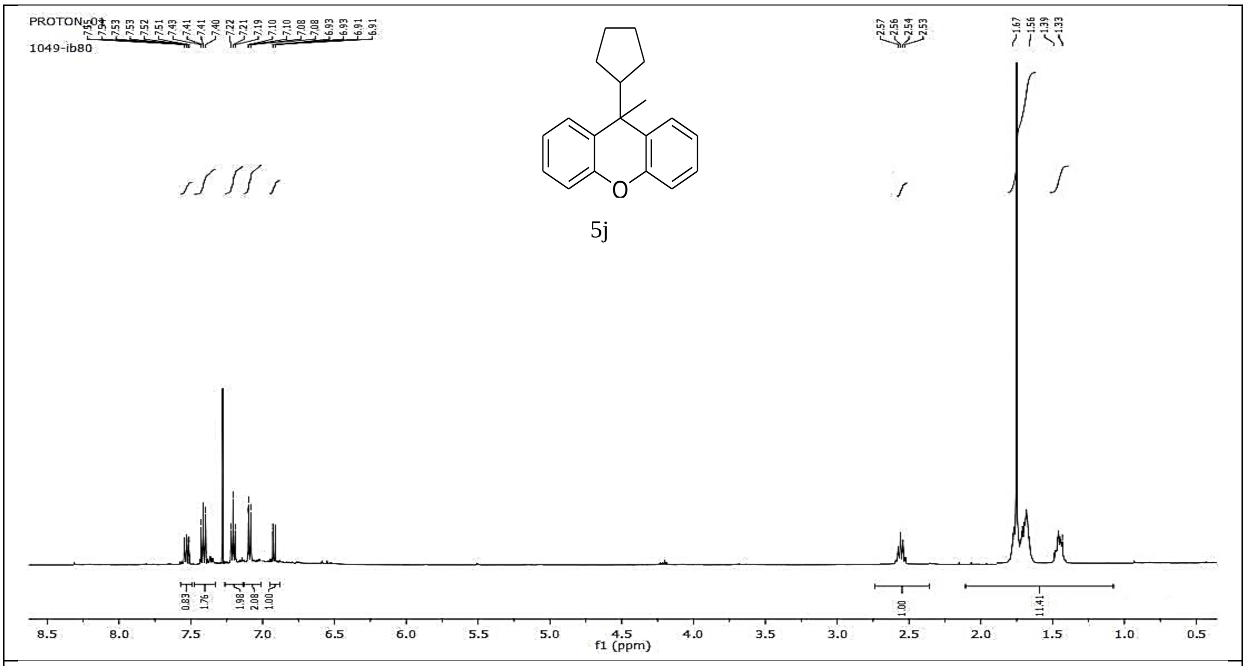
PROTON_01
1049-ib80
2.57
2.56
2.54
2.53
1.67
1.56
1.39
1.33
O
5j
0.83
1.76
1.98
2.08
1.00
1.00
11.41
f1 (ppm)

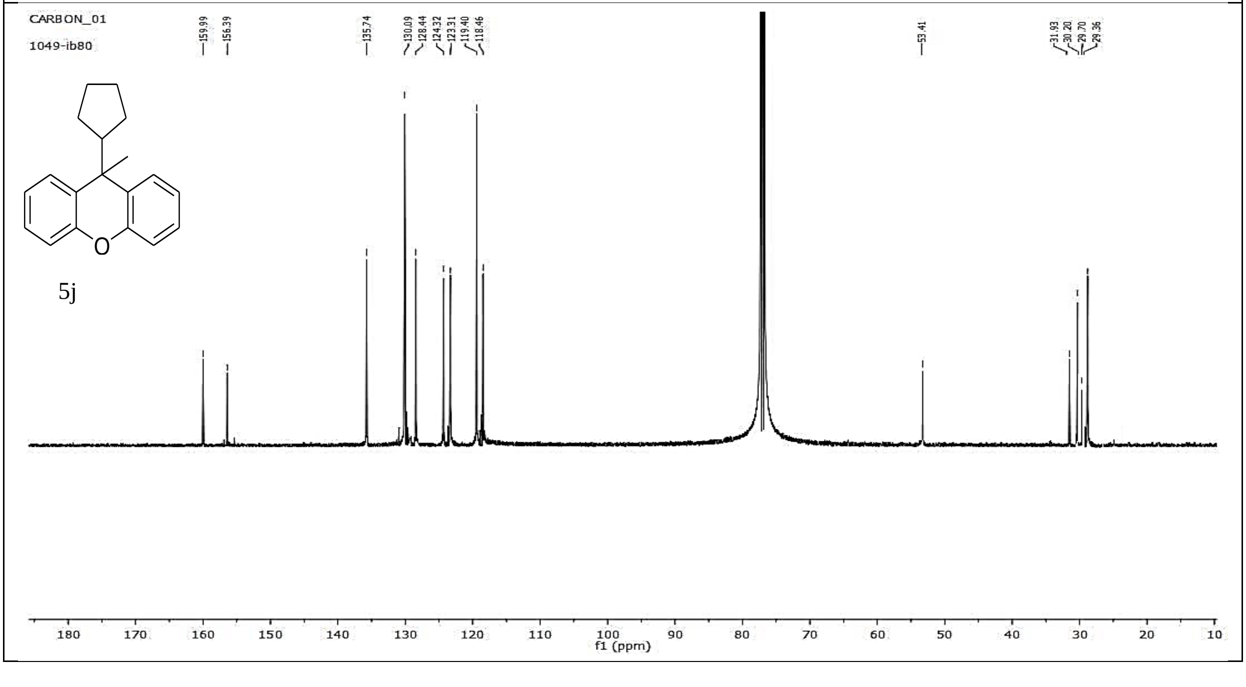
CARBON_01
1049-ib80
159.99
156.39
135.74
130.09
128.44
124.32
123.31
119.40
118.46
53.41
31.93
30.20
29.70
29.36
O
5j
f1 (ppm)

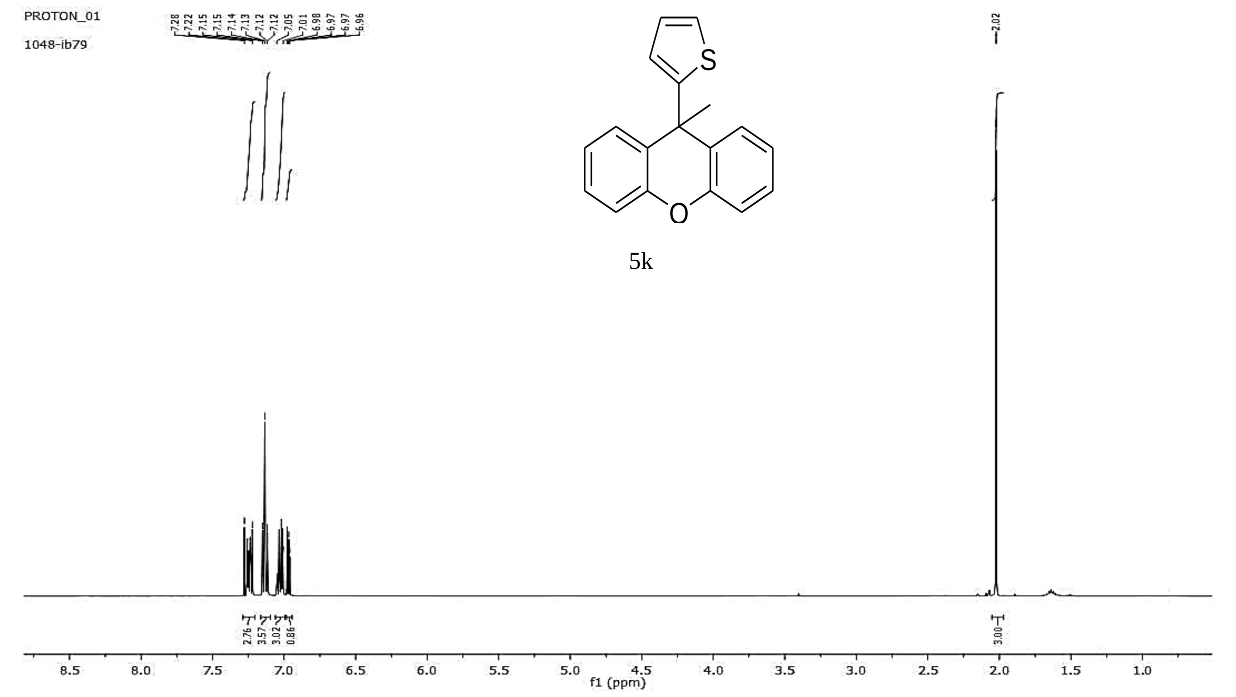
PROTON_01
1048-ib79
7.28
7.22
7.15
7.15
7.14
7.13
7.12
7.12
7.05
7.01
6.98
6.97
6.97
6.96
2.02
S
O
2.76
3.57
3.02
0.86
3.00
8.5
8.0
7.5
7.0
6.5
6.0
5.5
5.0
4.5
4.0
3.5
3.0
2.5
2.0
1.5
1.0
f1 (ppm)

5k

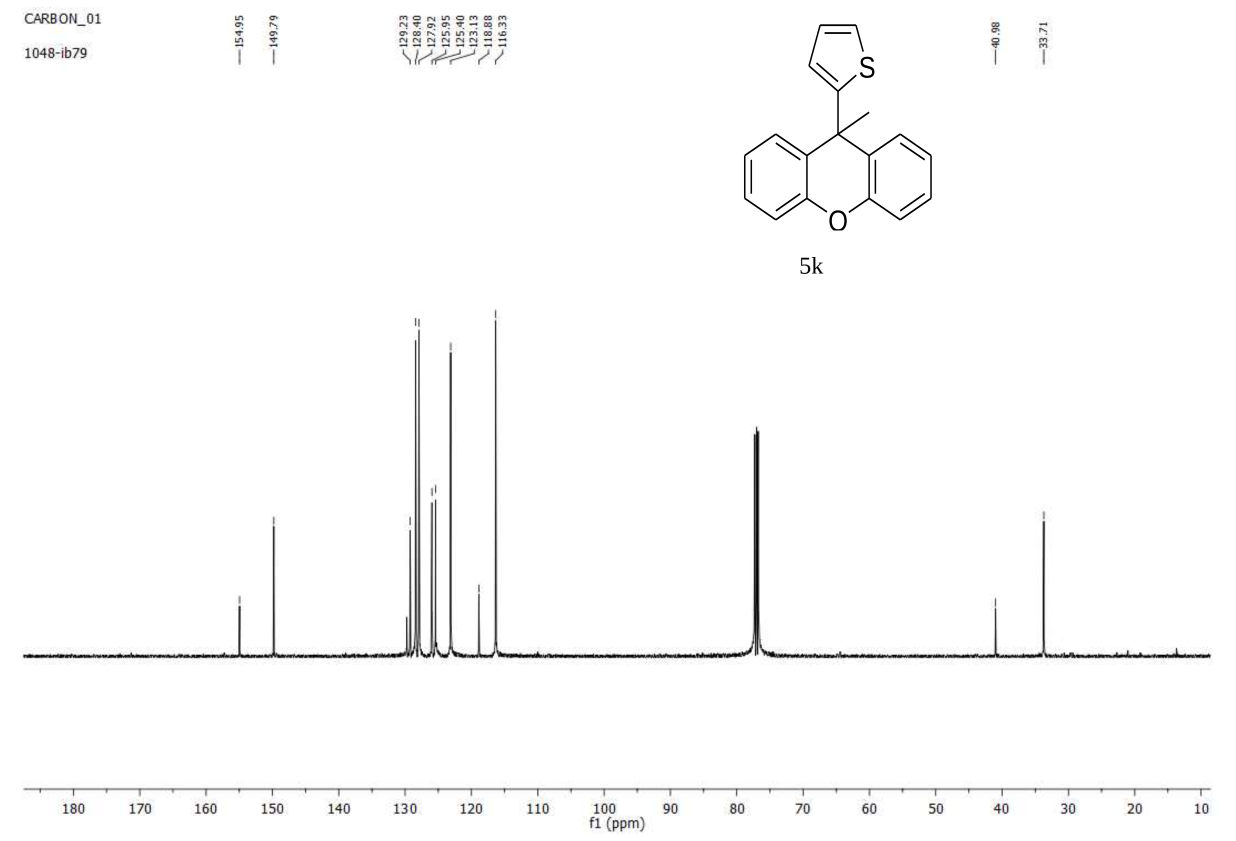
CARBON_01
1048-ib79
154.95
149.79
129.23
128.40
127.92
125.95
125.40
123.13
118.88
116.33
40.98
33.71
S
O
180
170
160
150
140
130
120
110
100
90
80
70
60
50
40
30
20
10
f1 (ppm)

5k

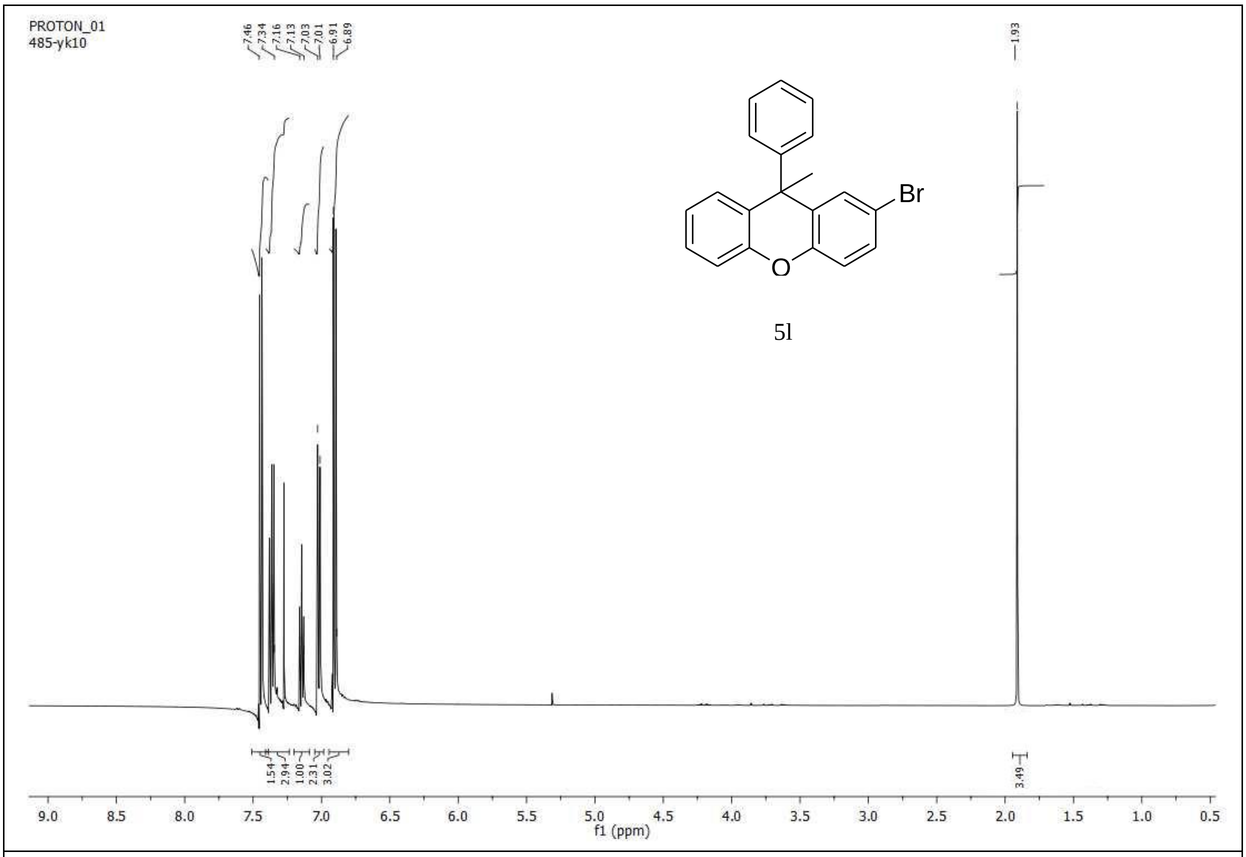
PROTON_01
485-yk10
7.46
7.34
7.16
7.13
7.03
7.01
6.91
6.89
1.93
Br
O
5l
1.54
2.94
1.00
2.31
3.02
3.49
9.0 8.5 8.0 7.5 7.0 6.5 6.0 5.5 5.0 4.5 4.0 3.5 3.0 2.5 2.0 1.5 1.0 0.5
f1 (ppm)

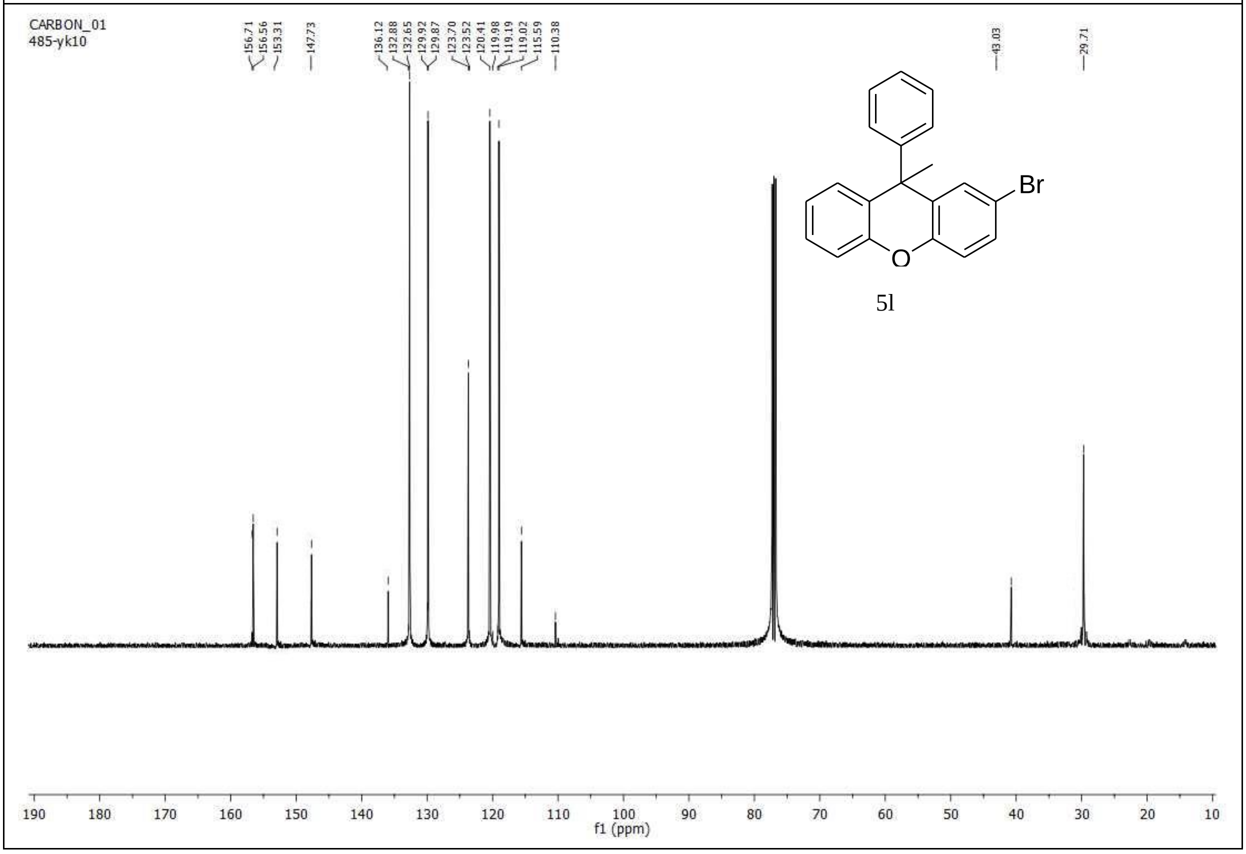
CARBON_01
485-yk10
156.71
156.56
153.31
147.73
136.12
132.88
132.65
129.92
129.87
123.70
123.52
120.41
119.98
119.19
119.02
115.59
110.38
43.03
29.71
Br
O
5l
190 180 170 160 150 140 130 120 110 100 90 80 70 60 50 40 30 20 10
f1 (ppm)
